# Robust automated backbone triple resonance NMR assignments of proteins using Bayesian-based simulated annealing

**DOI:** 10.1038/s41467-023-37219-z

**Published:** 2023-03-21

**Authors:** Anthony C. Bishop, Glorisé Torres-Montalvo, Sravya Kotaru, Kyle Mimun, A. Joshua Wand

**Affiliations:** 1 Department of Biochemistry & Biophysics, Texas A&M University, College Station, TX 77843 USA; 2 Graduate Group in Biochemistry & Molecular Biophysics, Perelman School of Medicine, University of Pennsylvania, Philadelphia, PA 19014 USA; 3 Department of Chemistry, Texas A&M University, College Station, TX 77843 USA; 4 Department of Molecular & Cellular Medicine, Texas A&M University, College Station, TX 77843 USA

**Keywords:** Solution-state NMR, Molecular conformation, Software

## Abstract

Assignment of resonances of nuclear magnetic resonance (NMR) spectra to specific atoms within a protein remains a labor-intensive and challenging task. Automation of the assignment process often remains a bottleneck in the exploitation of solution NMR spectroscopy for the study of protein structure-dynamics-function relationships. We present an approach to the assignment of backbone triple resonance spectra of proteins. A Bayesian statistical analysis of predicted and observed chemical shifts is used in conjunction with inter-spin connectivities provided by triple resonance spectroscopy to calculate a pseudo-energy potential that drives a simulated annealing search for the most optimal set of resonance assignments. Termed Bayesian Assisted Assignments by Simulated Annealing (BARASA), a C++ program implementation is tested against systems ranging in size to over 450 amino acids including examples of intrinsically disordered proteins. BARASA is fast, robust, accommodates incomplete and incorrect information, and outperforms current algorithms – especially in cases of sparse data and is sufficiently fast to allow for real-time evaluation during data acquisition.

## Abstract (web summary)

The authors present BARASA, an approach to assign backbone triple resonance spectra of proteins that augments traditional approaches with a Bayesian statistical analysis of the observed chemical shifts. The algorithm employs a simulated annealing engine to establish a consensus set of resonance assignments and is tested against systems ranging in size to over 450 amino acids including examples of intrinsically disordered proteins.

## Introduction

Nuclear magnetic resonance (NMR) spectroscopy is unique in its ability to provide simultaneous and comprehensive structural and dynamical atomic-scale information about macromolecules such as proteins in solution^1–4^. Unfortunately, however, an observed resonance frequency in an NMR spectrum cannot yet be directly assigned to the individual atom(s) within the protein from which they arise without the time-intensive collection and analysis of additional spectra. Comprehensive mapping of individual resonances comprising nuclear magnetic resonance (NMR) spectra to specific atoms within a protein molecule is a general prerequisite for the successful analysis of the structure and dynamics of proteins by NMR spectroscopy. Early applications of multi-dimensional homonuclear ^1^H NMR data to the so-called resonance assignment problem relied heavily on human intervention. The first comprehensive approach was the sequential assignment method, which centered on identification of J-coupled spin systems^5^ that are then assembled through connections provided by short distances revealed by the nuclear Overhauser effect (NOE) interactions between sequential residues using the identity of side chains to error-check against the primary structure^6,7^. The subsequent main chain directed (MCD) assignment strategy^8,9^ formalized self-correcting cyclic patterns of backbone ^1^H-^1^H NOE interactions and provided a more robust algorithmic framework that relieved somewhat the complexity of identifying side chain resonances^10,11^. While the MCD approach did lead to the first fully automated assignment of ^1^H resonances to backbone hydrogens^11^, automation of ^1^H-based resonance assignments was generally frustrated by the overwhelming spectral degeneracy of multidimensional ^1^H spectra of proteins and the interference of technical attributes such as a prominent diagonal. The introduction of heteronuclear triple resonance spectroscopy^12–17^ completely changed the landscape of the resonance assignment task by providing much greater resolution, generally higher quality data, and, most importantly, definitive rules with very precise meanings for making connectivities (correlations) between backbone resonances. Triple resonance assignments of the protein backbone permit access, either directly or by tethering to side chain resonance assignments, to a wide range of dynamic phenomena^17,18^ and structural information^19–21^.

Automated triple resonance algorithms have led to effectively complete backbone resonance assignments of smaller proteins with little human intervention and greatly aided the assignment of larger systems^22–24^. Yet, even with the advent of transverse relaxation optimized spectroscopy (TROSY)^25^, the comprehensive assignment of systems larger than 30 kDa remains remarkably rare. The limitations are quite analogous to that summarized for earlier assignment strategies based exclusively on ^1^H-^1^H scalar and NOE interactions: increasing ambiguity in connectivities due to degeneracy, loss of resonances due to relaxation or artifact, and other confounding spectral attributes are simply not sufficiently accommodated by current automated assignment strategies.

Here, we strive to overcome the issue of data sparseness and ambiguity by appealing to the statistics of Bayes to utilize available information more effectively via the calculation of explicit probabilities. Importantly, this formalism also allows for a flexible and adaptable incorporation of chemical shift prediction and structural knowledge into the assignment process. By implementing the Bayesian analysis within a simulated annealing engine, we develop a robust and efficient search for optimal solutions. Protein assignment algorithms utilizing simulated annealing have been developed in the past^26^. However, the stochastic algorithm described here takes advantage of readily available pre-existing structural models, both experimentally-determined and predicted, and in doing so more effectively exploits the rich information contained within structure-based predicted chemical shifts. We demonstrate how these invaluable restraints greatly aid the resonance assignment process, especially in cases where data may be otherwise sparse or even incorrect. We also compare the overall performance of BARASA against three highly cited assignment algorithms on a variety of experimental datasets.

## Results and discussion

### Bayesian assisted resonance assignments by simulated annealing (BARASA)

We designed an algorithm, termed BARASA, which utilizes a simulated annealing approach^27^ to efficiently search the immense solution space for the optimal set of resonance assignments starting with a set of raw crosspeaks derived from triple resonance type spectra. The objective is to find the correct mapping of individual resonances to specific atoms within the protein molecule. The algorithm first assembles an initial set of spin systems based on an analysis of crosspeak lists and the connectivity rules of the particular triple resonance experiments employed. This process may not yield an unambiguous nor complete set of spin systems due to inherent degeneracy and missing or artifactual peaks (See Methods). As a result, a given crosspeak could be associated with multiple, spectrally-overlapping spin systems; in which case, the crosspeak is randomly placed in one of the overlapping spin systems. The simulated annealing search engine then randomly distributes the starting set of spin systems to specific residue positions. If there are more spin systems than residue positions, then the excess spin systems are placed in a cache for later use as described below. The energy of this initial state is calculated as the sum of the energies of the individual spin systems currently placed in residue positions. Each spin system energy is composed of two terms: the adjacency energy and the chemical shift energy. The adjacency energy describes the interaction between two spin systems mapped to adjacent locations on the amino acid sequence. This energy is minimized if the Cα(i), Cβ(i), and C’(i) shifts of the spin system match the Cα(i-1), Cβ(i-1), and C’(i-1) of the spin system at the following residue in the sequence. In contrast, the chemical shift energy describes the interaction between a spin system and its current residue position i.e., it is defined by the local sequence and structure. This energy is minimized when the resonances of the spin system closely match the predicted values of the current residue position, while also failing to match the predicted values at all other residue positions. Application of Bayes’ theorem then provides a posterior probability of assigning each spin system at each location in the sequence that is based on the predicted and experimental shifts. Using this probability, the chemical shift energy is calculated (see Methods for a more detailed description). After the initial calculation of energy, a spin system or individual crosspeak is randomly chosen. A spin system is either moved to an unoccupied residue position, swapped with another spin system, or added to the cache. Spin systems or cross peaks deposited to their respective caches have no priority and are randomly selected from the cache. Similarly, if a chosen crosspeak can be productively added to the crosspeak cache, swapped with another crosspeak in an overlapping spin system, or moved to an overlapping spin system, the move is made. With every crosspeak/spin system swap, the decision to accept the proposed move is made based on the energy of the system before and after the proposed swap. Using an effective temperature T, the Metropolis criterion^28^ is applied (Eq. 1).

1 $${P}_{{{{{\rm{accept}}}}}}={{{{{\rm{min}}}}}} \left(1,{{{{{\rm{exp }}}}}}(-\triangle E/T)\right)$$

**Fig. 1:**
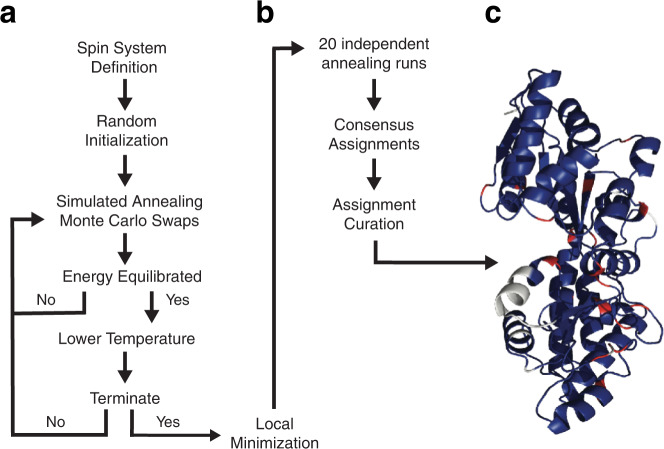
Outline of the BARASA triple resonance assignment algorithm. **a** The search engine rests on a Bayesian-based simulated annealing protocol that uses a specific-heat mechanism to guide cooling. Crosspeaks lists drawn from triple resonance spectra are assembled into putative spin systems, which are then randomly assigned to positions within the primary sequence of the protein. Sequential adjacency in the primary sequence is provided by apparent connectivities derived from triple resonance NMR spectra. Predicted chemical shifts, based on a high-resolution structural model or gleaned from empirical amino acid-specific distributions, are incorporated into the system energy using Bayesian statistics. Throughout annealing, crosspeaks may move among spin systems with overlapping resonances, changing the energies of the affected spin systems. Annealing involves Monte Carlo swapping of both crosspeak assignments to spin systems and spin system assignments to locations in the sequence. The concept of dynamic swapping of individual crosspeaks or entire spin systems is outlined in Fig. 2. Annealing continues until energy equilibration is achieved. The temperature is then lowered and the system re-equilibrated. Annealing is stopped when the termination criteria are met and a local minimization routine is performed. **b** The final resonance assignments are developed from results of multiple independent simulated annealing runs. **c** Shown is a ribbon representation of maltose binding protein (PDB code: 1DMB [10.2210/pdb1DMB/pdb]) color-coded according to assignment status following analysis by BARASA: correctly assigned residues (blue); unassigned residues (white), prolines (red). See main text for further details.

**Fig. 2:**
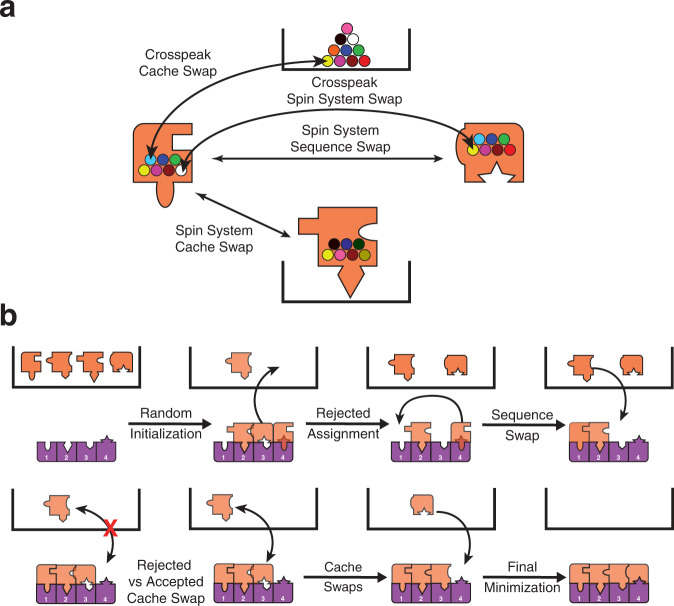
Dynamic assembly of spin systems during simulated annealing. **a** Spin systems (orange puzzle pieces) begin in the cache (black box) and are initialized by random assignment to the sequence (purple pieces). Spin systems can then be swapped with others or moved to different locations of the sequence or to the cache. Spin systems or cross peaks in their respective caches have no priority and are randomly selected. Swaps are accepted or rejected with a probability based on the change in energy of the proposed swap. **b** The energy of each spin system depends on how it fits with the adjacent spin system (adjacency energy) and with the predicted shifts for that residue location (chemical shift energy). Exchange of crosspeaks between spin systems can be thought of as changing the puzzle piece shape. See main text and supplementary material for details.

${P}_{{{{{\rm{accept}}}}}}$ is the probability of accepting the swap and $\varDelta E$ is the change in energy due to the proposed swap. If $\varDelta E\le 0$ then ${P}_{{{{{\rm{accept}}}}}}$ is set to 1. If $\varDelta E$ > 0, then $0 < \,{P}_{{{{{\rm{accept}}}}}} < 1$ and a uniformly distributed random number *r* such that $0\le {r}\le 1$ is generated. If $r\le \,{P}_{{{{{\rm{accept}}}}}}$ then the swap is accepted. Otherwise, the swap is rejected and the system state is left unchanged. Random swap attempts are continued until the average energy of system does not vary significantly. $T$ is then decreased by following a highly optimized schedule based on a quantity analogous to the specific heat of the system (see Methods). The system is further cooled and equilibrated in this manner until a set of termination criteria are achieved and the annealing protocol is ended. Finally, to ensure that the system has reached a minimum in energy, a proposed swap of each spin system with every other spin system as well as every crosspeak with every other possible crosspeak is then attempted with only decreasing energy changes being accepted. This post-annealing minimization routine is repeated 100 times. The entire procedure, starting from initialization and ending with minimization, is repeated 20 times. The algorithm then chooses the spin system that was assigned to each residue location in a majority of the annealing runs (if any) and builds a consensus assignment set. The consensus assignment set is further curated using criteria defined below to produce the final assignment set. The overall BARASA algorithm is outlined in Figs. 1 and 2.

### BARASA is accurate, robust, and fast

We tested BARASA against a test set of six different folded protein systems ranging in size and topology: human interleukin-1 receptor antagonist C66A, C122A (IL-1Ra, 152 residues, 17.1 kDa), human interleukin-1*β* (IL-1*β*, 154 residues, 17.5 kDa), *S. solfataricus* indole-3-glycerol phosphate synthase R43S (IGPS, 248 residues, 28.4 kDa), *E. coli* maltose binding protein (MBP, 371 residues, 40.8 kDa), the first cyclization domain from the *Y. pestis* yersiniabactin non-ribosomal peptide synthetase (Cy1, 453 residues, 51.9 kDa), and *E. coli* thymidylate synthase (ecTS, 264 residues, 61.0 kDa homodimer). In addition, we challenged the algorithm with two so-called intrinsically disordered proteins (IDPs). These include the V5 domain (residues 606-672) of human protein kinase C (V5dm, 68 residues, 7.7 kDa) and the intrinsically disordered region of human ANP32A (hIDD, 110 residues, 12.8 kDa). All crosspeak lists were derived from triple resonance data (Table 1). Crosspeak positions used were pulled from the canonical triple resonance spectra used for protein assignment (i.e., HSQC, HNCO^29^, HN(CA)CO^30^, HNCA^31^, HN(CO)CA^31^,HNCACB^32^, HN(CO)CACB/CBCA(CO)NH^33^) (see Supplementary Table S1) with the exception of hIDD in which crosspeaks were derived from provided spin systems. To generate crosspeaks from the spin systems of hIDD, Gaussian error was added to the resonance values to create the chemical shifts of simulated crosspeaks. (see Methods). Four of the data sets (IL-1Ra, IL-1*β*, IGPS, and MBP) were obtained in our laboratory. Crosspeak lists for Cy1, ecTS, V5dm, and spin systems for hIDD were kindly provided by Drs. Dominque Frueh (Johns Hopkins University), Andrew Lee (University of North Carolina at Chapel Hill), Tatyana Igumenova (Texas A&M University) and Martin Blackledge (Institut de Biologie Structurale), respectively.

**Table 1:** Proteins employed to evaluate BARASA

Protein	MW (kDa)	Sequence length	Data source	Reference assignments (%)^a^
IL-1β^b^	17.5	154	This work	95.9
IL-1Ra^c^ (C66A,C122A)	17.1	152	This work	92.4
IGPS(R43S)^d^	28.4	248	This work	88.0
MBP^e^	40.8	371	This work	95.1
6xHis -CY1^f^	51.9	453	Frueh lab correspondence	90.0
ecTS^g^	61.0	264	Lee lab correspondence	91.2
V5dm^h^	7.7	68	Igumenova Lab correspondence	96.7
hIDD^i^	12.8	110	Blackledge Lab correspondence	70.4

^a^Percentage of non-proline residues assigned a spin system in the reference assignments.

^b^Reference assignments^14^: BMRB 434. Four previously assigned amide groups did not yield cross peaks in the acquired spectra here and were removed from the reference assignments (See Supplementary Table 3). Reference structure: PDB 9ILB.

^c^Deposited to BMRB under accession code 51352. Reference structure: PDB 2IRT.

^d^Deposited to BMRB under accession code 51347. Reference Structure: PDB 1IGS. PDB file was modified using the PYMOL mutation wizard to reflect the R43S substitution.

^e^Reference assignments^54^: BMRB 4354. Ten additional amide group assignments were determined by BARASA and added to the reference assignment set (see Supplementary Table 6). Reference structure: PDB 1DMB.

^f^Reference assignments were provided via Frueh lab correspondence. Reference structure: PDB 7RY6.

^g^Molecular weight corresponds to the symmetric homodimer which is present under experimental conditions. Reference assignments^55^: BMRB 19082. Ten additional amide group resonances were determined by BARASA and added to the reference assignments (see Supplementary Table 8). Reference structure: PDB 1AOB.

^h^Reference assignments^56^: BMRB 18927. One additional amide group assignment was determined by BARASA and added to the reference assignment set (see Supplementary Table 9). No reference structure was used as it is an intrinsically disordered protein.

^i^Cross peak lists were derived from experimentally-determined spin systems by adding simulated noise (see Methods). Reference assignments^57^: BMRB 28135. BARASA extended the assignments considerably but these additional assignments, in contrast to MBP, ecTS, and V5dm, were not included in the reference assignments to avoid misinterpretation (Fig. 3, Supplementary Table 10). No reference structure was used as it is an intrinsically disordered protein.

The results from BARASA were compared to reference assignments to assess program performance. Reference assignments were obtained from either the BMRB, directly from another lab, or manually determined by us (Table 1). Deposited assignments were manually mapped to the acquired spectra for comparison. A small movement in crosspeak positions between the deposited assignments and the acquired spectra was permitted to account for differences in experimental conditions. In addition, a small number of resonances assigned in the deposited data sets were not present in the acquired spectra of IL-1*β*. These were removed from the reference assignments and considered unassigned when assessing algorithm performance (Supplementary Table 3). For the most part, reference assignments were considered complete though in a few cases BARASA identified a small number of additional assignments that were confirmed manually and included in the reference assignments (Supplementary Tables 6–9). For each residue position, BARASA either outputs the spin system and its associated resonances that were assigned to that residue position or marks it as unassigned. The assignment given to each residue in the protein sequence by BARASA was determined to either be matching, missing, or mismatching its counterpart in the reference assignments. A residue was considered to have a matching assignment if the amide group assigned to it by the algorithm was the same as the reference. A residue was also considered to match the reference if it was unassigned both by BARASA and in the reference assignments. A residue was designated missing if an amide group was assigned to that location in the reference assignments, but BARASA did not assign that residue position. Lastly, a residue was labeled as mismatching if BARASA assigned an amide group and it did not match that in the reference assignments or if the residue was unassigned in the reference assignments.

**Fig. 3:**
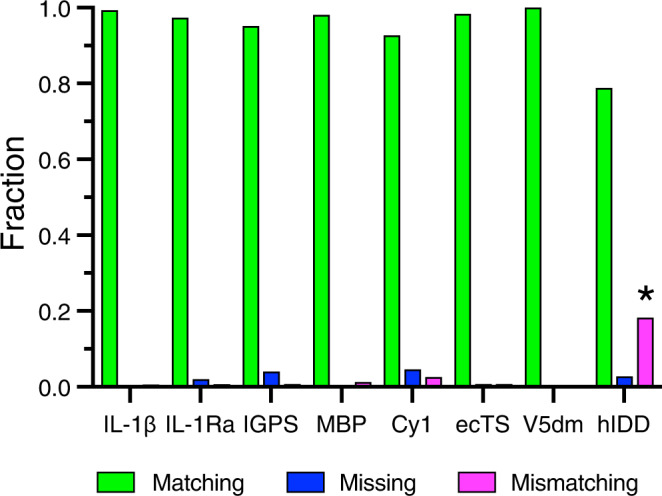
Performance of BARASA using a comprehensive set of triple resonance experiments. Comparison of automated assignment algorithms. Results of automated resonance assignments by BARASA utilizing raw crosspeak lists drawn from a relatively comprehensive set of triple resonance experiments. Compared to manually curated resonance assignments obtained for eight test proteins: interleukin-1*β* (IL-1*β*), interleukin-1 receptor antagonist (C66A, C122A) (IL-1Ra); indole-3-glycerol phosphate synthase (R43S) (IGPS), maltose binding protein (MBP), non-ribosomal peptide synthetase (Cy1), thymidylate synthase (ecTS), V5 domain of protein kinase C (V5dm), and intrinsically disordered region of human ANP32A (hIDD). Shown are the fractions of residues that are accurately matched (green), mismatched (magenta), or missing (i.e., unassigned) (blue) to the reference assignments. *In the case of hIDD, a number of de novo assignments were indicated by BARASA and are included as mismatching with the reference assignments. See main text and Table 1. Source data are provided in the Source Data file.

In general, BARASA’s performance when utilizing structure-based chemical shifts and crosspeak lists derived from a comprehensive set of triple resonance experiments is marked by (nearly) complete assignments when compared to the manually curated reference assignments and, most importantly, produced very few errors (Fig. 3 & Supplementary Table 2). Individual statistics for each assignment are listed in Supplementary Tables 3–10. BARASA had relatively more difficulty with the Cy1 and IGPS examples. This is likely due to a higher degree of variance in resonance chemical shifts of the backbone spins among the different spectra relative to the test cases because of the employment of multiple independently prepared samples, but the performance overall remained very good (Fig. 3). In the case of hIDD, a relatively high apparent mismatch rate is observed. Upon closer examination, the mismatching assignments made by BARASA were all assignments not previously reported as assigned. Many of these previously unreported assignments fall within regions of the sequence with low complexity (Supplementary Table 10) which is likely why they were difficult to assign manually. While there are no independent data supporting their veracity, these assignments proposed by BARASA and, as we discuss more below, by the next best performing automated assignment algorithm FLYA^34^ are highly similar and are likely to be largely correct.

BARASA utilized SHIFTX+^35^ predicted chemical shifts for the globular test proteins, whereas the algorithm utilized random coil chemical shifts^36,37^ for the so-called IDP examples as predicted shift restraints during annealing (see Methods). SHIFTX+ was chosen as it appears to be among the best-reported chemical shift prediction algorithm based solely on three-dimensional structural information and other physical parameters (i.e., temperature, pH). The related algorithm SHIFTX2, though it gives more accurate predictions, relies on the analysis of shifts from homologous proteins as well as the three-dimensional structural inputs specific to the protein being analyzed. It was our concern that the accuracy of SHIFTX2 would vary with the number of homologs available and, under circumstances of sparse homologs, result in significantly larger errors than are reported for the average case. As accurate estimation of prediction error is crucial to the Bayesian analysis (Methods), inaccurate and/or unaccounted for variance in prediction errors could compromise performance. Furthermore, as SHIFTX2 performs searches for the known chemical shifts of homologous sequences as part of its prediction, it would utilize the previously assigned shifts of our test proteins to present the BMRB in the generation of the predicted shifts. Such shifts would not be generally available for the de novo assignment of a protein and would thus be an invalid test of BARASA. We also note that using predicted chemical shifts generated by SPARTA+^38^ gave similar results (Supplementary Table 11) as when using those predicted by SHIFTX+.

In this regard, it is important to appreciate that it is statistically anticipated from the distributions of chemical shifts, either predicted or documented in the BMRB, that values outside the error range will be encountered. For example, if the distribution were taken as Gaussian and employing the standard deviation as the prediction error (see Methods), approximately 32% of all predictions would be expected to be outside of the considered error range. This is what is observed. Supplementary Tables 3–10 contain the likelihoods of the spin systems for the various test proteins. These likelihoods represent the probability of observing the experimental shifts given that the assignment is correct and ranges from 0 to 1. Likelihoods lower than 0.32 correspond to spin systems with predicted resonance chemical shifts that are, on average, beyond the specified error range but are nevertheless well accommodated by BARASA.

Finally, BARASA also produces a curated set of assignments from 20 annealing runs within 1 hour for each system tested (see Supplementary Table 12). With high accuracy and runtimes under an hour, the advantages of BARASA become even more apparent when considering large proteins with suboptimal data sets.

### The performance of BARASA with suboptimal data sets

The rather complete crosspeak lists from an extensive set of triple resonance experiments for each test protein provide valuable benchmarks for the validation of BARASA, but are arguably not fully illustrative of the difficult protein systems often challenging current applications of protein NMR spectroscopy. To examine the performance of BARASA in cases of missing data and to illuminate the most impactful triple resonance information, individual crosspeaks or all crosspeaks of entire spin systems from the MBP and ecTS data sets were randomly discarded to generate compromised data sets, emulating data collection on challenging protein systems. Individual crosspeaks were randomly retained in the data set with a probability based on the crosspeak type (i.e., Cα, Cβ, or CO resonance). This process was done over a wide range of retention probabilities to produce a multitude of distinct data sets that represent a wide range of data completeness. These depleted peaks lists were then used as input to BARASA the results of which are provided in Supplementary Tables 13 and 14. In this way, the relative importance and completeness of different types of spectral data as well as the effects of entirely missing spin systems could be probed. In addition, a key question was to learn the extent to which structure-based chemical shifts, as opposed to general BMRB residue-specific statistics, can rescue the assignment and aid the assignment process.

**Fig. 4:**
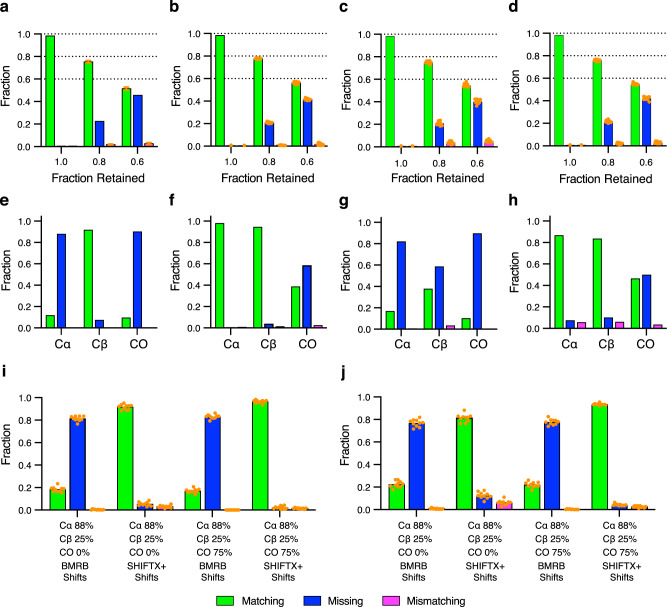
Performance of BARASA under data sparse conditions. Shown are the fractions of residues that are accurately matched (green), mismatched (magenta) or missing (i.e., unassigned) (blue) to the reference assignments. Panels **a**–**d** correspond to results from input data sets where entire spin systems were discarded from the crosspeak lists. The ordinate axis is the fraction of retained spin systems and the dashed lines indicate the maximum fraction of possible matching assignments. The effects of random spin system depletion on the analysis of MBP both randomly (**a**) and as stretches of five consecutive residues being discarded (**b**). A similar analysis of ecTS with either individual (**c**) or groups of five consecutive spin systems being discarded (**d**). For the conditions 0.8 and 0.6 fractions retained, ten random data sets retaining the indicated fraction of spin systems were generated. The performance of BARASA on each data set is shown as a single orange solid circle, with the bar height representing the arithmetic mean. The full data set (“1.0” condition) results were taken from Fig. 3. Only one result with the full data set was measured to avoid the comparison of run-to-run variation with variation due to differences in the input data set. The effects of restricting connectivity information by utilizing only a single pair of triple resonance experiments with either residue-type statistics (BMRB) (**e**) or structure-based (SHIFTX + ) (**f**) chemical shift predictions for MBP. Similarly, for ecTS using only residue-type statistics (BMRB) (**g**) or structure-based (SHIFTX + ) (**h**) chemical shift predictions. The effect of random depletion of crosspeaks from the comprehensive set of triple resonance experiments where the indicated percentages each type of crosspeak that are retained is illustrated for the MBP (**i**) and ecTS (**j**) data sets and used with residue-type statistics (BMRB) or structure-based (SHIFTX+) predicted chemical shifts. Results of ten individual runs (*n* = 10) are plotted as solid orange circles and bar heights represent the arithmetic mean. Source data are provided as a Source Data file.

Figure 4 illustrates the robustness of BARASA when analyzing conditions of missing spectral data. This specific example was generated using retention probabilities of 88% and 25% for the Cα- and Cβ-based information, respectively, and with retention probabilities of either 0% or 75% for the CO-based information. Reliance on the BMRB database for predicted shifts, as opposed to structure-based shifts, yielded poor performance. In brief, the use of structure-based SHIFTX+^35^ predictions entirely rescues the resonance assignment. These data indicate that the availability of the structure-based chemical shift predictions serves as a powerful restraint in protein assignment - large enough to potentially surpass the information provided by the CO experimental pair under many circumstances. This is likely due to the fact that spin system adjacency is established adequately with the Cα and Cβ spectral information and the remaining assignment ambiguity is due to residue type matching; CO resonances provide little residue type information and offer little help in this respect. We do not believe this observation to be an artifact of the parameterization of the energy function since carbonyl-derived connectivities are weighted roughly the same as the chemical shift probability (Methods). As such, the energy provided by CO connectivity information would be of a similar magnitude of the total chemical shift energy of the spin system.

Randomly retained spin system data sets were generated in two ways: by allowing all crosspeaks of any spin system assigned in the reference assignments to be randomly discarded from the input data set until only the indicated fraction of the assigned spin systems remained or by discarding the crosspeaks of random spin systems in the same manner, with the added condition that only those from sets of five random, but contiguous in sequence, spin systems are discarded. The latter condition was performed to simulate the performance of BARASA under the common situation where exchange broadening arising from physical motion of contiguous stretches of sequence (e.g., loops) results in loss of amide resonances. In both cases, BARASA is still able to produce the overwhelming majority of the possible assignments without errors even when up to 40% of the spin systems are missing (Fig. 4). There is little difference in performance whether the missing data is localized or distributed across the sequence. The performance of BARASA when challenged with artifact peaks, which often arises from low-concentration or unstable samples or instrumentation, was also examined. In this case, a depleted data set from above was augmented with randomly generated artifact peaks. Only a modest decrease in performance is observed even when the crosspeak list is contaminated with 20% artifactual entries (Supplementary Fig. 1).

Even with the considerable time-savings introduced by non-uniform sampling^39^, collection of NMR data on proteins is still time intensive. The superior performance of BARASA on missing data within a comprehensive set of triple resonance experiments raised the possibility that BARASA could tolerate a reduced set of triple resonance experiments. We tested this hypothesis using ecTS and MBP where information from a single triple resonance experimental pair (e.g., HNCA and HN(CO)CA) combined with BMRB or SHIFTX + predicted shifts were analyzed. The Cα- and Cβ-type triple resonance pairs are equally useful in the BARASA assignment process when provided SHIFTX+ shifts, but the Cβ information becomes relatively more effective when relying on BMRB amino acid distributions (Fig. 4). This is clearly due to the higher residue type information intrinsic to the Cβ resonance. Overall, BARASA performs extremely well with either the Cα -or Cβ-type triple resonance experimental pairs only. In contrast, the CO-type triple resonance experimental pair when used alone is much less effective, likely due to the reduced sensitivity of carbonyl carbon shifts to amino acid type and local structure.

### Comparison to alternate automated resonance assignment algorithms

**Fig. 5:**
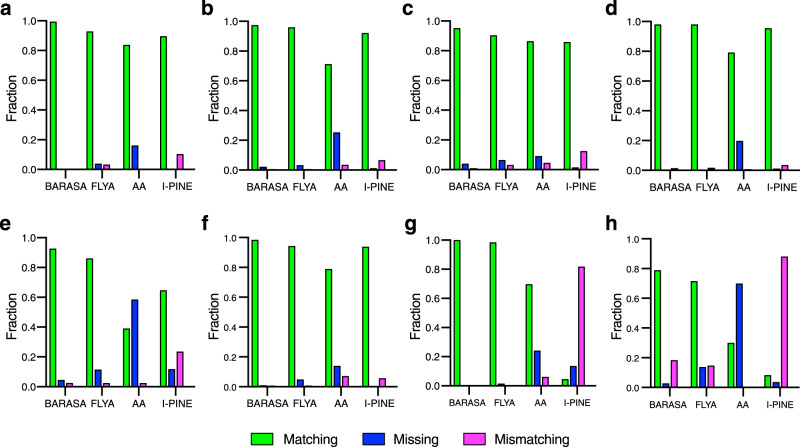
Comparison of BARASA against other assignment algorithms. Performance of BARASA, FLYA, AutoAssign (AA), and I-PINE against reference triple resonance assignments of six protein systems: **a** IL-1*β*; **b** IL-1Ra; **c** IGPS; **d** MBP; **e** CY1; **f** ecTS; **g** V5dm; **h** hIDD. Shown are the fractions of residues that are accurately matched to the reference assignments (green), incorrectly matched (magenta) or missing (i.e., unassigned) (blue). *BARASA and, to a lesser extent, FLYA extended the reference assignments for hIDD considerably (Supplementary Table 10). The extended assignments are therefore denoted here as mismatching. Source data are provided as a Source Data file.

Computer-assisted resonance assignment strategies for analysis of triple resonance spectra have been employed for over two decades. For the sake of comparison, three highly-cited algorithms were compared to BARASA: FLYA, AutoAssign^22^, and I-PINE^40^. The same crosspeak lists derived from the comprehensive set of triple resonance experiments were used for all four algorithms (Fig. 5). BARASA achieved the highest percent matching among all the algorithms against the reference assignments in all test cases. BARASA outperformed AutoAssign and I-PINE by considerable margins, most notably with the two IDPs examined, while offering only marginal improvement over FLYA (Supplementary Table 2). Importantly, BARASA made few mismatching assignments (<3%) while I-PINE had up to 20% mismatches meaning that about 1 in 5 assignments made were incorrect. For these reasons, AutoAssign and I-PINE were not examined further.

**Fig. 6:**
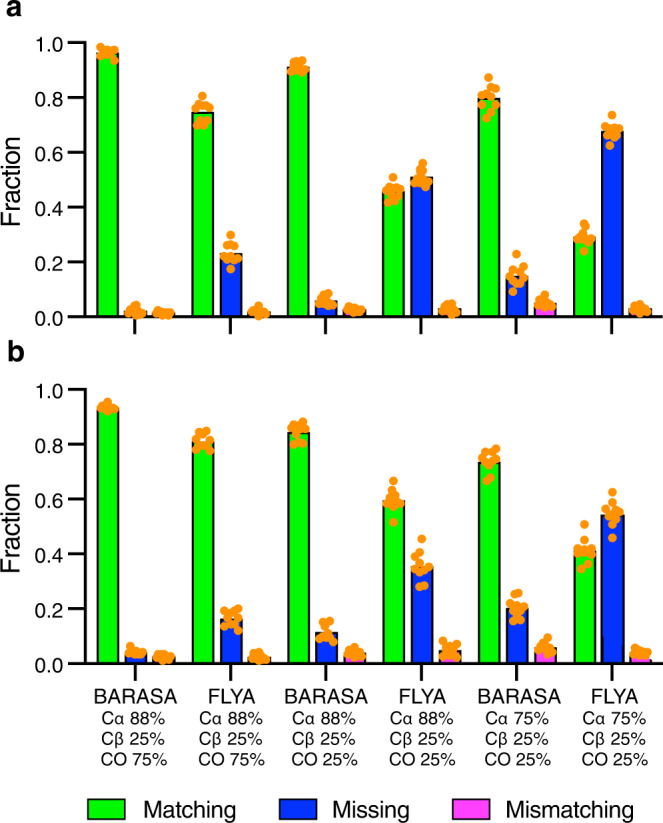
Performance of BARASA and FLYA with depleted crosspeak lists. The effects of random crosspeak depletion on the analysis of MBP (**a**) and ecTS (**b**) comprehensive triple resonance data sets with partial retention of the indicated crosspeak types (see text and Fig. 4). Shown are the fractions of residues that are accurately matched (green), mismatched (magenta) or missing (i.e., unassigned) (blue) to the reference assignments. Ten independent data sets (*n* = 10) were randomly generated for each depletion condition. The results of analysis by BARASA for each data set are shown as solid orange circles and the bar heights correspond to the mean. Source data are provided as a Source Data file.

The marginal advantage of BARASA over FLYA when utilizing a comprehensive triple resonance data set prompted us to examine their behavior in the more challenging situations commonly encountered. BARASA’s performance in settings where there is a significant amount of missing data was compared against FLYA. MBP and ecTS crosspeak lists with varying retention probabilities were generated and used as input for BARASA and FLYA (Fig. 6). BARASA was able to generate a higher assignment match rate in all scenarios with the difference in performance between the algorithms growing as the data became increasingly sparse. In addition, the mismatch rate between the algorithms remained similar. These results demonstrate that BARASA has excellent outcomes in circumstances where there is a large quantity of missing data – greatly outperforming existing algorithms.

### Use of predicted versus experimentally determined structural models

It is clear from Fig. 4 that use of structure-based chemical shift predications provides significant advantages over simple residue-type predictions derived from empirical distributions. This is particularly true in the case of Cy1, which is perhaps an exemplar of the challenges facing modern protein NMR and required a battery of experimental spectra and labeling schemes^41^. The sheer number of samples and experiments required resulted in a relatively high variation in resonance positions among the spectra. The resonance assignment was carried out in the absence of an experimentally determined structural model with the closest homolog having only 38% identity. Accordingly, the resonance assignment of Cy1 must be considered a significant achievement.

**Fig. 7:**
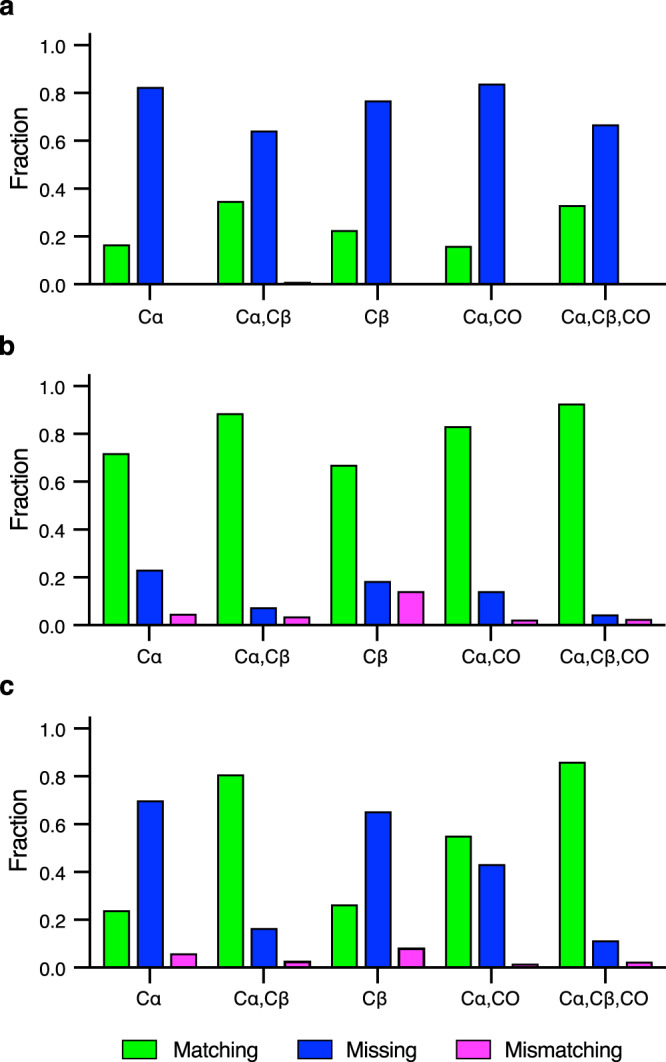
Sensitivity of automated resonance assignment of Cy1 to breadth of triple resonance experimental foundation and chemical shift prediction precision. The resonance assignment by BARASA using the indicated cross crosspeak types from the triple resonance spectra and, residue-type (BMRB) chemical shift statistics (**a**) or chemical shifts predicted by SHIFTX + based on a structural model provided by AlphaFold2 (**b**). Triple resonance data sets include the peaks from the following spectra: HNCA/HN(CO)CA (Cα), HN(CA)CB/HN(COCA)CB (Cβ) and HNCO/HN(CA)CO (CO). Bar heights indicate the fractions of residues that are accurately matched (green), mismatched (magenta) or missing (i.e., unassigned) (blue) to the reference assignments. Equivalent runs with FLYA (**c**) using the data set of (**b**) reinforce the conclusion that BARASA is more robust to non-ideal data. Source data are provided as a Source Data file.

The absence of an experimentally-determined atomic-resolution structure of the protein of interest is a common occurrence and can severely limit the resonance assignment process. However, powerful structure-prediction algorithms have recently been introduced^42^ and we sought to learn how the availability of structures predicted by the AlphaFold2 algorithm influence the performance of BARASA. Chemical shifts predicted by SHIFTX+ using the structure of Cy1 predicted by AlphaFold2 were used for analysis by BARASA. Using only residue-type information based on the BMRB resulted in poor performance. However, when utilizing the predicted chemical shifts from the predicted structure of Cy1, BARASA recapitulated its performance based on the NMR-derived structure and a comprehensive set of triple resonance experiments. In addition, BARASA performed very well using subsets of triple resonance experiment pairs and significantly outperformed FLYA (Fig. 7). This level of success of BARASA using SHIFTX+ in concert with structures predicted by AlphaFold2 was observed across the test data sets (Supplementary Table 15). Taken together these data suggest that the lack of an experimental structure is unlikely to hinder the full capability of the BARASA algorithm.

In summary, we have demonstrated that Bayesian-based simulated annealing combining sequential relationships derived from triple resonance spectra and chemical shift information predicted from a high-resolution structural model can greatly facilitate the triple-resonance backbone assignment of proteins. The implementation of this strategy in BARASA is robust to incompleteness of spin system definition (sparseness) and overall complexity of the resonance assignment challenge (protein size). Importantly, BARASA is relatively conservative and makes few errors. An optimized annealing strategy utilizing a specific heat approach to guide temperature cooling results in a very rapid analysis. The speed of analysis combined with its aforementioned robustness clearly positions BARASA to inform on the real time data acquisition side of the resonance assignment process. This becomes increasingly feasible with the utilization of automated crosspeak picking. Iterative examination by BARASA of sequentially acquired triple resonance spectra could, in principle, allow the user to determine if a satisfactory level of assignment can be achieved without further data acquisition and thereby save valuable spectrometer time. In summary, the BARASA algorithm provides the ability to easily and robustly assign unusually difficult protein systems and simplify this otherwise challenging task. The combination of fast and robust backbone resonance assignments with structure-based methyl resonance assignments^43–48^ will reduce the resonance assignment barrier considerably and allow greater application of the power of NMR spectroscopy to be applied in a facile manner to otherwise challenging proteins.

## Methods

### NMR sample production

A vector encoding the gene for Interleukin 1- β (IL-1β) was transformed into E. coli BL21DE3 cells and expressed in 1 L of 95% D_2_O M9 media containing ^15^NH_4_Cl and ^2^H,^13^C glucose as the sole nitrogen and carbon sources, respectively. Cells were grown at 37 °C to an OD_600_ of 0.9 and induced with 1 mM IPTG. Induction continued for 4 hrs at 37 °C until harvesting via centrifugation at 3500xg and frozen overnight. The cell pellet was then thawed, resuspended in 10 mM potassium phosphate pH 8.0, 0.2 mM EDTA, 5 mM DTT and 1 mM PMSF. The cells were then lysed by sonication and centrifuged at 32,000xg for 30 min at 4 °C. Lysate was then brought to 80% saturation with NH_4_SO_4_ and allowed to stir for 1 hr at 4 °C. The suspension was then centrifuged for 30 min at 32,000 x *g* 4 °C and the pellet was resuspended in 25 mM ammonium acetate pH 4.5, 1 mM BME and dialyzed overnight (8 kDa MWCO) in the same buffer at 4 °C. The dialyzed protein was then loaded onto a HiTrap Capto S column (Cytiva Life Sciences) equilibrated in 25 mM ammonium acetate pH 4.5, 1 mM BME and eluted with a linear gradient up to 500 mM ammonium acetate pH 4.5, 1 mM BME. Protein was then frozen and lyophilized. The lyophilized protein was dissolved in 20 mM Tris pH 8.0, 7 M urea, 20 mM DTT and added drop wise to 20x volume of 20 mM tris, 100 mM NaCl, 5 mM DTT pH 8.0 under constant stirring. The refolded protein was then dialyzed against 50 mM sodium acetate pH 5.0, 5 mM DTT and concentrated to 0.67 mM. To this sample 0.02% NaN_3_, 100 μM DSS and 5% D_2_O was added. Triple resonance spectra were acquired at 23 °C on an 800 MHz (^1^H) Bruker NEO spectrometer running TopSpin and equipped with a CryoProbe.

A vector encoding the gene human interleukin-1 receptor antagonist (IL-1Ra) containing C66A/C122A amino acid substitutions was expressed using *E. coli* BL21(DE3) cells in M9 minimal media. The M9 minimal media contained ^15^NH_4_Cl and ^13^C-glucose as the sole nitrogen and carbon sources respectively. The culture was centrifuged at 5000 rpm, and the cell pellet was resuspended in 20 mM Tris, 500 mM NaCl, 20 mM imidazole, pH 7.9 for sonication. Sonicated cells were centrifuged at 15000 rpm, and supernatant was loaded onto a His60 column (Takara Bio USA). The column was washed with 20 mM Tris, 500 mM NaCl, 40 mM imidazole, pH 7.9; and protein was eluted with 20 mM Tris, 500 mM NaCl, 500 mM imidazole, pH 7.9. The collected protein fraction was buffer exchanged to 12.5 mM HEPES, 50 mM NaCl, 5 mM CaCl2, pH 6.5 for His-tag removal by FXa protease (New England Biolabs) and further purified both by affinity (His60 resin) and size exclusion chromatography (S-75 Sephadex, Cytiva Life Sciences). The NMR sample was prepared by buffer exchanging the protein into 100 mM NaCl, 25 mM MES, pH 6.0 and concentrated to 1 mM, with the addition of 100 μM DSS, 5% D2O, and 0.02% NaN3 (Supplementary Table 1). Triple resonance assignment experiments were acquired at 35 °C on either a 500 MHz Bruker Avance spectrometer or an 800 MHZ (^1^H) Bruker NEO spectrometer both equipped with a Cryoprobe.

A R43S variant of the gene for indole-3-glycerol phosphate synthase from *S. solfataricus* (IGPS) was cloned in a modified pGS-21a vector downstream of an N-terminal His-tag and TEV protease site. This expression plasmid was a gift from the lab of Professor Robert Matthews, University of Massachusetts Medical School, Worcester. IGPS R43S protein was expressed in BL21(DE3) competent cells with ampicillin antibiotic selection. Cells were grown at 37 °C until they reached an OD600nm of 0.6 and 1 mM IPTG was added to induce expression for 16-20 h at 25 °C. To isotopically label the protein for NMR spectroscopy, cells were grown in M9 minimal medium with ^15^NH_4_Cl and ^13^C-glucose as the nitrogen and carbon sources, respectively. Cells were lysed in 100 mM potassium phosphate, pH 7.5, 50 mM KCl, 5 mM imidazole by sonication. The lysate was loaded onto a Ni^2+^-NTA column pre-equilibrated with the lysis buffer. Impurities weakly bound to the column were washed away with 100 mM potassium phosphate, pH 7.5, 150 mM KCl, 75 mM imidazole, followed by equilibration into the low salt buffer 100 mM potassium phosphate, pH 7.5, 50 mM KCl, 75 mM imidazole. Protein was eluted with 100 mM potassium phosphate, pH 7.5, 50 mM KCl, 500 mM imidazole and dialyzed into lysis buffer. Purified His-tagged protein was concentrated to 5 mL, and tag was cleaved with TEV protease added at 1:30 mass ratio and mixing at RT overnight. Untagged protein was separated TEV protease and uncleaved protein by Ni^2+^-affinity chromatography. Protein aliquots were flash frozen and stored at −80 °C. NMR samples of ^15^N^13^C-labeled IGPS were prepared at 250 µM concentration in 60 mM potassium phosphate, pH 7.2, 50 mM KCl, 5% D_2_O, 100 μM DSS. All data were collected on a 750 MHz (^1^H) Bruker AVANCE III NMR spectrometer equipped with a CryoProbe at 50 °C.

A vector encoding maltose binding protein (MBP) was transformed into BL21DE3 cells and expressed in 1 L of 95% D_2_O M9 media containing ^15^NH_4_Cl and ^2^H,^13^C glucose as the sole nitrogen and carbon sources respectively. Cells were grown at 37 °C to an OD_600_ of 0.9 and induced with 1 mM IPTG. Induction continued for 4 hrs at 37 °C until harvesting via centrifugation at 3500 × g. The cell pellet was frozen overnight and resuspended in 20 mM Tris-HCl, 20 mM NaCl pH 8.0, 1 mM DTT. 6 mg of Lysozyme was added and was incubated under stirring for 30 min at room temperature. Cells were further lysed by sonication and centrifuged at 32000 × g for 30 min at 4 °C. Clarified lysate was filtered (0.45 um pore size) and loaded onto a 25 ml DEAE column equilibrated in 20 mM Tris, 20 mM NaCl, pH 8.0, 1 mM DTT. The protein was eluted using a gradient to 20 mM Tris, 500 mM NaCl. Protein was concentrated to 1-2 ml and run on a 112 ml Superdex 75 column equilibrated in 20 mM Tris, 20 mM NaCl, 2 mM DTT pH 8.0. The protein was pooled and unfolded by dialysis in 4 M GuCHl, 20 mM Tris-HCl, 1 mM DTT pH 7.5. Protein was refolded by repeated 10x dilution with 20 mM sodium phosphate pH 7.1, 1 mM EDTA, 2 mM β-cyclodextrin, 0.02% NaN3, 100 μM DSS 5%D_2_O followed by concentration (4 times). From this a 0.5 mM sample of MBP was created. Spectra were acquired at 37 °C on an 800 MHz (^1^H) Bruker NEO NMR spectrometer. NMR data acquisition and processing parameters recorded by us for IL-1β, IL-1Ra, IGPS and MBP are summarized in Supplementary Table 1. Poisson gap NUS spectra were reconstructed using hmsIST^39^ and all spectra were processed with NMRpipe^49^ on NMRBox^50^. Spin systems were built by manual peaking picking using NMRFAM-SPARKY^51^ and referenced using DSS.

### Origin of protein test data sets

Triple resonance data acquired in our laboratory were processed using the NMRPipe^49^ installed on NMRbox^50^. The crosspeak lists were constructed from data acquired in our laboratory (see Table 1) by manually crosspeak picking using NMRFAM-SPARKY^51^ (i.e., not reconstructed from deposited assignments) (see Table 1). Crosspeak lists for ecTS, Cy1 and V5dm were provided by Professors Andrew Lee (University of North Carolina, Chapel Hill), Dominique Frueh (Johns Hopkins University), and Tatyana Igumenova (Texas A&M University), respectively, and were used without further adjustment. Crosspeaks for hIDD were generated from spin systems provided by Professor Martin Blackledge (Institut de Biologie Structurale) in the following manner. Each provided spin system consisted of an amide proton (H) and amide nitrogen (N) chemical shift as well as chemical shifts for Cα, Cα(i-1), CO and CO(i-1) resonances (though a complete set of carbon resonances were not present for each spin system). HNCA, HN(CO)CA, HNCO, and HN(CA)CO crosspeak lists were generated from the spin system data by adding the following crosspeaks to the indicated crosspeak list from each spin system: H-N-Cα(i-1), H-N-Cα for the HNCA; H-N-CO and H-N-CO(i-1) for the HN(CA)CO; H-N-CO for the HNCO and H-N-Cα(i-1) for the HN(CO)CA. The resonance values for the crosspeak positions were drawn from a normal distribution with a mean given by the value of the resonance in the spin system and a standard deviation of 0.003, 0.04, and 0.04 ppm for hydrogen, nitrogen and carbon resonances, respectively.

### BARASA algorithm description

The algorithm begins by reading in the crosspeak lists to assemble spin systems. Within the crosspeak lists, the user provides the possible crosspeak types that are produced by the experiment. For example, the HNCA would produce possible crosspeak types of H-N-CA(i) and H-N-CA(i-1). The user also specifies cutoff values for each spectral dimension that dictate the range over which chemical shifts will be matched during spin system construction. The provided crosspeak types dictate which dimensions have resonances of ambiguous type. In the example of the HNCA, the first two dimensions are of unambiguous type (H and N resonances respectively). However, the third dimension is ambiguous (CA(i) or CA(i-1)).

BARASA builds crosspeak lists by first arbitrarily choosing a crosspeak to seed the construction of the spin system. All other crosspeaks are searched to find those that have at least two resonances of unambiguous type that match the resonances of the seed crosspeak, both in terms of their chemical shift (i.e., fall within a tolerance cutoff specified by the user) and resonance type. After each subsequent addition, BARASA attempts to resolve ambiguous resonance types based on known chemical shifts already present in the spin system. For example, if a spin system has a Cα(i-1) value of 56.0 ppm (with a tolerance of 0.3 ppm) and a HNCA crosspeak, which is added (which could have a resonance type of Cα or Cα(i-1)) with a value of 58.0 ppm, then the algorithm will resolve the type of the new crosspeak as the Cα as it is not within the 0.3 ppm tolerance of the 56.0 ppm Cα(i-1). After adding the crosspeak and resolving type, the algorithm then iterates through the entire list of remaining crosspeaks and repeats the above addition procedure. Once no more peaks can be added to the spin system, a new crosspeak is arbitrarily chosen from the list of remaining peaks to seed the construction of additional spin systems. This continues until all peaks have been added to a spin system.

If BARASA finds a crosspeak that has two unambiguous resonances that match those already present in a spin system, but contains additional resonances that have shifts which conflict with those that are already present in the spin system, then an additional spin system in which to place the incongruent crosspeak is created. Such as situation arises due to spectral degeneracy (e.g., two spin systems with the same or similar amide shifts). The algorithm will then attempt to add the remaining peaks to both spin systems. Any further clashes are resolved by the generation of a new spin system. This continues until no more crosspeaks can be added to any spin system. The crosspeaks within this group of spin systems are then marked by the algorithm to be allowed to exchange to any other spin system within the group during the annealing process. In addition, the user has the option to allow the algorithm to use a crosspeak cache to which low intensity peaks (lowest 5%) can be added to over the course of the annealing run to provide a mechanism to eliminate potential artifactual crosspeaks.

Once all the crosspeaks to a spin system have been added, all possible resonance type sets are generated for that spin system. A resonance type set is a complete designation of each atom type of each crosspeak in a spin system. If a spin system only contains peaks with no ambiguous resonance types, then the spin system has only one possible resonance type set. This is the case for the majority of data sets as experiments with ambiguous resonance types are often paired with experiments that resolve this ambiguity (e.g., HNCA, HN(CO)CA experimental pair). However, if ambiguous resonance types are present in a spin system, then the spin system will contain all possible resonance type sets. A distinct set of average resonance values for the spin system are calculated for each resonance type set; all of which will be considered over the course of the annealing run.

The resonance assignment analysis is then initialized by randomly assigning the spin systems to the protein sequence. Often there are more spin systems than are residue positions (e.g., spin systems correlated to a side-chain amide group and not the backbone are also present in the data set). Any spin systems that were not randomly placed on the sequence, are placed in a spin system cache and may be assigned to the sequence over the course of the run. The simulation temperature is initialized at 1000 arbitrary units and a spin system or crosspeak is chosen at random to swap. The probability that a swap will be a crosspeak swap is set at 0.01 (which was found to be a good compromise between sampling and algorithm speed) with the remaining swaps being spin system swaps. A chosen spin system will have the ability to be added to the spin system cache, swap positions with another spin system, or move to an empty position in the sequence, making its former position available. Whenever a spin system is moved, a random resonance type set is chosen from among those possible. In addition, the algorithm may attempt to change the current resonance type set and keep the current spin system in place. If a crosspeak is chosen to swap, it has the potential to be added to the crosspeak cache (if it is of low intensity), added to another spin system within its spin system group, or swap places with any crosspeak within its spin system group. Upon moving/swapping cross peaks, the affected spin systems are evaluated for clashes. If there are none, the crosspeak swap is allowed to continue, otherwise the swap is rejected. In addition, a crosspeak move/swap will trigger the affected spin systems to generate all new resonance type sets and choose one at random from the possibilities. This forces a recalculation of average chemical shifts for each resonance type set of each spin system resonance, as well as the Bayesian probabilities described below for sequence position determination.

If the swap is not immediately rejected due to a crosspeak clash, the change in energy of the system due to the swap is calculated using the energy function described below. The swap is then accepted or rejected at a frequency corresponding to a probability generated by applying the Metropolis criterion (Eq. 1). After each successful swap, the energy of the state is recorded and stored as a part of a sample of energy values. Once the sample reaches a particular size, the sample mean and standard error are calculated and an additional sample is generated by continued swapping. A Student’s two tailed t-test is performed to compare the sample means of the two samples. The system is considered to have equilibrated at the current temperature if the *p*-value of the t-test is greater than a user supplied value (default *p* > 0.5). If equilibration has not been reached, more swaps are performed to generate an additional sample and the t-test is repeated with the two most recent samples. If equilibration has been reached, then the energy values are used to estimate the specific heat at the current temperature:

2 $$d\left\langle E\right\rangle /{dT}=\left(\langle {E}^{2}\rangle -{\left\langle E\right\rangle }^{2}\right)/{T}^{2}$$

Where $T$ is the current temperature in arbitrary units, $E$ is the energy of the system and the angled brackets indicate the sample mean. Large drops in average ensemble energy due to oversized temperature steps can lead to the system becoming trapped in a local minimum. By deciding on a target energy drop that is unlikely to lead to a frustrated state, we can utilize the specific heat calculated at each temperature to estimate the temperature drop needed to achieve the target change in energy. This is done in the following manner:

3 $${T}_{{next}}=T+\triangle {\left\langle E\right\rangle }_{{{\mbox{target}}}}/\left(\frac{d\left\langle E\right\rangle }{{dT}}\right)$$

Where $\triangle {\left\langle E\right\rangle }_{{target}}$ is a user-controlled parameter and is kept at −2000 for this study. Decreasing the magnitude of the target energy drop, in situations where the system is becoming trapped in a frustrated state can lead to better results at the expense of longer simulation time. If ${T-T}_{{next}}$ is greater than 10, then the temperature decrease is limited to 10 units to prevent overcooling the system. The use of the specific heat in this manner results in smaller temperature steps at temperatures where the system is rapidly decreasing in energy, while allowing for larger steps when drops in temperature have a modest effect on the ensemble. The resulting schedule avoids system quenching while simultaneously minimizing unproductive swaps at temperatures that are either too high or too low for effective annealing. After decreasing the temperature, the annealing run will terminate if any of the following criteria are met: the temperature is less than 1, the product of the temperature and the last specific heat calculated is less than 200, or the ratio of unsuccessful swaps to successful swaps while collecting the last sample is greater than 10,000. The rational for the criteria are as follows: Given the standard energy parameterization, productive annealing is unlikely to happen at temperatures below 1; the product of specific heat and current temperature (at low temperatures) provides a crude estimate as to the amount of energy between the current ensemble and global minimum (i.e. the thermodynamic ensemble at T = 0, which should correspond to a single state) and approximately 200 energy units is negligible; and at this ratio of unsuccessful to successful swaps, the system is near a minimum and further sampling is inefficient. If termination is not achieved, a new sample size is defined using the following equation:

4 $$S=\,{{{{{\rm{min}}}}}} \left({{{{{\rm{max}}}}}} \left(N\frac{{\left(d\left\langle E\right\rangle /{dT}\right)}^{1.5}}{300},\,10000\right),\,100000\right)$$

Where *N* is the number of residues in the sequence. This equation permits increases in sample size when sampling at temperatures with high specific heats, which is where the most productive swaps occur. This approach also permits scaling of sampling for larger proteins. The parameters of this equation were found empirically to be a good compromise between sufficient sampling and speed. Samples are then drawn at the new temperature to determine equilibration and the cycle is continued. Upon termination of the annealing protocol, a steepest-descent type search is performed to locally minimize the system energy and refine the assignments, discarding potentially bad assignments that were left over from the run. This is done by attempting to place (or swap) every spin system/peak at every possible location in the sequence/spin system group (including the cache, if allowed). Only spin system/peak swaps/placements that decrease the system energy are accepted. This is repeated 100 times.

This entire process of simulated annealing is independently repeated with a number of different random starting conditions. Here we have used 20. A consensus set of assignments is generated by calculating the frequency with which each spin system is placed at each amino acid location. The spin system assigned to each residue location in a majority of the runs (if any) is kept as the consensus spin system. A curated set of assignments is generated from this consensus analysis. The curation procedure is as follows: the consensus spin system at each residue was chosen as the tentative assignment for that particular residue. Residues without a consensus spin system (i.e. did not have the same spin system assigned to it greater than 50% of the time) were marked as unassigned. Tentatively assigned spin systems are then evaluated by the posterior probabilities as well as the number of connectivities defined as a matching resonance between adjacent spin systems. Assignments were accepted if they met any of the following criteria: 1) the assigned spin system has at least two connectivities with adjacent spin systems, 2) the assigned spin system has at least 1 connectivity with adjacent spin systems and a posterior probability at least three times higher than the quantity 1/*N*, or 3) the assigned spin system has a posterior probability > 50%. Residues with tentative assignments that did not satisfy any of these criteria were then marked as unassigned.

The energy function used in the annealing routine is calculated as the sum of all the energies of the constituent spin systems (*E*_*tot*_) (Eq. 5). At any given step during the annealing protocol, spin systems are either tentatively assigned to a position in the sequence or placed in the cache. Cached spin systems are defined as having zero energy (i.e., ${E}_{m}=0$).

5 $${E}_{{tot}}=\mathop{\sum }\limits_{m=0}^{M}{E}_{m}$$

The energy of each spin system tentatively assigned to a specific place in the amino acid sequence is comprised of the adjacency energy (${E}_{m}^{{adj}}$) and the chemical shift energy (${E}_{m}^{{cs}}$):

6 $${E}_{m}=\,{E}_{m}^{{adj}}+\,{E}_{m}^{{cs}}$$

The adjacency energy is related to the degree of correspondence between the averages of the Cα, Cβ and CO resonances of the current spin system and the averages of the Cα (i-1), Cβ (i-1) and CO (i-1) resonances of the spin system tentatively assigned to the subsequent position in the sequence. Each average resonance value in a spin system is calculated as the arithmetic mean of all resonance chemical shifts of the indicated type from all of the crosspeaks that contain that resonance currently in the spin system. ${E}_{m}^{{adj}}$therefore, captures the process of evaluating spin system adjacency and is based on the number of potential connectivities between adjacent spin systems tentatively assigned to the sequence. For example, if spin system *m* is assigned to a residue position immediately prior to that of spin system *l*, then the adjacency energy is given by:

7 $${E}^{{adj}}=\,{\sum }_{k}\left[{c}_{0}\exp \left({\frac{1}{2}\left[\frac{{\delta }_{k(i)}^{m}-{\delta }_{k(i-1)}^{l}}{{\sigma }_{k}}\right]}^{2}\right)+{c}_{1}\right]$$

Where ${\delta }_{k\left(i\right)}^{m}$ is the chemical shift of resonance *k*(*i*) (either Cα(*i*), Cβ(*i*) or CO(*i*)) of spin system *m* and ${\delta }_{k\left(i-1\right)}^{l}$ is the chemical shift of resonance *k*(*i*−1) (either Cα (*i* −1), Cβ (*i* −1) or CO(*i* −1)) of spin system *l*). ${\sigma }_{k}$ is related to the estimated precision of the measured chemical shifts. The *E*^*adj*^ is the sum of inverted Gaussians when *c*_0_ < 0. Previous assignment algorithms have used functions of this form to good effect for estimating adjacency^26^. In the limit of well-matched connectivities, the sum of inverted Gaussian functions will have a minimum value of *K(c*_0_ + *c*_1_) where *K* is the number of connectivities whereas, for poorly matched putative connectivities, the adjacent energy will tend to a limit of *Kc*_1_. Importantly, when an expected element of spin system *m* or *l* is missing, that contribution to the adjacency energy is set to zero. Similarly, if the subsequent position in the sequence is not currently assigned a spin system, then *E*^*adj*^ = 0. Here, *c*_0_ and *c*_1_ were set to −100 and +50, respectively. This results in an energy of −50 if the difference in chemical shifts is 0 and approaches +50 as the magnitude of the difference in chemical shifts approaches infinity. The value ${\sigma }_{k}$ is influenced by the properties of the NMR spectra from which the spin systems are built. For all runs described, ${\sigma }_{k}$ was chosen so that the function has an abscissa-intercept at a chemical shift difference of 0.2 ppm for all nuclei *k*.

The second term of the spin system energy, ${E}_{m}^{cs}$, evaluates the degree of correspondence of the observed chemical shifts to those predicted. It is this term that makes use of the ability of Bayesian statistics to incorporate diverse degrees of knowledge of the local structure of the protein. These include relatively structureless information encoded in the simple empirical distributions of chemical shifts of the amino acids observed in proteins or specific chemical shift predictions based on the high-resolution structure of the protein being examined. For the former, we utilize the BMRB^52^ database. For the latter, we use SHIFTX + predictions derived from either crystallographic structures available in the PDB^53^ or structures predicted by AlphaFold2^42^. Or in the case of the IDPs V5dm and hIDD, we use calculated, sequence-specific random coil chemical shifts^36,37^ as prediction. ${E}_{m}^{{cs}}$ is ultimately calculated from the Bayesian posterior probability of a proposed assignment given the observed chemical shifts:

8 $$P\left({A}_{n,m}\cap {Q}_{{m}_{i}}{{{{{\rm{|}}}}}}{B}_{m}\right)=\frac{P\left({B}_{m}{{{{{\rm{|}}}}}}{A}_{n,m}\cap {Q}_{{m}_{i}}\right)P\left({A}_{n,m}\cap {Q}_{{m}_{i}}\right)}{P\left({B}_{m}\right)}$$

The subscripts *n* and *m* index over all residue positions and the provided spin systems, respectively. The condition *A*_*n,m*_ refers to where spin system *m* is correctly assigned to sequence position *n*. The condition *B*_*m*_ refers to the observed chemical shifts of spin system *m*. Condition ${Q}_{{m}_{i}}$ refers to where resonance type set *i* of spin system *m* is the correct resonance type set. Because it is possible for the spin system to have ambiguous resonance crosspeak types, the probability calculation explicitly considers each resonance type set of a spin system within the context of each residue location. Thus, an assignment entails both the placement of a spin system at a residue location and choice of resonance type set.

The prior probability $P\left({A}_{n,m}\cap {Q}_{{m}_{i}}\right)$ refers to the initial probability of the assignment of spin system *m* to residue *n* being correct and that the resonance type set *i* is correct for spin system *m*. If *I*^*m*^ represents the number of possible resonance type sets of spin system *m* then the total number of combinations of residue type sets and residue locations for spin system *m* is the product *I*^*m*^*N*. However, given the constraints provided by the amino acid sequence of the protein, not all combinations of sequence location and residue type sets are possible. For example, a resonance type set with a defined amide proton would be impossible to place at a proline. To encode the impossibility of certain resonance type set/residue location combinations, these assignments are assigned a prior probability of 0. The remaining prior probability is then evenly distributed among the remaining locations:

9 $$P({A}_{n,m}\cap {Q}_{{m}_{i}})=\bigg\{\begin{array}{c}0;{{{{{\rm{at}}}}}}\,{{{{{\rm{all}}}}}}\,{{{{{\rm{values}}}}}}\,n,\,{m}_{i}\,{{{{{\rm{that}}}}}}\,{{{{{\rm{are}}}}}}\,{{{{{\rm{impossible}}}}}}\\ \frac{1}{C};{{{{{\rm{at}}}}}}\,{{{{{\rm{all}}}}}}\,{{{{{\rm{possible}}}}}}\,{{{{{\rm{combinations}}}}}}\,{{{{{\rm{of}}}}}}\,n,{m}_{i}\end{array}$$

Where *C* is the number of possible combinations of *n* and ${m}_{i}$ in the sequence.

The likelihood of assignment $P\left({B}_{m}|{A}_{n,m}\cap {Q}_{{m}_{i}}\right)$ (i.e., the probability of observing the chemical shifts of spin system *m* given the assignment ${A}_{n,m}\cap {Q}_{{m}_{i}}$) is given by Eq. 10 & 11:

10 $$P\left({B}_{m}|{A}_{n,m}\cap {Q}_{{m}_{i}}\right)={CCDF}\left({X}_{n,m,i}^{2}\right)$$

11 $${X}_{n,{m}_{i}}^{2}=\,\mathop{\sum }\limits_{r=1}^{R}{\left(\frac{{\delta }_{{obs},r}^{{m}_{i}}-{\delta }_{{pred},r}^{n}}{{\sigma }_{r}^{n}}\right)}^{2}$$

Where ${\delta }_{{pred},r}^{n}$ is the predicted chemical shift of spin *r* at sequence position *n*; ${\delta }_{{obs},r}^{{m}_{i}}$ is the observed chemical shift of resonance *r* of resonance type set *i* of spin system *m* and ${\sigma }_{r}^{n}\,$is the standard error for the chemical shift prediction of resonance *r* at sequence position *n*. The resonances, represented by variable *r*, are the following: H, N, C*α*, C*β*, CO, C(i-1), C*β*(i-1), CO(i-1). In Eqs. 10 and 11 it is assumed that the random variable ${\delta }_{{pred},r}^{n}\,$ is normally distributed about ${\delta }_{{obs},r}^{m}$ with a standard deviation ${\sigma }_{r}^{n}$ and that the error in the chemical shift measurement is much less than the error in the prediction. With these assumptions, the random variable ${X}_{n,m}^{2}$ is a chi-square distribution with *R* degrees of freedom, where *R* is equal to the number of spins for which data are provided. The likelihood is then calculated as the value of the complementary cumulative distribution function (CCDF) of a chi square variable of R degrees of freedom at ${X}_{n,{m}_{i}}^{2}$.

The likelihoods of all other residue position/resonance type sets being a valid assignment of spin system *m* are considered via the calculation of the marginalization, ${{{{{\rm{P}}}}}}\left({B}_{m}\right)$:

12 $${{{{{\rm{P}}}}}}\left({B}_{m}\right)={\sum }_{i=1}^{{I}^{m}}\mathop{\sum }\limits_{n=1}^{N}\left[P\left({B}_{m}|{A}_{n,m}\cap {Q}_{{m}_{i}}\right)P({A}_{n,m}\cap {Q}_{{m}_{i}})\right]$$

Where the summation terms are over all possible *i* and *n* combinations. Using Bayes’ theorem as expressed above, the posterior probability (i.e., the probability of a particular assignment being correct given the observed data) can be calculated and then ${E}_{m}^{cs}$ determined via:

13 $${E}_{m}^{cs}=\frac{{E}_{{{{{\rm{min}}}}}}^{cs}}{{{{{{\rm{log}}}}}} ({I}^{m}N)}\,{{{{{\rm{log}}}}}} (P({A}_{n,m}\cap {Q}_{{m}_{i}}|{B}_{m}){I}^{m}N)$$

To avoid numerical instability in the evaluation of logarithms of numbers near zero and to prevent a dominating influence of inaccurate chemical shift predictions on the energy function, instances where ${E}_{m}^{{cs}}$ > ${E}_{{{{{\rm{max}}}}}}^{{cs}}$ are fixed at ${E}_{\max }^{{cs}}$. ${E}_{\max }^{{cs}}$ and ${E}_{\min }^{{cs}}$ are set to 100 and −50 respectively, for this study.

The values of the parameters for the energy function were chosen to safeguard against inaccurate chemical shift predictions based on the following reasoning: a perfectly matching connectivity between two resonances will contribute −50 to the final energy function. Given that a spin system with a posterior probability of 0 will contribute 100 to the final energy function, it would require two perfect connectivities or three or more reasonable connectivities for that spin system to be favorably assigned to that position vs being left in the cache. This was done to permit the algorithm to assign a spin system to a particular location in the event of highly inaccurate chemical shift prediction of its resonances so long as there are sufficient resonance connectivities to justify the assignment. Likewise, the ${E}_{{{{{\rm{min}}}}}}^{{cs}}$ parameter was chosen such that a posterior probability of 1.0 would result in an energy contribution of −50 and would be equal to the contribution of a single perfect connectivity. This would require two bad connectivities to overrule a high posterior probability and disfavor its assignment. The user has control over these ${E}_{{{{{\rm{max }}}}}}^{{cs}}$ and ${E}_{{{{{\rm{min}}}}}}^{{cs}}$ to adjust the relative influence of chemical shift energy on the course of the annealing run.

The source of predicted shifts for each resonance can be from any source, so long as the precision of the prediction algorithm is accurately estimated. For IDPs, sequence-specific random coil chemical shifts can be substituted (see below). In the absence of an acceptable structural model, the average and standard deviation of the BMRB distribution of chemical shifts for a given atom of a given residue type are used as the predicted shift and prediction error respectively. This is also used in regions where the sequence of interest contains a tag that is absent in the structural model used to predict chemical shifts as well as regions that are not resolved.

### Generation of predicted chemical shifts

Predicted H, N, C*α*, C*β*, and CO chemical shifts were generated via SHIFTX + using PDB entries and/or AlphaFold2 predicted structures (Table 1). Chemical shift prediction errors for H, N, C*α*, Cβ, and CO were taken from the reported root mean squared deviations (RMSD) of SHIFTX + predictions: 0.45, 2.4, 0.8, 0.95, and 0.9 ppm, respectively. Sequence regions present in the NMR sample but not resolved or present in the provided structure (e.g., loops or expression tags) were given predicted values from their corresponding average values in the BMRB. For the runs that were performed with SPARTA+^38^ predicted shifts, the reported errors for each individual prediction were used. For the IDPs V5dm and hIDD, predicted shifts were provided using predicted sequence-specific random coil chemical shifts according to the method in^36,37^ Prediction errors were taken from the reported RMSD of the prediction method and were 0.16, 1.0, 0.42, 0.37, and 0.43 ppm for H, N, C*α*, Cβ, and CO resonances, respectively. Prediction errors associated with BMRB-derived values were taken as the standard deviation of the corresponding resonance distribution for the particular amino acid type in the BMRB.

### Comparison of resonance assignment algorithms

BARASA was compared to three triple resonance assignment algorithms that are highly utilized by the NMR community. All algorithms were provided the same crosspeak lists as BARASA, albeit in different file formats. As FLYA can utilize predicted chemical shift data, the algorithm was provided with the same predicted shifts and associated errors as BARASA. Assignment results were taken from the strong assignments generated from 20 runs. The assignment algorithm I-PINE was run using the I-PINE server. AutoAssign was run on NMRbox using the default parameters. For each algorithm, proposed assignments were compared to reference assignments. At each residue position, the proposed assignment was determined to have either matched, mismatched, or been missing when compared to the reference assignments (see Results and Discussion). The same reference assignments were used for the evaluation of all algorithms.

### Generation of simulated data sets

To assess the performance of BARASA on datasets of lower quality, the MBP crosspeak lists were processed to randomly retain spin systems and/or individual crosspeaks at specific probabilities depending on cross peak type. For each data quality condition 10 different independent data sets were randomly generated and BARASA was run on each of them. The results from each of these executions of BARASA were generated from the curation of 20 independent annealing runs. The performance of BARASA on data with artifactual peaks was evaluated using a depleted data set and adding randomly generated cross peaks such that 20% of all C*α*, Cβ, and CO cross-peaks were artifacts. Each artifact peak was generated in the following manner. A random residue from the protein sequence, containing an amide group and desired peak type (C*α*, Cβ, CO, C(i-1), Cβ(i-1), or CO(i-1)) was chosen. Chemical shifts for each dimension of the cross peak were randomly generated from a Gaussian distribution with a mean and standard deviation equal to the mean and standard deviation value of that atom of that residue type in the BMRB. All artifact peaks were given the maximum peak intensity of their peak lists to ensure they would not be cached during the run.

BARASA was implemented in C++ and can be built on all major computing platforms (MacOS, Linux, and Windows). BARASA possesses a command line interface, as well as a GUI implemented using the wxWidgets library and utilizes the Boost libraries. For this study, the simulations were run on 2019 6-core MacBook Pro (Intel processor) with up to 12 annealing runs running in parallel.

### Reporting summary

Further information on research design is available in the Nature Portfolio Reporting Summary linked to this article.

## Supplementary information

Supplementary Information

Reporting Summary

## Data Availability

Resonance assignments for IGPS and IL-1Ra have been deposited to the BMRB under accession numbers 51347, and 51352, respectively. Cross peak lists and protein sequences for IL-1β, IL-1Ra, IGPS, MBP, Cy1, ecTS, v5domain, and huIDR, which form the foundation of the analysis here, are included in the Source Data file. BMRB statistics used to test BARASA are also included in the Source Data file. Assignments referenced in this study from the BMRB can be accessed via the following accession codes: 434, 4354, 19082, 18927, and 28135. The experimental structures referenced in this study from the PDB can be accessed via the following accession codes: 9ILB, 2IRT, 1IGS, 1DMB, 7RY6, and 1AOB. Supplementary Information is available and consists of fifteen tables and one figure listing resonance assignments made by BARASA, summary statistics of BARASA’s performance using SPARTA+ predicated chemical shifts, AlphaFold2 structural models or in the presence of artifact peaks. Source data are provided with this paper.

## Source data

Source Data

## Code availability

BARASA will be made generally available for non-commercial use through, preferably, NMRbox^50^ [https://nmrbox.nmrhub.org/] or, less preferred, by contacting barasa@wandlab.org for Linux or OSX compatible executables.
