# Supplementary material for: Robust automated backbone triple resonance NMR assignments of proteins using Bayesian-based simulated annealing
Source: Nat Commun. 2023 Mar 21;14:1556. doi: 10.1038/s41467-023-37219-z (PMC10030768; doi:10.1038/s41467-023-37219-z)
Supplement: Supplementary file 1 — Supplementary Information [file 41467_2023_37219_MOESM1_ESM.pdf]

Supplementary Information

Robust Automated Backbone Triple Resonance NMR Assignments of Proteins Using Bayesian-Based Simulated Annealing

Anthony C. Bishop[1], Glorisé Torres-Montalvo[1], Sravya Kotaru[4], Kyle Mimun[1] and A. Joshua Wand[1,2,3,4*]

Departments of Biochemistry & Biophysics,[1] Chemistry[2] and Molecular & Cellular Medicine[3], Texas A&M University, College Station, Texas USA 77843

[4]Graduate Group in Biochemistry & Molecular Biophysics, Perelman School of Medicine, University of Pennsylvania, Philadelphia, Pennsylvania USA 19014

*E-mail: josh.wand@ag.tamu.edu

Supplementary Table 1 – Acquisition parameters for NMR assignment spectra

Spectrum			IL-1β	IL1Ra	IGPS	MBP‡
HSQC[1]	^{1}H (direct)	SW (ppm)	13.58	14.87	16.04	15.62
		pts	1024	1024	1024	2048
	^{15}N	SW (ppm)	30.0	27	30.0	36.0
		pts	128	82	128	128
	Field (MHz)		800	800	750	800
	NS		8	8	8	32
HNCA[2]	^{1}H (direct)	SW (ppm)	20.82	14.87	16.04	15.62
		pts	1024	1024	1024	2048
	^{15}N	SW (ppm)	30.0	29	30.0	36.0
		pts	50†	50†	52†	64†
	^{13}C	SW (ppm)	33	30	28	29.0
		pts	72†	128†	74†	98†
	Field (MHz)		800	800	750	800
	NS		8	16	32	32
HN(CO)CA[2]	^{1}H (direct)	SW (ppm)	20.82	-	16.04	15.62
		pts	1024	-	1024	2048
	^{15}N	SW (ppm)	30.0	-	30.0	36.0
		pts	50†	-	52†	64†
	^{13}C	SW (ppm)	33	-	28	29.0
		pts	72†	-	74†	98†
	Field (MHz)		800	-	750	800
	NS		8	-	32	16
HNCACB[3*]	^{1}H (direct)	SW (ppm)	20.82	14.87	16.04	15.62
		pts	1024	1024	1024	2048
	^{15}N	SW (ppm)	30	29	30.0	36.0
		pts	54†	56†	44†	64†
	^{13}C	SW (ppm)	70.0	65	65	63.0
		pts	296†	64†	60†	214†
	Field (MHz)		800	800	750	800
	NS		16	16	88	32
CBCA(CO)NH[3*]	^{1}H (direct)	SW (ppm)	20.82	14.87	16.04	15.62
		pts	1024	1024	1024	2048
	^{15}N	SW (ppm)	30	29	30.0	36.0
		pts	54†	56†	44†	64†
	^{13}C	SW (ppm)	70.0	65	65	63.0
		pts	296†	64†	60†	214†
	Field (MHz)		800	800	750	800
	NS		48	16	88	32
HNCO[4†]	^{1}H (direct)	SW (ppm)	20.82	14.87	16.04	15.62
		pts	1024	1024	1024	2048
	^{15}N	SW (ppm)	30.0	29	30.0	36.0
		pts	50†	64	52†	64†
	^{13}C	SW (ppm)	12.0	16	12.0	13.0
		pts	72†	192	32†	44†
	Field (MHz)		800	500	750	800
	NS		8	16	32	16
HN(CA)CO[5]	^{1}H (direct)	SW (ppm)	20.82	14.87	-	15.62
		pts	1024	1024	-	2048
	^{15}N	SW (ppm)	30.0	29	-	36.0
		pts	50†	64	-	64†
	^{13}C	SW (ppm)	12.0	16	-	13.0
		pts	72†	192	-	44†
	Field (MHz)		800	500	-	800
	NS		32	32	-	64

SW = Sweep Width; pts = complex points; NS = scans/FID; ‡Spectra were acquired using TROSY[6] variants of the spectra; †Indirect dimensions acquired using NUS[8] with Poisson gap distribution sampling and reconstructed via hmsIST[7]; *HNCACB, HNCOCACB spectra acquired for IL-1β. TROSY-HNCOCACB and TROSY-HNCOCACB spectra were acquired for MBP; Field strengths are given as the proton Larmor frequency. The table is included in Excel format in the Source Data file.

Supplementary Table 2 – Performance of Various Automated Assignment Algorithms using SHIFTX+[8] predicted shifts from PDB[9] structures[‡]			
**BARASA**			
**Protein**	**Fraction Matching**	**Fraction Missing**	**Fraction Mismatching**
**IL-1β**	0.994	0.000	0.006
**IL-1Ra**	0.974	0.020	0.007
**IGPS**	0.952	0.040	0.008
**MBP**	0.981	0.005	0.013
**CY1**	0.927	0.046	0.026
**ecTS**	0.984	0.008	0.008
**VD5dm**	1.000	0.000	0.000
**hIDD**	0.789	0.028	0.183
**I-PINE[10]**			
**Protein**	**Fraction Matching**	**Fraction Missing**	**Fraction Mismatching**
**IL-1β**	0.896	0.000	0.104
**IL-1Ra**	0.921	0.013	0.066
**IGPS**	0.859	0.016	0.125
**MBP**	0.954	0.011	0.035
**CY1**	0.647	0.117	0.236
**ecTS**	0.939	0.004	0.057
**VD5dm**	0.045	0.136	0.818
**hIDD**	0.083	0.037	0.881
**FLYA[11]**			
**Protein**	**Fraction Matching**	**Fraction Missing**	**Fraction Mismatching**
**IL-1β**	0.929	0.039	0.033
**IL-1Ra**	0.960	0.033	0.007
**IGPS**	0.903	0.065	0.032
**MBP**	0.981	0.005	0.013
**CY1**	0.861	0.115	0.024
**ecTS**	0.943	0.049	0.008
**VD5dm**	0.985	0.015	0.000
**hIDD**	0.716	0.138	0.147
**AutoAssign[12]**			
**Protein**	**Fraction Matching**	**Fraction Missing**	**Fraction Mismatching**
**IL-1β**	0.838	0.161	0.000
**IL-1Ra**	0.712	0.253	0.035
**IGPS**	0.864	0.091	0.045
**MBP**	0.792	0.199	0.008
**CY1**	0.391	0.585	0.024
**ecTS**	0.789	0.140	0.072
**VD5dm**	0.697	0.242	0.061
**hIDD**	0.300	0.700	0.000

[‡] Used the comprehensive triple resonance data set described for each protein
The table is included in Excel format in the Source Data file.

Supplementary Table 3 – Assignments of IL-1β by BARASA

Index	Type	H	N	CA(i-1)	CA	CB(i-1)	CB	CO(i-1)	CO	Posterior	Likelihood	Prior
1	M	9.59	100.84	---	---	---	---	---	---	0.577	0.000	0.007
2	A	8.75	128.04	54.47	50.28	32.00	17.43	171.88	175.44	1.000	0.546	0.008
3	P											
4	V	8.16	119.66	62.56	61.00	31.35	32.46	176.34	175.01	0.618	0.893	0.008
5	R	9.05	128.44	61.00	56.10	32.39	30.37	175.01	175.49	0.999	0.912	0.008
6	S	8.38	118.52	56.11	56.74	30.36	65.29	175.47	172.80	0.381	0.812	0.004
7	L	9.09	122.46	56.74	53.68	65.32	44.37	172.80	175.01	0.516	0.807	0.008
8	N	8.88	120.69	53.69	51.48	44.35	38.90	175.00	175.61	0.985	0.969	0.008
9	C	9.66	117.81	51.47	55.53	38.87	32.41	175.60	172.18	0.984	0.139	0.008
10	T	8.98	109.43	55.52	59.53	32.36	71.26	172.19	174.06	1.000	0.877	0.008
11	L	9.49	120.46	59.55	53.28	71.32	45.67	174.06	176.58	0.991	0.411	0.008
12	R	8.66	119.30	53.28	53.84	45.65	34.10	176.48	---	0.500	0.950	0.008
13	D	8.78	121.19	53.86	52.90	34.04	40.32	176.38	179.86	0.999	0.315	0.008
14	S	7.45	114.53	52.86	60.13	40.29	61.56	179.86	---	1.000	0.228	0.008
15	Q	8.05	120.43	60.14	54.69	61.57	27.62	175.29	---	1.000	0.933	0.004
16	Q	8.46	111.39	54.73	57.03	27.64	24.45	175.27	175.21	0.246	1.000	0.001
17	K	8.47	120.05	57.03	58.69	24.35	29.26	175.20	175.69	1.000	0.060	0.008
18	S	8.30	119.34	58.76	---	29.26	64.93	175.69	171.93	0.684	0.007	0.004
19	L	8.88	121.99	58.85	54.21	64.91	41.36	171.93	175.87	0.990	0.690	0.008
20	V	8.83	115.24	54.22	58.60	41.32	35.31	175.88	174.78	0.993	0.883	0.004
21	M	8.74	120.21	58.60	55.20	35.26	31.10	174.78	176.61	0.938	0.985	0.008
22	S	8.67	119.76	55.21	56.29	31.09	62.34	176.61	174.41	0.117	0.206	0.004
23	G	8.18	111.94	56.29	44.24	62.35	---	174.40	171.07	1.000	0.026	0.004
24	P											
25	Y	8.08	110.34	63.71	55.62	30.72	38.65	176.56	175.12	0.993	0.252	0.004
26	E	7.20	119.23	55.49	---	38.64	33.59	175.12	173.05	0.205	0.371	0.004
27	L	8.70	122.51	55.41	52.67	33.58	44.32	173.05	176.57	0.551	0.677	0.008
28	K	9.25	119.20	52.66	54.68	44.30	36.56	176.57	173.81	0.763	0.990	0.008
29	A	7.65	118.66	54.67	49.07	36.51	21.01	173.81	177.81	0.678	0.003	0.008
30	L	9.48	125.65	49.06	55.24	20.94	43.35	177.81	175.34	0.719	0.021	0.008
31	H	10.27	121.81	55.24	59.29	43.33	27.16	175.34	174.30	1.000	0.008	0.008
32	L	8.45	126.06	59.31	53.31	27.13	43.79	174.30	176.70	0.999	0.030	0.008
33	Q	8.97	120.12	53.30	54.48	43.77	29.90	176.81	---	0.176	0.190	0.008
34	G	8.83	109.55	54.46	46.55	29.82	---	176.93	176.37	0.997	0.730	0.007
35	Q	8.97	121.95	46.54	56.78	---	27.44	176.36	176.76	0.948	0.968	0.004
36	D	7.98	118.91	56.80	54.83	27.44	40.30	176.76	177.04	0.093	0.510	0.001
37	M	7.69	118.33	54.85	57.06	40.27	30.85	177.04	177.67	0.983	0.448	0.008
38	E	8.12	117.33	57.21	---	30.86	27.97	177.68	176.97	0.490	0.808	0.004

39	Q	7.99	117.04	57.32	56.02	27.90	28.73	176.98	176.16	0.771	0.876	0.008
40	Q	7.56	117.06	56.06	---	28.61	29.09	176.15	176.17	0.118	0.901	0.002
41	V	8.39	122.86	56.10	61.75	29.02	31.71	176.13	174.07	0.999	0.850	0.008
42	V	7.78	123.69	61.75	61.30	31.56	---	174.07	176.09	0.864	0.334	0.007
43	F	9.66	127.22	61.28	56.92	31.29	40.22	176.10	---	0.562	0.294	0.008
44	S	9.21	115.39	56.91	56.22	40.23	62.93	176.13	174.49	0.938	0.728	0.004
45	M	9.67	101.77	56.23	53.86	62.95	34.45	174.51	174.89	0.000	0.000	0.008
46	S	8.91	120.78	53.88	56.63	34.43	64.55	174.89	173.20	0.539	0.669	0.008
47	F	8.52	122.94	56.63	58.52	64.63	37.38	173.20	176.41	0.984	0.922	0.008
48	V	7.45	118.63	58.52	60.07	37.34	32.54	176.40	175.23	0.834	0.904	0.008
49	Q	8.59	120.32	60.07	55.73	32.54	28.95	175.22	176.13	0.850	0.974	0.004
50	G	8.42	111.81	55.72	44.71	28.92	---	176.13	172.74	0.999	0.904	0.004
51	E	8.56	121.46	44.71	56.06	---	29.22	172.74	176.17	0.575	0.994	0.004
52	E	8.61	122.59	56.03	55.71	29.20	30.02	176.17	175.89	0.200	0.988	0.002
53	S	8.49	116.89	55.74	57.18	30.00	63.64	175.89	174.29	0.510	0.937	0.004
54	N	8.64	120.27	57.28	53.86	63.56	38.01	174.20	174.82	0.993	0.834	0.008
55	D	8.59	116.72	53.85	54.49	---	39.89	174.83	175.39	0.236	0.552	0.004
56	K	7.74	118.92	54.43	55.15	39.91	33.69	175.38	175.06	0.458	0.624	0.008
57	I	8.50	122.90	55.29	56.86	33.78	39.43	175.00	173.99	0.549	0.718	0.004
58	P											
59	V	10.08	118.03	62.21	58.29	30.88	36.50	177.46	173.75	0.998	0.608	0.008
60	A	8.70	120.53	58.33	50.26	36.45	22.50	173.75	176.28	0.995	0.958	0.008
61	L	10.58	124.04	50.24	53.46	22.39	44.22	176.28	173.34	0.063	0.000	0.008
62	G	8.28	109.86	53.45	42.34	44.22	---	173.34	173.20	0.275	0.408	0.002
63	L											
64	K											
65	E	8.97	118.71	58.40	58.09	---	---	177.38	176.29	0.420	0.446	0.007
66	K	7.61	115.22	57.90	---	---	---	176.30	---	0.080	0.545	0.007
67	N											
68	L	6.87	115.63	53.86	52.56	---	46.64	173.70	---	0.972	0.053	0.004
69	Y	9.22	121.28	52.59	56.75	46.64	42.25	175.31	175.89	1.000	0.689	0.008
70	L	8.52	121.60	56.74	56.26	42.24	41.23	175.88	176.52	0.999	0.941	0.008
71	S	9.20	115.80	56.27	55.67	41.19	66.46	176.52	173.21	0.955	0.774	0.008
72	C	8.45	119.49	55.65	56.33	66.50	29.66	173.21	174.29	0.972	0.546	0.008
73	V	8.86	117.24	56.33	58.37	29.69	35.37	174.23	---	0.968	0.834	0.004
74	L	8.61	123.64	58.37	54.13	35.32	41.81	174.14	176.72	0.667	0.985	0.008
75	K	8.27	126.25	54.12	55.37	41.78	33.31	176.72	176.05	0.754	0.812	0.008
76	D	9.37	127.94	55.46	---	33.26	38.59	176.05	174.94	0.234	0.439	0.008
77	D	8.58	109.58	55.51	---	38.54	39.45	174.95	174.53	0.952	0.941	0.008
78	K	7.73	120.09	55.41	52.76	39.45	33.90	174.53	173.98	0.629	0.749	0.004
79	P											

80	T	8.80	119.77	62.97	61.82	30.51	72.22	175.00	171.78	1.000	0.812	0.004
81	L	8.92	126.79	61.86	53.61	72.19	43.51	171.78	175.62	0.999	0.973	0.008
82	Q	9.76	126.37	53.40	---	43.49	31.21	175.62	173.66	0.245	0.114	0.008
83	L	8.56	122.02	53.28	54.00	31.14	41.26	173.67	175.22	0.886	0.793	0.008
84	E	9.21	123.77	53.98	54.34	41.25	32.79	175.22	175.48	0.106	0.992	0.002
85	S	8.81	121.40	54.36	58.26	32.77	63.01	175.49	174.70	0.310	0.992	0.004
86	V	7.98	117.40	58.19	---	63.00	34.33	174.69	---	0.786	0.977	0.004
87	D	8.18	121.91	58.13	50.67	34.26	41.41	174.67	176.28	0.986	0.460	0.008
88	P											
89	K	8.44	117.12	63.62	57.45	31.30	---	177.84	177.77	0.204	0.896	0.002
90	N	7.81	114.23	57.47	52.52	31.09	40.84	177.76	172.74	0.961	0.123	0.008
91	Y	7.30	116.46	52.51	55.99	40.73	---	172.74	173.76	0.498	0.170	0.007
92	P											
93	K	7.28	116.75	62.44	54.46	33.74	---	174.24	174.53	0.257	0.614	0.002
94	K	8.35	119.21	54.48	58.81	33.87	31.83	174.55	176.41	0.863	0.979	0.004
95	K	7.91	118.02	58.80	53.95	31.78	30.30	176.42	175.92	0.571	0.814	0.004
96	M	7.69	122.46	53.84	---	30.27	33.39	175.93	---	0.878	0.726	0.008
97	E	9.17	122.46	53.75	58.95	33.34	27.50	175.96	177.44	0.991	0.138	0.008
98	K	8.28	121.51	58.95	59.39	27.45	31.32	177.44	178.27	1.000	0.138	0.008
99	R	7.96	113.99	59.42	57.85	31.35	27.17	178.27	176.28	0.909	0.709	0.004
100	F	7.89	116.53	57.84	57.44	27.18	40.57	176.28	175.31	0.870	0.326	0.004
101	V	7.48	117.74	57.43	62.96	40.55	31.12	175.31	175.35	0.232	0.354	0.002
102	F	9.88	127.00	62.96	55.58	31.05	41.47	175.36	174.19	0.894	0.656	0.008
103	N	10.44	120.98	55.58	51.96	41.47	38.83	174.19	173.80	0.990	0.524	0.004
104	K	9.39	127.72	51.95	55.86	38.77	30.97	173.80	176.03	0.998	0.868	0.008
105	I	9.32	103.98	55.82	60.18	30.97	40.53	176.02	174.42	0.000	0.000	0.004
106	E	8.57	126.53	60.18	54.58	40.51	29.94	174.42	175.64	0.997	0.494	0.008
107	I	8.72	125.66	54.58	59.02	29.84	39.37	175.63	175.58	0.103	0.443	0.002
108	N	9.43	126.70	59.02	54.30	39.33	36.69	175.48	174.43	0.996	0.014	0.008
109	N	8.90	110.32	54.29	54.69	36.59	37.20	174.43	173.98	0.010	0.003	0.008
110	K	8.06	120.24	54.73	54.43	37.15	34.70	173.98	174.59	0.122	0.127	0.004
111	L	9.50	121.00	54.37	53.12	34.70	44.55	174.59	176.26	0.525	0.841	0.004
112	E	8.82	117.55	53.07	53.00	44.56	31.66	176.26	174.37	0.038	0.435	0.000
113	F	10.37	119.37	52.98	55.94	31.55	40.92	174.37	173.88	0.952	0.369	0.004
114	E	8.85	126.06	55.96	53.03	40.87	32.15	173.88	177.31	0.666	0.788	0.004
115	S	9.20	121.84	53.01	58.79	32.07	62.52	177.31	---	0.669	0.441	0.008
116	A	8.18	128.30	58.79	53.19	62.58	17.48	174.76	179.25	1.000	0.521	0.008
117	Q	7.92	117.02	53.19	56.33	17.59	28.43	179.26	174.76	0.923	0.602	0.008
118	F	7.45	116.97	56.32	52.90	28.39	38.10	174.76	181.39	0.000	0.000	0.004
119	P											
120	N	10.22	113.76	63.08	55.31	30.99	37.43	178.17	173.33	0.999	0.722	0.004

121	W	8.15	119.95	55.30	56.79	37.40	28.40	173.33	175.28	0.999	0.244	0.008
122	Y	9.47	119.16	56.78	56.41	28.41	41.14	175.28	179.05	0.227	0.004	0.004
123	I	8.78	124.75	56.38	65.56	41.11	35.92	179.05	175.28	1.000	0.003	0.008
124	S	9.02	122.28	65.56	57.19	35.90	66.73	175.28	171.32	1.000	0.337	0.004
125	T	9.07	109.86	57.18	58.40	66.71	70.26	171.33	174.69	0.992	0.496	0.004
126	S	9.65	113.87	58.41	56.52	70.25	63.64	174.69	175.96	1.000	0.219	0.004
127	Q	8.98	121.94	56.51	57.96	63.66	28.04	175.96	177.66	0.998	0.843	0.004
128	A	8.22	120.90	57.94	51.06	28.06	19.09	177.66	176.57	0.779	0.826	0.004
129	E	8.54	116.95	51.06	55.90	19.01	30.23	176.57	177.42	0.999	0.449	0.008
130	N	8.09	113.80	55.89	54.46	30.22	37.42	177.42	173.53	0.680	0.558	0.004
131	M	8.94	117.88	54.44	51.60	37.39	30.58	173.53	174.25	0.998	0.948	0.008
132	P											
133	V	8.11	125.70	62.48	---	31.90	31.25	178.77	175.88	0.929	0.378	0.008
134	F	8.80	124.86	62.45	55.29	31.20	41.36	175.87	171.89	0.914	0.997	0.008
135	L	8.71	119.79	55.28	52.98	41.35	42.80	171.89	176.40	0.999	0.913	0.008
136	G	9.35	114.89	52.97	44.82	42.77	---	176.39	173.18	0.932	0.564	0.007
137	G	8.76	110.19	44.80	44.04	---	---	173.19	174.73	0.425	0.204	0.007
138	T	7.49	112.21	44.01	61.05	---	69.65	174.73	174.20	1.000	0.512	0.004
139	K	8.50	124.63	61.05	55.89	69.75	31.91	174.19	177.24	1.000	0.373	0.008
140	G	8.76	111.69	55.89	45.13	31.88	---	177.24	174.93	0.753	0.259	0.004
141	G	8.20	109.21	45.10	44.56	---	---	174.94	174.35	0.673	0.821	0.007
142	Q	8.52	119.62	44.56	56.11	---	28.81	174.35	176.24	0.510	0.760	0.004
143	D	7.90	119.42	56.08	54.00	28.79	40.78	176.24	175.39	0.869	0.853	0.004
144	I	9.28	125.65	53.99	61.26	---	---	175.38	---	0.488	0.388	0.007
145	T											
146	D	7.06	120.46	---	52.59	---	---	173.09	---	0.137	0.785	0.002
147	F	9.63	117.80	52.58	56.27	42.61	43.47	174.08	176.07	0.280	0.524	0.004
148	T	9.62	109.71	56.27	60.19	43.45	70.76	176.06	173.25	1.000	0.871	0.008
149	M	8.76	121.31	60.20	53.46	70.87	36.49	173.25	174.86	0.931	0.817	0.008
150	Q	8.49	123.81	53.45	54.02	36.46	29.27	174.93	---	0.871	0.287	0.004
151	F	8.83	123.42	54.01	58.32	29.20	38.58	175.00	176.14	0.783	0.516	0.008
152	V	8.10	123.04	---	61.04	38.53	32.90	176.15	175.51	0.410	0.218	0.003
153	S	8.50	119.48	61.04	58.36	32.84	63.27	175.51	173.70	1.000	0.406	0.008
154	S	8.10	122.61	58.36	59.71	63.33	64.12	173.70	178.52	1.000	0.009	0.008

Assigned amide groups that match the reference assignment are indicated by green shading. Missing amide groups that are present in the reference assignments are indicated by white shading. Amide groups that were assigned to residues that are not assigned in the reference assignments are indicated by orange shading. Missing amide groups that were assigned in BMRB 434[13], but not present in the spectra are indicated by grey shading and are considered matching the reference assignments. Residues that were not assigned by either BARASA or in the reference assignments were considered matching. Non-amide resonances were not considered when determining assignment accuracy. The table is included in Excel format in the Source Data file.

Supplementary Table 4 – Assignments of IL1-Ra by BARASA												
**Index**	**AA**	**H**	**N**	**CA(i-1)**	**CA**	**CB(i-1)**	**CB**	**CO(i-1)**	**CO**	**Posterior**	**Likelihood**	**Prior**
1	R											
2	P											
3	S											
4	G											
5	R											
6	K											
7	S											
8	S	9.07	116.95	59.70	58.28	60.03	---	---	---	0.589	0.004	0.004
9	K	8.25	122.67	58.39	56.32	64.25	33.45	173.64	175.35	1.000	0.173	0.008
10	M	8.03	120.67	56.36	55.37	33.44	35.28	175.34	175.47	0.010	0.048	0.002
11	Q	8.98	124.66	55.35	54.74	35.20	33.35	175.54	174.20	0.150	0.113	0.008
12	A	8.47	126.24	54.68	51.56	33.34	21.16	174.20	176.53	0.763	0.831	0.008
13	F	9.61	122.98	51.60	56.88	21.11	43.65	176.53	175.34	0.700	0.833	0.008
14	R	8.99	119.14	56.66	54.69	43.74	33.14	175.34	174.86	0.689	0.945	0.008
15	I	9.00	120.40	54.69	58.64	33.13	43.57	174.86	173.09	0.665	0.696	0.008
16	W	8.51	123.73	58.64	56.74	43.44	32.18	172.44	175.79	0.936	0.406	0.008
17	D	9.32	120.19	56.73	52.99	32.16	42.07	175.54	178.37	0.886	0.484	0.008
18	V	6.66	109.26	53.02	64.27	42.06	31.37	178.37	176.19	0.870	0.001	0.008
19	N											
20	Q	8.79	113.37	52.96	58.06	38.73	25.69	175.00	174.63	0.984	0.572	0.008
21	K	8.54	119.25	57.94	---	25.64	32.38	174.63	176.45	0.581	0.933	0.008
22	T	8.84	113.77	57.82	60.02	32.35	71.42	176.45	174.18	0.590	0.947	0.008
23	F	9.45	122.17	60.07	53.57	71.47	38.99	174.18	175.43	0.657	0.252	0.008
24	Y	9.01	116.21	53.56	56.28	38.99	41.09	175.43	172.26	0.988	0.326	0.008
25	L	9.08	120.67	56.27	53.72	41.09	43.93	172.26	176.76	0.911	0.976	0.008
26	R	8.80	123.83	53.73	55.35	43.94	33.27	176.76	176.11	0.432	0.929	0.008
27	N	9.51	127.83	55.28	54.81	33.22	37.19	176.11	174.36	0.767	0.852	0.008
28	N	8.70	110.17	54.72	---	37.06	37.81	174.36	172.85	0.975	0.931	0.004
29	Q	7.57	116.74	54.71	53.97	37.90	31.86	172.84	174.01	0.578	0.903	0.008
30	L	8.65	124.81	53.94	54.43	31.83	42.54	173.88	175.64	0.319	0.556	0.008
31	V	9.05	125.59	54.77	59.70	42.53	35.03	175.64	172.72	0.306	0.120	0.008
32	A	7.14	118.49	59.73	48.87	34.97	21.69	172.72	179.11	0.535	0.000	0.008
33	G	9.73	113.18	48.89	45.57	21.63	---	179.10	169.01	0.998	0.000	0.007
34	Y	9.12	121.11	45.58	57.04	---	38.70	169.02	175.32	0.836	0.395	0.002
35	L	8.56	125.11	57.09	53.93	38.71	42.34	175.32	175.95	0.716	0.968	0.008
36	Q	8.30	119.68	53.94	54.30	42.33	31.99	175.95	---	0.116	0.544	0.004
37	G	8.79	111.73	54.30	45.11	31.94	---	175.77	---	0.654	0.020	0.004
38	P											

39	N	7.60	114.33	64.11	53.66	31.92	38.08	177.45	176.05	0.454	0.905	0.008
40	V	7.35	116.00	53.69	65.03	38.05	31.21	176.05	177.17	0.948	0.789	0.008
41	N	7.68	117.43	65.06	54.46	31.13	37.88	177.17	175.55	0.340	0.658	0.008
42	L	7.75	118.66	54.50	54.92	37.86	41.98	175.55	176.30	0.812	1.000	0.008
43	E	7.06	119.35	55.01	57.42	41.96	30.26	176.30	176.93	0.492	0.981	0.008
44	E	8.79	127.31	57.42	54.93	30.19	29.66	176.93	176.56	0.528	0.937	0.008
45	K	7.94	122.93	54.86	55.79	29.39	33.24	176.56	175.71	0.474	0.901	0.008
46	I	8.99	121.13	55.85	57.30	33.22	38.08	175.71	175.34	0.515	0.711	0.008
47	D	8.98	128.28	57.33	53.42	38.02	43.71	175.33	174.52	0.575	0.572	0.008
48	V	8.86	119.56	53.46	60.24	43.75	35.83	174.52	176.26	0.827	0.714	0.008
49	V	8.03	124.51	60.22	59.02	35.73	---	176.25	173.91	0.408	0.753	0.007
50	P											
51	I	8.35	123.37	62.43	62.72	31.70	40.08	176.60	175.47	0.432	0.818	0.004
52	E	8.09	118.30	62.84	54.48	39.89	29.84	175.46	173.85	0.935	0.225	0.008
53	P											
54	H	8.39	118.52	63.25	55.90	33.49	29.43	176.86	172.57	0.144	0.085	0.008
55	A	8.19	121.89	55.90	50.50	29.39	22.33	172.57	176.77	0.938	0.278	0.008
56	L	9.19	118.15	50.49	55.09	22.33	44.18	176.76	176.14	0.140	0.122	0.002
57	F	8.53	119.81	55.10	56.40	44.21	40.58	176.13	174.93	0.306	0.191	0.008
58	L	9.91	122.62	56.41	53.81	40.56	45.60	174.93	---	0.748	0.265	0.008
59	G	9.12	109.56	53.80	44.86	45.65	---	174.97	172.13	0.979	0.671	0.007
60	I	8.30	110.53	44.86	59.85	---	42.22	172.14	176.88	0.998	0.201	0.002
61	H	9.59	117.37	59.88	---	---	26.34	176.88	176.50	0.321	0.265	0.004
62	G	9.02	111.34	55.85	46.57	26.62	---	176.50	176.03	0.971	0.350	0.007
63	G	8.09	107.37	46.60	---	---	---	176.03	172.56	0.353	0.203	0.002
64	K	7.14	116.32	45.86	57.08	---	33.02	172.56	176.17	0.315	0.444	0.002
65	M	7.68	115.57	57.07	54.39	32.95	37.72	176.17	173.90	0.617	0.981	0.008
66	A	9.07	123.02	54.40	51.23	37.68	24.20	173.89	176.31	0.991	0.501	0.008
67	L	8.21	122.40	51.26	55.85	24.17	43.62	176.30	---	0.879	0.601	0.008
68	S	8.72	111.89	55.78	---	43.56	67.49	175.68	173.32	0.977	0.594	0.008
69	C	7.35	116.50	55.73	54.06	67.50	47.89	173.30	173.70	0.006	0.000	0.008
70	V	8.59	126.04	54.09	60.54	47.90	34.87	173.68	173.91	0.207	0.000	0.002
71	K	8.04	125.36	60.54	54.46	34.78	33.68	173.91	176.42	0.531	0.731	0.008
72	S	8.18	119.68	54.49	56.22	33.60	63.37	176.42	174.96	0.359	0.453	0.008
73	G	8.83	117.42	56.21	47.19	63.41	---	174.87	---	0.214	0.439	0.004
74	D	8.79	125.57	47.22	54.62	---	41.17	174.78	175.87	0.938	0.248	0.002
75	E	7.95	120.40	54.66	55.33	41.15	31.89	175.87	175.26	0.104	0.200	0.008
76	T	7.88	121.00	55.87	62.63	31.87	69.29	175.26	173.19	0.483	0.301	0.008
77	R	8.46	123.27	62.68	54.24	69.35	33.91	173.19	---	0.959	0.917	0.008
78	L	8.45	123.29	54.31	53.48	33.84	43.73	173.32	---	0.532	0.985	0.008

79	Q	8.96	127.09	53.48	54.14	43.78	32.23	176.26	173.11	0.367	0.178	0.008
80	L	8.14	123.32	54.15	53.75	32.18	43.49	173.11	176.74	0.563	0.976	0.008
81	E	9.14	123.19	53.66	55.03	43.49	33.58	176.73	175.58	0.451	0.935	0.008
82	A	8.79	127.80	55.03	51.77	33.53	16.77	175.57	175.11	0.915	0.584	0.008
83	V	7.57	124.40	51.76	60.13	16.75	34.97	175.11	171.73	1.000	0.084	0.008
84	N	7.93	119.63	60.15	51.42	34.92	38.47	171.73	178.20	0.090	0.012	0.008
85	I	8.54	121.85	51.47	64.39	38.35	---	178.20	176.80	0.672	0.063	0.007
86	T	7.72	109.70	64.46	63.69	37.93	68.40	176.80	174.91	0.821	0.987	0.008
87	D	7.66	120.36	63.66	58.17	68.45	40.49	176.77	176.42	1.000	0.016	0.008
88	L	6.51	119.96	54.45	---	41.35	42.42	176.41	177.13	0.993	0.679	0.004
89	S	9.75	116.14	54.47	56.88	42.49	64.99	177.13	175.53	0.927	0.369	0.008
90	E	9.70	127.08	56.84	57.98	65.03	29.10	175.52	---	1.000	0.938	0.008
91	N	8.10	116.07	57.98	53.28	29.15	39.35	175.63	174.72	0.679	0.901	0.008
92	R	7.41	120.62	53.26	55.67	39.35	31.21	174.70	---	0.553	0.623	0.008
93	K											
94	Q	9.07	116.96	60.03	58.42	32.08	28.03	179.20	176.73	0.591	0.742	0.008
95	D	7.58	120.14	58.38	55.09	27.99	40.45	176.73	177.22	0.524	0.629	0.008
96	K	7.57	121.06	55.10	58.87	40.44	31.95	177.13	---	0.377	0.826	0.008
97	R											
98	F	7.60	120.77	58.10	---	27.74	40.36	177.13	174.97	0.090	0.115	0.004
99	A	7.80	120.70	58.06	51.89	40.32	19.69	174.97	177.64	0.972	0.106	0.008
100	F	9.57	123.23	51.84	56.87	19.67	43.25	177.64	173.62	0.033	0.032	0.004
101	I	10.05	121.69	56.88	60.91	43.33	39.99	173.61	176.62	1.000	0.401	0.008
102	R	8.91	129.93	60.93	55.52	39.92	31.83	176.62	175.37	0.986	0.860	0.008
103	S	8.48	121.45	55.53	57.16	31.79	64.92	175.37	172.44	0.553	0.987	0.008
104	D	9.71	127.08	56.70	---	64.90	---	---	175.61	0.040	0.111	0.001
105	S	8.44	116.99	53.64	56.30	43.73	63.22	175.79	174.81	0.891	0.743	0.008
106	G	8.89	118.62	56.30	44.83	63.29	---	174.81	---	0.214	0.244	0.007
107	P											
108	T	7.44	108.58	63.68	59.83	32.91	72.42	176.36	173.55	0.669	0.873	0.008
109	T	9.58	119.14	59.85	62.29	72.55	---	173.55	173.12	1.000	0.043	0.007
110	S	8.65	120.17	62.31	57.22	72.65	67.01	173.11	170.97	1.000	0.173	0.008
111	F	9.65	119.20	57.23	56.30	67.11	42.47	170.97	173.30	0.931	0.185	0.008
112	E	8.99	126.02	56.25	53.37	42.43	32.01	173.32	176.48	0.911	0.935	0.008
113	S	8.76	120.85	53.32	58.50	31.96	62.38	176.47	174.88	0.483	0.921	0.008
114	A	8.15	128.44	58.55	53.54	62.42	19.12	174.87	178.75	1.000	0.676	0.008
115	A	7.01	116.02	53.59	52.61	19.02	19.89	178.75	176.76	1.000	0.491	0.008
116	C	7.69	118.02	52.60	49.89	19.92	46.19	176.76	168.88	0.754	0.000	0.008
117	P											
118	G	10.11	112.99	64.18	44.58	31.35	---	178.74	173.07	0.999	0.094	0.007

119	W	8.04	119.75	44.59	56.54	---	28.40	173.07	174.63	0.349	0.556	0.002
120	F	9.29	121.52	56.59	56.27	28.36	41.91	174.63	176.93	0.320	0.353	0.004
121	L	8.82	124.31	56.21	56.60	41.95	44.28	176.91	---	0.984	0.674	0.008
122	A	8.77	126.59	56.72	51.06	44.34	25.69	176.54	175.73	0.988	0.706	0.008
123	T	9.25	109.78	51.08	57.95	25.63	70.56	175.73	174.18	0.980	0.300	0.008
124	A	9.32	122.63	57.96	50.54	70.60	19.70	174.18	177.79	1.000	0.265	0.008
125	M	8.46	117.22	50.55	57.18	19.67	31.70	177.79	178.86	0.996	0.569	0.008
126	E	7.82	118.75	57.21	56.09	31.59	30.56	178.86	175.51	0.310	0.470	0.008
127	A	8.75	123.35	56.09	53.12	30.48	20.43	175.51	176.51	0.878	0.846	0.008
128	D	8.59	114.63	53.14	56.34	20.36	38.73	176.51	173.94	0.998	0.838	0.008
129	Q	8.45	116.58	56.34	51.96	38.70	28.36	173.93	173.46	0.838	0.347	0.008
130	P											
131	V	8.12	127.22	63.05	64.01	31.98	31.49	178.48	175.60	0.467	0.181	0.008
132	S	9.15	123.27	64.12	56.68	31.28	65.73	175.59	172.63	0.448	0.589	0.008
133	L	8.28	117.06	56.68	53.23	65.77	48.05	172.62	177.18	1.000	0.172	0.008
134	T	9.34	113.89	53.25	60.30	48.04	69.94	177.18	171.29	0.999	0.008	0.008
135	N	7.28	126.69	60.29	51.01	70.00	35.45	171.29	175.16	0.984	0.000	0.008
136	M	8.09	119.23	51.04	52.81	35.42	32.94	175.16	174.21	0.111	0.051	0.008
137	P											
138	D	8.38	117.52	64.42	54.72	31.76	40.89	176.98	176.28	0.640	0.555	0.008
139	E	7.45	118.55	54.72	57.05	40.86	30.39	176.28	176.97	0.125	0.298	0.008
140	G	8.10	106.80	57.08	45.73	30.37	---	176.97	174.22	0.898	0.481	0.007
141	V	7.63	116.03	45.76	63.04	---	32.57	174.22	175.48	0.572	0.850	0.002
142	M	7.90	117.46	63.05	53.74	32.50	35.89	175.48	175.01	0.526	0.693	0.008
143	V	9.61	125.01	53.74	62.67	35.87	31.03	175.01	176.11	0.753	0.241	0.008
144	T	8.27	111.42	62.72	60.26	30.97	69.57	176.11	174.20	0.062	0.093	0.008
145	K	6.59	122.74	60.24	55.50	69.68	35.50	174.20	173.22	0.999	0.514	0.008
146	F	8.80	117.68	55.51	56.25	35.46	42.29	173.22	175.13	0.818	0.264	0.008
147	Y	9.37	121.43	56.32	58.24	42.26	37.81	175.92	174.21	0.057	0.029	0.008
148	F	8.62	126.88	58.27	55.84	37.79	42.20	174.20	174.60	0.273	0.083	0.008
149	Q	8.66	124.96	55.86	54.82	42.20	32.94	174.60	174.01	0.166	0.303	0.008
150	E	9.11	128.51	54.39	58.13	32.96	29.24	173.76	176.14	0.225	0.125	0.008
151	D	8.41	125.62	58.14	54.06	29.21	42.44	176.14	174.42	0.292	0.050	0.008
152	E	7.66	126.84	54.09	57.79	42.43	31.58	174.42	180.69	0.975	0.050	0.008

Assigned amide groups that match the reference assignment are indicated by green shading. Missing amide groups that are present in the reference assignments are indicated by white shading. Amide groups that were assigned to residues that are not assigned in the reference assignments are indicated by orange shading. Amide groups that were assigned by BARASA and confirmed manually but were not in the reference assignments indicated by blue shading and are considered matching reference assignments. Residues that were not assigned by either BARASA or in the reference assignments were considered matching. Non-amide resonances were not considered when determining assignment accuracy.
The table is included in Excel format in the Source Data file.

Supplementary Table 5 – Assignments of IGPS by BARASA											
**Index**	**AA**	**H**	**N**	**CA(i-1)**	**CA**	**CB(i-1)**	**CB**	**CO(i-1)**	**Posterior**	**Likelihood**	**Prior**
1	G	10.34	111.75	---	---	---	---	---	0.713	0.030	0.004
2	P										
3	R										
4	Y										
5	L										
6	K	8.29	119.61	54.31	55.46	43.49	---	175.79	0.785	0.661	0.004
7	G										
8	W										
9	L	6.50	122.39	59.86	56.19	26.42	41.67	175.94	0.998	0.083	0.002
10	K	6.73	117.19	56.21	58.89	41.71	31.88	177.96	0.917	0.445	0.005
11	D	7.17	117.84	58.96	57.34	31.87	41.86	178.56	0.588	0.927	0.005
12	V	8.38	117.64	57.33	65.92	41.87	31.18	178.13	1.000	0.977	0.005
13	V	7.69	122.17	65.91	67.92	31.24	---	---	0.999	0.620	0.004
14	Q										
15	L	8.27	120.23	59.24	58.20	28.10	41.58	179.36	0.304	0.877	0.005
16	S	8.72	119.25	58.16	63.33	41.67	---	181.23	0.517	0.129	0.002
17	L	8.15	120.46	63.37	58.17	---	42.33	175.73	0.345	0.675	0.001
18	R	7.28	115.99	58.14	55.35	42.33	30.76	---	0.882	0.994	0.005
19	R	7.36	124.10	55.34	---	30.82	31.84	---	0.597	0.439	0.002
20	P										
21	S	8.14	115.98	63.02	58.12	32.60	64.94	176.38	0.900	0.961	0.002
22	F	9.07	126.94	58.09	57.52	64.96	41.88	174.62	0.313	0.155	0.002
23	R										
24	A	7.35	125.45	55.95	51.64	29.88	21.71	---	1.000	0.515	0.005
25	S										
26	R										
27	Q										
28	R										
29	P										
30	I	8.23	120.97	62.74	61.11	32.19	38.11	175.83	0.903	0.886	0.005
31	I	9.20	133.11	61.04	60.32	38.19	---	176.08	0.566	0.095	0.004
32	S	7.92	115.77	60.32	59.15	38.53	62.81	174.60	0.906	0.127	0.005
33	L	7.93	130.11	59.18	57.35	62.83	41.80	---	0.998	0.983	0.002
34	N	8.34	115.11	57.35	57.42	41.78	34.45	176.80	0.078	0.069	0.001
35	E	7.44	117.24	57.42	59.31	34.46	29.29	176.15	0.650	0.168	0.005
36	R										
37	I										
38	L	7.24	118.66	66.33	58.32	38.04	41.42	---	0.783	0.936	0.005

39	E	7.99	121.05	58.30	59.77	41.39	29.38	179.61	0.153	0.872	0.005
40	F	8.87	124.79	59.78	61.69	29.41	36.72	179.76	0.333	0.325	0.005
41	N	9.15	117.91	61.72	56.64	36.72	39.34	178.96	0.720	0.297	0.005
42	K	8.27	121.32	56.61	59.43	39.35	32.35	179.15	0.604	0.577	0.005
43	S	7.66	113.60	59.41	58.75	32.37	64.23	177.95	0.993	0.960	0.005
44	N	8.02	116.97	58.73	54.46	64.19	37.42	172.86	0.906	0.941	0.005
45	I	8.12	119.44	54.45	60.15	37.50	39.52	173.90	1.000	0.998	0.005
46	T	8.85	127.56	60.15	65.67	39.62	68.74	177.42	1.000	0.408	0.005
47	A	9.29	131.80	65.69	51.69	68.77	18.76	172.97	1.000	0.552	0.005
48	I	9.37	125.30	51.71	58.86	18.78	39.38	174.32	1.000	0.636	0.005
49	I	9.65	129.96	58.92	59.55	39.33	37.95	175.81	0.998	0.600	0.005
50	A	7.64	130.12	59.70	51.45	37.97	20.16	174.26	0.995	0.947	0.005
51	E	8.86	124.26	51.48	54.42	20.12	33.69	176.35	0.491	0.958	0.005
52	Y	9.30	128.47	54.46	56.90	33.74	40.38	175.56	0.989	0.859	0.005
53	K	6.88	125.85	56.91	54.46	40.30	34.10	173.83	0.994	0.781	0.005
54	R	8.88	121.55	54.46	57.11	34.12	31.79	172.59	0.989	0.087	0.005
55	K	7.36	115.13	57.14	53.24	31.86	37.75	175.49	0.949	0.521	0.005
56	S	7.50	111.03	53.22	55.49	37.69	65.48	173.82	1.000	0.318	0.005
57	P										
58	S										
59	G	7.63	108.54	59.02	45.27	63.39	---	174.41	0.790	0.942	0.004
60	L	7.18	123.31	45.26	56.01	---	42.93	174.04	1.000	0.996	0.002
61	D	7.90	125.49	56.05	54.51	43.00	41.45	176.46	0.981	0.835	0.005
62	V	8.50	122.89	54.58	61.11	41.17	35.42	174.77	0.896	0.859	0.005
63	E	8.17	125.07	61.03	55.62	35.44	30.25	173.48	0.999	0.776	0.005
64	R	7.30	122.68	55.54	54.88	30.36	33.08	175.53	0.711	0.638	0.005
65	D										
66	P										
67	I	7.86	121.46	64.85	63.08	31.50	35.18	178.95	0.995	0.327	0.005
68	E	8.58	121.94	63.07	59.85	35.26	30.45	177.62	0.829	0.111	0.005
69	Y	8.08	117.96	59.83	61.90	30.56	39.87	180.06	0.300	0.360	0.005
70	S	7.84	114.98	61.84	63.80	39.93	62.78	176.99	1.000	0.697	0.005
71	K	8.73	120.18	63.82	58.50	62.78	31.13	175.65	0.990	0.731	0.005
72	F	7.74	120.51	58.52	61.31	31.17	39.35	179.27	0.459	0.873	0.005
73	M	8.15	113.55	61.34	56.06	39.31	32.23	177.43	0.999	0.595	0.005
74	E	8.18	121.34	56.04	59.33	32.23	29.84	178.88	0.508	0.585	0.005
75	R	6.88	113.96	59.32	57.21	30.05	---	176.17	0.273	0.271	0.004
76	Y	7.15	113.57	57.21	58.56	30.26	44.96	175.85	0.913	0.000	0.005
77	A	8.66	120.82	58.64	52.29	45.07	20.39	175.64	0.999	0.000	0.005
78	V	8.75	121.92	52.28	62.00	20.35	32.28	176.48	1.000	0.187	0.005

79	G	7.35	104.85	62.05	44.83	32.30	---	174.83	1.000	0.706	0.004
80	L	8.90	119.18	44.81	52.99	---	46.55	---	1.000	0.844	0.002
81	S	8.74	116.66	53.00	55.43	46.60	63.30	175.40	1.000	0.413	0.005
82	I	8.78	126.85	55.44	58.52	63.35	38.92	173.94	0.987	0.421	0.005
83	L	7.39	130.52	58.54	52.75	38.85	42.13	175.05	0.439	0.105	0.005
84	T	8.59	109.47	52.76	61.21	42.11	67.99	179.77	0.099	0.000	0.005
85	E	6.49	126.61	61.16	58.32	67.82	30.90	173.87	1.000	0.002	0.005
86	E	8.27	126.28	58.32	59.62	30.95	33.30	176.78	0.186	0.044	0.005
87	K										
88	Y										
89	F	7.59	114.25	59.51	56.83	39.65	---	177.10	0.680	0.898	0.004
90	N	7.16	116.01	56.77	54.18	39.82	38.44	175.70	0.894	0.330	0.005
91	G	8.85	105.36	54.14	44.26	38.47	---	174.62	0.995	0.930	0.004
92	S	7.12	105.14	44.25	57.70	---	65.80	172.14	1.000	0.010	0.002
93	Y										
94	E	8.87	124.78	59.78	59.79	---	---	179.76	0.018	0.105	0.001
95	T	7.80	116.02	59.79	67.13	29.16	68.51	178.71	1.000	0.999	0.005
96	L	6.99	120.34	67.16	59.81	68.55	41.05	175.46	1.000	0.415	0.005
97	R	8.31	118.98	59.77	59.20	41.06	30.44	176.86	0.391	0.553	0.005
98	K	8.07	118.24	59.16	59.60	30.39	32.62	179.17	0.124	1.000	0.002
99	I	7.99	121.07	59.59	66.18	32.67	38.49	180.20	0.916	0.955	0.005
100	A	9.03	121.87	66.20	55.09	38.53	17.77	178.42	0.544	0.709	0.005
101	S	7.49	109.12	55.11	59.91	17.85	63.98	178.70	1.000	0.976	0.005
102	S	7.92	114.78	59.96	---	63.97	65.68	174.62	1.000	0.919	0.005
103	V	7.38	112.36	---	59.71	65.69	35.80	173.24	0.476	0.681	0.001
104	S	9.11	117.61	59.71	57.64	35.81	64.09	176.23	0.929	0.822	0.005
105	I	6.40	112.36	57.64	58.73	64.14	38.02	173.80	0.988	0.621	0.005
106	P										
107	I	10.82	122.92	62.91	59.48	33.49	42.02	176.17	1.000	0.000	0.005
108	L	9.53	129.77	59.45	52.97	42.01	44.47	175.49	0.998	0.693	0.005
109	M	8.39	127.80	52.99	56.61	44.50	34.94	174.62	0.997	0.350	0.005
110	K	8.29	132.37	56.49	55.93	34.84	33.08	172.59	0.537	0.008	0.002
111	D	7.23	125.89	55.93	51.44	33.14	43.48	171.83	0.999	0.009	0.005
112	F	11.68	127.34	51.46	57.31	43.44	38.31	174.03	0.000	0.000	0.005
113	I	9.02	125.15	57.40	62.33	38.31	36.89	174.18	0.995	0.470	0.005
114	V	9.20	117.03	62.32	59.74	36.87	34.00	174.76	0.999	0.119	0.005
115	K	8.02	118.03	59.75	55.07	34.06	35.85	174.75	0.937	0.439	0.005
116	E	9.37	122.57	55.06	60.89	35.87	28.85	175.65	0.954	0.458	0.005
117	S	8.59	113.22	60.95	61.91	28.73	---	179.32	0.634	0.880	0.004
118	Q	7.40	119.24	61.89	59.12	---	32.96	176.30	0.002	0.003	0.002

119	I	7.62	119.55	59.24	65.29	33.05	36.70	178.53	0.003	0.003	0.005
120	D	7.79	120.37	65.29	58.50	36.68	39.82	177.30	0.533	0.879	0.005
121	D	8.50	119.03	58.30	---	39.81	---	177.75	0.123	0.930	0.004
122	A	7.50	120.27	58.07	55.68	39.79	20.13	176.61	0.985	0.586	0.005
123	Y	8.22	116.54	55.71	61.53	20.09	37.98	178.75	1.000	0.810	0.005
124	N	8.93	118.85	61.54	55.70	38.15	---	179.00	0.223	0.579	0.004
125	L	8.30	116.39	55.70	56.21	38.27	44.01	176.06	0.500	0.317	0.002
126	G	7.02	100.70	56.30	44.58	---	---	---	0.831	0.263	0.002
127	A	7.94	119.05	44.55	52.54	---	19.89	173.36	0.659	0.740	0.002
128	D	9.49	121.71	52.54	56.69	19.90	44.94	176.50	1.000	0.295	0.005
129	T	8.15	109.77	56.62	59.98	44.84	67.11	176.28	0.973	0.002	0.005
130	V	8.10	112.22	59.97	58.39	67.17	36.02	172.55	0.261	0.007	0.005
131	L	6.81	122.46	58.35	53.31	36.06	47.14	172.78	0.950	0.013	0.005
132	L	9.03	127.76	53.48	---	47.14	44.35	176.81	0.999	0.015	0.005
133	I	9.12	124.99	53.77	61.45	44.35	39.95	174.71	0.401	0.481	0.005
134	V	7.59	125.24	61.46	67.35	39.94	31.82	175.48	1.000	0.119	0.005
135	K	8.17	113.17	67.41	58.91	31.69	33.45	177.49	1.000	0.255	0.005
136	I	6.77	106.94	58.98	61.04	33.48	37.55	177.77	0.856	0.094	0.005
137	L	7.41	121.81	60.98	53.46	37.59	47.47	173.39	0.639	0.012	0.005
138	T	8.22	109.57	53.46	61.06	47.54	71.29	177.03	0.995	0.037	0.005
139	E										
140	R	8.10	116.22	61.05	58.66	29.98	---	177.78	0.234	0.671	0.004
141	E	7.57	119.76	58.69	59.11	30.30	31.24	179.66	0.044	0.526	0.002
142	L	8.58	119.91	59.17	58.62	31.30	41.40	178.60	0.294	0.660	0.005
143	E	8.28	117.71	58.60	60.46	41.41	30.27	---	0.179	0.787	0.002
144	S	7.86	113.59	60.49	61.82	30.31	63.03	178.54	0.490	0.775	0.005
145	L	8.69	123.17	61.92	58.28	63.06	41.77	177.86	0.523	0.811	0.005
146	L	8.64	121.79	58.24	59.08	41.83	42.43	178.38	0.678	0.704	0.005
147	E	7.88	117.73	59.12	59.22	42.56	29.44	178.42	0.088	0.790	0.002
148	Y	8.52	122.57	59.22	62.63	29.33	39.08	179.23	0.357	0.882	0.005
149	A	8.81	121.70	62.60	56.04	39.09	17.68	179.54	0.968	0.701	0.005
150	R										
151	S	8.18	120.35	59.84	61.92	30.28	---	181.36	0.050	0.056	0.004
152	Y	7.27	120.03	62.04	59.69	55.43	40.45	175.60	0.811	0.000	0.005
153	G	8.07	105.53	59.67	44.93	40.45	---	176.12	0.756	0.160	0.004
154	M	7.58	117.90	44.91	55.39	---	35.25	177.47	1.000	0.022	0.002
155	E	7.76	121.32	55.35	52.43	35.26	30.15	174.45	1.000	0.570	0.005
156	P										
157	L	7.91	122.98	62.82	53.54	33.10	42.48	173.53	0.980	0.543	0.005
158	I	8.58	128.73	53.51	59.83	42.53	36.93	174.61	0.718	0.579	0.005

159	E	9.48	130.27	59.79	56.08	36.92	32.45	175.31	0.935	0.431	0.005
160	I	9.37	121.77	56.01	59.08	32.57	42.55	---	0.993	0.696	0.005
161	N	10.41	120.65	59.01	53.80	42.58	---	173.92	0.924	0.008	0.004
162	D	7.55	115.75	53.77	53.23	44.21	42.47	173.26	1.000	0.060	0.005
163	E	9.07	117.26	53.21	60.07	42.44	29.74	175.84	1.000	0.892	0.005
164	N	8.07	121.36	60.00	56.69	29.69	38.39	178.22	0.501	0.855	0.005
165	D	8.77	120.01	56.76	---	38.43	41.98	177.57	0.605	0.871	0.005
166	L	7.80	118.45	56.96	58.38	41.94	41.43	178.40	0.331	0.829	0.005
167	D	7.76	118.43	58.36	57.95	41.49	---	176.55	0.033	0.202	0.002
168	I	7.45	117.80	57.86	65.62	41.54	38.39	178.92	0.523	0.909	0.002
169	A	8.38	119.82	65.62	55.51	38.44	18.23	177.04	0.503	0.978	0.005
170	L	8.60	114.06	55.52	57.35	18.28	40.54	178.96	1.000	0.414	0.005
171	R	8.02	122.39	57.37	60.15	40.58	30.29	181.07	0.495	0.841	0.005
172	I	7.95	111.06	60.17	61.42	30.28	38.21	179.01	0.968	0.834	0.005
173	G	7.48	106.80	61.42	46.25	38.28	---	176.16	0.995	0.989	0.004
174	A	7.95	122.01	46.22	53.86	---	20.22	175.36	0.693	0.756	0.002
175	R	7.50	117.25	53.83	56.02	20.23	32.09	176.30	0.989	0.116	0.005
176	F	8.64	128.99	56.02	53.76	32.18	39.76	175.56	0.001	0.001	0.005
177	I	8.83	125.76	53.73	59.76	39.86	42.84	173.75	0.411	0.044	0.005
178	G	8.94	115.94	59.76	43.37	42.82	---	173.93	0.980	0.718	0.004
179	I	9.16	124.07	43.30	61.47	---	38.94	171.74	1.000	0.773	0.002
180	N	8.73	128.80	61.54	52.62	38.72	---	175.51	0.389	0.578	0.004
181	S	8.67	120.93	52.65	59.40	38.65	62.96	176.25	1.000	0.817	0.005
182	R	8.36	125.80	59.60	55.00	62.95	31.86	173.52	0.518	0.592	0.005
183	D	8.29	126.82	54.91	53.76	31.94	42.55	174.57	0.391	0.822	0.005
184	L	8.75	127.91	53.69	58.04	42.67	43.76	177.98	0.993	0.245	0.005
185	E	8.30	113.81	58.09	59.60	43.82	30.79	176.67	0.311	0.076	0.005
186	T	7.86	106.80	59.51	62.37	30.65	70.92	178.70	1.000	0.853	0.005
187	L	8.43	115.61	62.32	56.81	70.93	38.39	175.65	1.000	0.875	0.005
188	E	7.76	120.46	56.77	---	38.51	31.25	176.02	0.648	0.803	0.005
189	I	8.61	124.51	56.62	60.29	31.30	39.28	176.52	0.778	0.953	0.005
190	N	8.78	125.49	60.30	52.52	39.36	---	175.98	0.472	0.997	0.004
191	K	8.71	121.10	52.56	---	38.64	---	176.32	0.285	0.361	0.004
192	E	9.92	123.10	59.71	60.10	33.58	---	178.54	0.150	0.001	0.002
193	N	8.01	118.83	60.11	55.62	29.04	38.27	179.42	0.401	0.836	0.005
194	Q	7.04	118.21	55.66	59.18	38.31	29.97	176.65	0.335	0.682	0.005
195	R	7.88	117.56	59.19	---	30.00	29.44	177.20	0.165	0.712	0.005
196	K										
197	L	7.87	118.44	59.52	57.77	32.12	42.38	179.38	0.436	0.944	0.005
198	I	7.99	116.68	57.77	66.31	42.37	37.95	179.51	0.773	0.873	0.005

199	S	7.42	112.88	66.35	60.86	37.99	63.65	178.19	1.000	0.981	0.005
200	M	7.49	119.27	60.78	56.23	63.66	34.59	174.32	0.949	0.772	0.005
201	I	7.14	120.96	56.22	59.52	34.51	39.60	175.48	0.979	0.464	0.005
202	P										
203	S										
204	N										
205	V										
206	V	7.92	127.76	61.77	63.95	33.49	31.74	175.47	1.000	0.393	0.005
207	K	9.22	128.20	63.96	54.87	31.69	36.15	174.54	0.997	0.675	0.005
208	V	9.16	123.04	54.85	59.30	35.97	34.78	175.63	0.995	0.666	0.005
209	A	8.00	129.35	59.25	51.91	34.59	20.76	175.23	0.945	0.320	0.005
210	E	7.85	121.63	51.93	55.94	20.72	33.91	174.78	0.778	0.363	0.005
211	S										
212	G	9.93	111.80	62.67	46.62	61.23	---	175.96	1.000	0.000	0.004
213	I	8.86	123.61	46.60	62.98	---	37.03	175.08	0.999	0.086	0.002
214	S										
215	E	7.99	119.55	58.84	54.61	66.14	33.47	172.91	0.906	0.533	0.005
216	R										
217	N										
218	E	7.19	119.00	56.81	59.15	37.73	29.97	177.08	0.388	0.893	0.005
219	I	6.75	119.27	59.15	64.42	30.02	37.32	178.35	0.411	0.780	0.005
220	E	7.87	117.80	64.44	59.29	37.47	29.70	177.20	0.599	0.914	0.005
221	E										
222	L	8.07	118.39	59.48	57.82	29.94	43.22	179.55	0.319	0.650	0.005
223	R	9.10	122.39	57.79	59.67	43.23	29.62	180.40	0.339	0.248	0.005
224	K	7.62	119.48	59.60	---	29.62	32.31	180.83	0.377	0.927	0.005
225	L	7.31	117.07	59.22	55.03	32.32	42.87	177.92	0.789	0.855	0.005
226	G	7.81	104.97	55.00	45.11	42.86	---	176.98	0.528	0.987	0.004
227	V	7.38	118.72	45.09	64.92	---	31.49	174.71	1.000	0.822	0.002
228	N	8.02	122.42	64.93	57.25	31.60	---	174.62	0.834	0.234	0.004
229	A	7.79	118.88	57.26	50.88	40.83	23.26	175.49	0.990	0.214	0.005
230	F	8.08	116.55	50.88	57.02	23.29	42.41	173.34	1.000	0.961	0.005
231	L	8.94	129.75	57.02	55.19	42.38	44.30	173.01	0.978	0.364	0.005
232	I	8.26	125.91	55.18	61.79	44.35	41.48	175.30	0.931	0.277	0.005
233	G	10.14	116.58	61.85	46.93	41.44	---	173.12	0.367	0.000	0.004
234	S	11.19	126.69	46.99	64.70	---	---	173.57	0.797	0.000	0.004
235	S	9.20	114.42	64.77	62.31	63.16	---	175.46	1.000	0.295	0.004
236	L	6.86	119.98	62.32	56.00	63.10	42.15	176.81	0.830	0.713	0.005
237	M	8.46	116.48	55.83	---	42.09	29.80	177.72	0.180	0.291	0.005
238	R	7.82	115.92	55.62	59.13	30.10	30.81	180.04	0.469	0.150	0.005

239	N	7.26	112.86	59.12	51.29	30.87	39.44	177.06	1.000	0.487	0.005
240	P										
241	E	7.96	111.53	65.77	58.71	32.23	28.79	177.38	0.621	0.155	0.005
242	K	8.17	122.24	58.73	58.19	28.90	32.08	178.63	0.086	0.667	0.002
243	I	7.66	117.80	58.13	63.92	32.09	37.81	177.31	0.744	0.986	0.005
244	K	7.01	118.42	63.91	59.25	37.91	32.03	175.34	0.996	0.668	0.005
245	E	7.00	116.48	59.25	57.60	32.01	29.89	178.22	0.612	0.879	0.005
246	F	7.47	116.07	57.60	56.50	30.01	38.39	178.37	0.108	0.072	0.005
247	I	7.14	110.88	56.47	62.20	38.42	39.21	175.83	0.999	0.005	0.005
248	L	7.26	128.03	62.19	57.54	39.31	42.78	175.55	0.992	0.011	0.005

Assigned amide groups that match the reference assignment are indicated by green shading. Missing amide groups that are present in the reference assignments are indicated by white shading. Amide groups that were assigned to residues that are not assigned in the reference assignments are indicated by orange shading. Residues that were not assigned by either BARASA or in the reference assignments were considered matching. Non-amide resonances were not considered when determining assignment accuracy.

The table is included in Excel format in the Source Data file.

Supplementary Table 6 – Assignments of MBP by BARASA

Index	AA	H	N	CA(i-1)	CA	CB(i-1)	CB	CO(i-1)	CO	Posterior	Likelihood	Prior
1	M	10.51	134.87	---	---	---	---	---	---	0.912	0.003	0.003
2	K											
3	T	8.04	116.55	55.82	61.61	32.19	69.10	176.36	174.30	0.572	1.000	0.003
4	E	8.36	122.87	61.62	55.68	69.13	30.27	174.30	175.96	0.602	0.726	0.003
5	E	8.31	122.44	55.68	56.22	30.27	29.48	175.98	176.84	0.210	0.261	0.003
6	G	8.41	110.60	56.22	45.23	29.48	---	176.85	172.17	0.759	0.836	0.003
7	K	7.54	119.49	45.24	54.47	---	34.43	172.18	173.43	0.262	0.399	0.001
8	L	8.22	118.82	54.47	52.86	34.43	45.54	173.44	175.22	0.983	0.545	0.003
9	V	9.85	125.74	52.85	61.25	45.54	32.81	175.23	175.82	0.878	0.007	0.003
10	I	8.95	128.63	61.25	59.46	32.81	40.29	175.83	174.59	0.928	0.629	0.003
11	W	8.88	126.97	59.46	53.69	40.30	32.13	174.60	174.00	0.802	0.725	0.003
12	I	8.54	122.23	53.68	59.38	32.14	40.63	174.01	171.04	0.355	0.122	0.003
13	N	8.69	122.76	59.38	53.88	40.64	39.23	171.05	177.29	0.904	0.381	0.003
14	G	8.06	106.61	53.87	45.90	39.23	---	177.31	173.09	0.497	0.725	0.003
15	D	7.75	116.97	45.89	52.56	---	39.12	173.10	176.02	0.630	0.932	0.002
16	K	7.39	118.88	52.51	---	39.12	32.31	176.03	177.41	0.327	0.431	0.003
17	G	8.59	108.20	52.46	47.60	32.31	---	177.42	175.61	0.957	0.201	0.003
18	Y	8.03	120.72	47.59	59.22	---	36.99	175.62	177.06	0.537	0.873	0.002
19	N	7.93	123.71	59.22	55.69	36.94	---	177.07	178.93	0.024	0.038	0.003
20	G	8.77	111.83	55.70	46.94	36.86	---	178.95	175.85	0.736	0.096	0.003
21	L	8.14	122.02	46.93	57.10	---	40.60	175.87	178.65	0.338	1.000	0.002
22	A	7.91	121.27	57.10	54.61	40.60	16.80	178.67	180.56	0.497	0.999	0.003
23	E	7.63	120.64	54.60	59.22	16.80	27.92	180.56	180.07	0.542	0.920	0.003
24	V	7.65	123.31	59.22	66.23	27.93	30.45	180.08	179.40	0.877	0.383	0.003
25	G	8.53	106.87	66.24	47.12	30.45	---	179.41	175.05	1.000	0.470	0.003
26	K	8.11	122.93	47.10	58.79	---	31.11	175.06	179.42	0.896	0.773	0.002
27	K	7.48	122.95	58.78	59.20	31.19	---	179.44	178.03	0.229	0.799	0.003
28	F	7.90	120.32	59.20	60.90	31.30	38.59	178.06	178.89	0.485	0.565	0.003
29	E	8.73	124.33	60.89	58.56	38.59	29.10	178.90	179.79	0.323	0.142	0.003
30	K	7.92	122.16	58.67	---	29.13	30.66	179.79	178.25	0.087	0.251	0.003
31	D	7.40	116.58	58.79	56.00	30.65	40.35	178.25	---	0.082	0.345	0.003
32	T	7.75	107.40	55.98	62.26	40.35	71.66	178.21	176.11	0.282	0.125	0.003
33	G	8.42	112.88	62.25	44.89	71.66	---	176.14	173.39	0.954	0.378	0.003
34	I	7.57	124.44	44.88	58.31	---	35.06	173.40	173.62	0.922	0.115	0.002
35	K	7.54	124.82	58.31	56.03	35.07	32.12	173.67	175.56	0.062	0.059	0.003
36	V	8.29	124.99	56.02	60.54	32.13	---	175.61	---	0.190	0.540	0.003
37	T	8.95	125.29	60.54	60.89	32.19	70.40	175.68	172.53	0.037	0.036	0.003
38	V	8.70	127.56	60.93	---	70.39	31.47	172.55	175.23	0.960	0.920	0.003

39	E	9.46	127.13	60.94	53.76	31.48	32.48	175.24	173.36	0.779	0.913	0.003
40	H	8.16	115.41	53.75	50.79	32.48	27.96	173.37	172.42	0.777	0.877	0.003
41	P											
42	D	8.04	121.37	61.75	54.30	30.08	40.44	176.61	176.38	0.514	0.999	0.003
43	K	8.59	119.53	54.30	55.97	40.43	28.74	176.34	178.30	0.692	0.983	0.003
44	L	7.25	120.21	55.97	58.98	28.74	40.09	178.31	175.84	0.398	0.225	0.003
45	E	10.22	123.96	58.98	56.56	40.10	24.11	175.86	178.42	0.507	0.002	0.003
46	E	7.16	120.86	56.55	56.06	24.11	29.35	178.44	178.15	0.572	0.214	0.003
47	K	8.04	120.39	56.05	57.94	29.35	32.71	178.15	179.00	0.390	0.740	0.003
48	F	8.40	117.30	57.94	62.95	32.70	35.51	179.00	172.18	0.113	0.068	0.003
49	P											
50	Q	6.77	114.85	64.99	57.40	29.47	27.94	177.73	178.23	0.824	0.683	0.003
51	V	7.41	110.47	57.38	61.29	27.94	31.50	178.15	---	0.001	0.000	0.003
52	A	7.91	125.12	61.28	54.47	31.51	17.12	178.04	179.76	0.108	0.029	0.003
53	A	7.54	117.78	54.47	52.63	17.12	17.56	179.76	178.35	0.297	0.924	0.003
54	T	7.09	106.55	52.61	61.07	17.57	69.45	178.37	175.17	1.000	0.832	0.003
55	G	7.68	109.23	61.06	45.04	69.46	---	175.19	173.43	0.503	0.949	0.003
56	D	7.40	119.44	45.03	52.95	---	41.65	173.45	175.11	0.894	0.947	0.002
57	G	7.99	106.70	52.95	43.51	41.65	---	175.13	170.25	0.812	0.627	0.003
58	P											
59	D	8.55	118.10	61.28	57.72	30.00	43.11	174.86	175.44	0.975	0.706	0.003
60	I	7.46	114.70	57.72	58.43	43.11	41.93	175.48	173.59	0.997	0.880	0.003
61	I	8.72	124.58	58.42	57.47	41.93	40.70	173.58	172.75	0.885	0.704	0.003
62	F	8.47	126.25	57.46	55.22	40.70	41.45	172.77	176.51	0.909	0.351	0.003
63	W	9.01	121.51	55.21	56.80	41.46	31.06	176.52	173.47	0.485	0.674	0.003
64	A	6.22	126.39	56.80	53.32	31.06	18.15	173.47	179.57	1.000	0.569	0.003
65	H	7.95	116.40	53.31	59.73	18.15	29.53	179.58	178.24	0.124	0.321	0.003
66	D	7.55	119.46	59.72	56.11	29.53	38.44	178.24	177.59	0.276	0.845	0.003
67	R	6.85	117.35	56.10	54.32	38.43	29.23	177.62	177.32	0.627	0.504	0.003
68	F	7.48	116.76	54.32	57.62	29.22	35.90	177.33	177.78	0.171	0.151	0.003
69	G	7.18	107.63	57.61	47.05	35.90	---	177.80	175.65	0.980	0.230	0.003
70	G	7.57	107.84	47.03	46.37	---	---	175.66	177.12	1.000	0.968	0.003
71	Y	6.72	119.28	46.36	56.53	---	35.17	177.14	178.22	0.903	0.045	0.002
72	A	8.42	122.99	56.52	53.76	35.17	17.31	178.25	181.95	0.422	0.006	0.003
73	Q	8.46	122.72	53.75	58.03	17.31	26.98	181.96	177.60	0.087	0.010	0.003
74	S	7.15	112.22	58.02	58.55	27.00	63.52	177.60	173.57	0.766	0.857	0.003
75	G	7.87	109.55	58.55	45.82	63.51	---	173.58	175.97	0.910	0.718	0.003
76	L	7.67	114.01	45.81	55.08	---	41.91	175.99	177.53	0.982	0.865	0.002
77	L	7.26	116.25	55.07	52.04	41.92	42.81	177.54	176.53	0.929	0.988	0.003
78	A	8.87	126.23	52.04	50.47	42.81	18.30	176.54	176.26	1.000	0.974	0.003

79	E	8.02	121.10	50.45	55.78	18.30	28.74	176.29	176.54	0.998	0.960	0.003
80	I	8.01	123.00	55.78	59.12	28.76	38.37	176.60	175.50	0.519	0.872	0.003
81	T	8.58	112.93	59.12	57.32	38.37	68.70	175.51	172.64	0.665	0.874	0.003
82	P											
83	D	8.91	123.86	61.82	52.98	31.00	40.94	177.37	176.61	0.408	0.534	0.003
84	K	8.37	123.13	52.99	59.23	40.93	31.03	176.63	177.50	0.470	0.919	0.003
85	A	8.03	119.63	46.31	54.25	31.05	16.88	177.51	180.19	0.000	0.000	0.002
86	F	7.75	118.73	54.24	61.76	16.88	38.47	180.20	178.14	0.162	0.267	0.003
87	Q	8.33	117.36	61.76	59.14	38.48	27.64	178.15	179.90	0.103	0.119	0.003
88	D	7.90	117.13	59.14	55.54	27.63	40.28	179.91	176.33	0.312	0.641	0.003
89	K	7.63	117.40	55.54	57.61	40.28	31.29	176.35	176.61	0.355	0.715	0.003
90	L	7.49	120.21	57.60	52.91	31.29	41.89	176.59	---	0.569	0.342	0.003
91	Y	7.56	117.24	52.91	58.55	41.90	38.38	176.60	---	0.616	0.228	0.003
92	P											
93	F	7.86	112.93	66.27	58.74	30.84	36.09	179.42	176.19	0.965	0.251	0.003
94	T	6.94	111.24	58.73	64.18	36.09	66.53	176.21	---	0.390	0.279	0.003
95	W	6.50	120.04	64.18	57.98	66.53	28.77	176.26	178.14	0.535	0.137	0.003
96	D	7.09	115.73	57.98	56.70	28.76	39.31	178.17	---	0.232	0.581	0.003
97	A	6.83	118.80	56.69	53.11	39.30	17.13	178.23	176.68	0.754	0.666	0.003
98	V	6.81	106.30	53.10	59.12	17.13	29.79	176.69	172.52	1.000	0.087	0.003
99	R	6.87	121.41	59.12	54.29	29.80	30.95	172.53	176.45	0.139	0.059	0.003
100	Y	9.53	126.90	54.28	57.83	30.94	41.19	176.46	175.38	0.831	0.692	0.003
101	N	8.83	129.14	57.82	53.21	---	---	---	---	0.060	0.101	0.001
102	G	8.43	102.46	53.28	45.00	36.52	---	175.18	173.78	0.979	0.998	0.003
103	K	7.60	121.44	44.99	54.03	---	34.06	173.78	175.08	0.365	0.981	0.002
104	L	8.79	123.59	54.03	54.63	34.06	41.56	175.09	178.30	0.822	0.966	0.003
105	I	8.65	112.89	54.62	59.13	41.56	38.42	178.32	175.34	0.913	0.516	0.003
106	A	7.50	117.41	59.13	51.47	38.42	21.36	175.35	173.53	0.987	0.942	0.003
107	Y	8.80	113.90	51.46	55.44	21.36	39.32	173.54	174.22	0.997	0.748	0.003
108	P											
109	I	8.34	115.57	61.79	60.36	32.13	36.99	174.40	176.38	0.004	0.001	0.003
110	A	7.71	118.52	60.35	50.08	37.02	22.55	176.39	174.51	0.342	0.184	0.003
111	V	8.59	121.11	50.07	61.30	22.55	33.40	174.52	173.56	0.983	0.533	0.003
112	E	9.39	123.66	61.30	54.38	33.41	32.51	173.61	---	0.511	0.852	0.003
113	A	6.22	117.65	54.38	50.29	32.51	21.46	173.69	176.32	0.997	0.096	0.003
114	L	8.26	123.16	50.28	54.56	21.46	42.72	176.33	173.37	1.000	0.464	0.003
115	S	7.30	108.21	54.55	56.57	42.72	66.72	173.38	171.75	0.604	0.012	0.003
116	L	7.12	122.33	56.57	53.81	66.72	42.97	171.76	174.00	1.000	0.540	0.003
117	I	8.48	129.75	53.81	59.51	42.98	37.48	174.02	174.38	0.947	0.379	0.003
118	Y	8.90	121.99	59.51	54.00	37.49	41.61	174.41	172.72	0.779	0.735	0.003

119	N	9.14	121.36	54.00	51.55	41.61	37.09	172.73	175.53	0.638	0.830	0.003
120	K	8.63	125.68	51.55	58.15	37.09	31.97	175.55	177.45	0.637	0.892	0.003
121	D	7.99	115.80	58.16	55.80	31.97	39.79	177.43	---	0.246	0.868	0.003
122	L	6.95	117.98	55.79	54.85	39.78	43.36	177.42	176.51	0.755	0.632	0.003
123	L	8.16	120.06	54.85	51.16	43.36	---	176.52	171.12	0.419	0.035	0.003
124	P											
125	N	7.32	112.94	64.15	49.08	31.08	39.35	177.08	171.32	1.000	0.693	0.003
126	P											
127	P											
128	K	7.96	118.65	61.80	55.98	30.57	32.55	175.87	177.17	0.596	0.892	0.003
129	T	7.69	108.83	55.97	59.49	32.55	71.26	177.19	174.38	0.224	0.271	0.003
130	W	9.99	124.01	59.49	60.41	71.26	27.92	174.39	179.68	1.000	0.104	0.003
131	E	10.41	118.20	60.41	61.18	27.92	27.56	179.70	178.41	0.981	0.137	0.003
132	E	7.43	116.85	61.18	56.59	27.53	30.63	178.42	176.77	0.478	0.369	0.003
133	I	8.03	121.71	56.59	66.44	30.63	33.37	176.78	174.18	0.000	0.000	0.003
134	P											
135	A	7.64	118.31	66.52	54.67	29.84	17.28	177.48	181.03	0.908	0.939	0.003
136	L	7.44	120.21	54.64	56.83	17.30	41.29	181.04	179.04	0.634	0.969	0.003
137	D	8.65	118.67	56.83	58.43	41.27	---	179.06	177.13	0.136	0.477	0.003
138	K	7.86	117.51	58.43	59.79	41.24	31.37	177.16	179.62	0.278	0.405	0.003
139	E	7.30	118.72	59.79	58.65	31.37	28.74	179.59	---	0.072	0.146	0.003
140	L	8.23	121.45	58.64	57.40	28.76	39.65	179.54	180.46	0.068	0.074	0.003
141	K	9.08	124.29	57.39	58.31	39.64	31.26	180.48	181.40	0.016	0.005	0.003
142	A	7.09	122.22	58.31	53.82	31.26	17.14	181.40	178.40	0.842	0.301	0.003
143	K	7.47	115.97	53.82	54.45	17.14	31.71	178.42	176.52	0.568	0.998	0.003
144	G											
145	K	7.69	120.06	45.52	52.75	---	34.47	174.11	173.59	0.411	0.744	0.002
146	S	7.40	109.65	52.74	56.09	34.52	65.84	173.61	174.44	0.436	0.193	0.003
147	A	9.41	123.01	56.07	55.09	65.85	17.32	174.46	175.68	1.000	0.955	0.003
148	L	8.50	116.97	55.07	53.82	17.32	44.80	175.69	174.85	0.999	0.908	0.003
149	M	8.01	121.85	53.81	55.23	44.80	37.17	174.87	174.08	0.902	0.335	0.003
150	F	8.40	120.32	55.19	---	37.17	40.83	174.10	171.35	0.554	0.189	0.003
151	N	8.49	116.10	55.15	52.77	40.83	36.62	171.35	172.38	0.682	0.211	0.003
152	L	7.14	123.25	52.77	53.89	36.62	41.19	172.38	177.78	0.892	0.319	0.003
153	Q	7.77	113.21	53.87	55.03	41.19	27.78	177.80	175.86	0.911	0.901	0.003
154	E	6.28	113.90	55.02	50.82	27.79	32.44	175.98	---	0.401	0.013	0.003
155	P											
156	Y	7.78	117.94	63.06	61.95	31.42	39.53	176.70	175.62	0.421	0.018	0.003
157	F	7.88	112.22	61.95	60.07	39.52	40.95	175.66	---	0.843	0.014	0.003
158	T	7.42	106.06	60.06	62.37	40.95	68.69	175.89	175.68	0.702	0.294	0.003

159	W	8.19	124.33	62.37	60.19	68.70	27.96	175.79	171.35	1.000	0.025	0.003
160	P											
161	L	6.24	111.66	65.29	55.38	29.39	41.52	177.89	176.54	1.000	0.515	0.003
162	I	6.79	119.58	55.38	65.08	41.52	36.71	176.56	175.39	0.972	0.707	0.003
163	A	7.82	115.31	65.09	51.66	36.71	17.71	175.41	179.39	0.999	0.961	0.003
164	A	6.48	120.90	51.66	55.72	17.72	19.05	179.39	178.06	0.989	0.741	0.003
165	D	9.40	115.09	55.72	52.79	19.05	41.70	178.06	176.38	0.915	0.120	0.003
166	G	7.31	103.81	52.78	45.16	41.71	---	176.40	175.45	0.961	0.692	0.003
167	G	7.23	108.24	45.15	44.81	---	---	175.47	171.94	1.000	0.850	0.003
168	Y	8.09	115.67	44.79	57.02	---	38.48	171.95	173.19	0.996	0.995	0.002
169	A	10.34	124.46	57.02	52.62	38.47	15.89	173.19	172.93	1.000	0.291	0.003
170	F	6.14	108.10	52.62	55.04	15.89	42.06	172.92	176.47	1.000	0.044	0.003
171	K	8.69	125.78	55.04	56.47	42.05	31.78	176.48	174.48	0.462	0.915	0.003
172	Y	8.42	129.99	56.47	55.73	31.78	39.21	174.57	---	0.720	0.697	0.003
173	E	8.06	126.91	55.72	55.17	39.21	31.86	174.68	175.18	0.150	0.144	0.003
174	N											
175	G	7.29	102.22	53.53	45.11	36.67	---	175.23	172.99	0.164	0.056	0.003
176	K	6.91	118.69	45.10	53.97	---	34.71	173.00	174.49	0.330	0.771	0.002
177	Y	8.55	120.08	53.97	58.30	34.71	39.21	174.51	176.05	0.377	0.342	0.003
178	D	8.84	124.02	58.30	52.27	39.21	40.67	176.07	177.25	0.556	0.459	0.003
179	I	7.54	117.17	52.27	63.03	40.67	36.14	177.27	175.60	0.560	0.248	0.002
180	K	8.12	116.25	63.02	54.12	36.15	31.35	175.61	176.26	0.732	0.405	0.003
181	D	7.59	123.40	54.13	52.75	31.34	40.33	176.28	172.81	0.669	0.443	0.003
182	V	7.30	123.04	52.74	58.69	40.34	34.04	172.83	176.68	0.782	0.770	0.003
183	G	6.89	120.90	58.68	45.84	34.04	---	176.70	173.97	0.924	0.195	0.003
184	V	6.53	114.17	45.82	62.73	---	31.84	173.98	173.06	0.978	0.298	0.002
185	D	7.66	114.97	62.73	51.01	31.84	39.20	173.08	176.00	0.860	0.121	0.003
186	N	6.72	116.42	51.02	---	39.20	38.75	176.01	174.62	0.263	0.104	0.003
187	A	8.32	120.69	51.14	54.90	---	---	174.65	---	0.201	0.805	0.003
188	G	8.20	109.60	54.90	46.73	17.59	---	180.29	175.77	0.566	0.855	0.003
189	A	7.89	127.84	46.72	53.32	---	17.66	175.79	180.62	1.000	0.715	0.002
190	K	7.81	115.35	53.32	59.08	17.65	31.20	180.62	180.12	0.693	0.852	0.003
191	A	8.04	124.83	59.06	55.13	31.20	17.21	180.14	181.11	0.761	0.859	0.003
192	G	8.05	107.75	55.12	47.30	17.21	---	181.12	174.80	0.481	0.812	0.003
193	L	8.49	121.42	47.28	56.64	---	39.45	174.81	178.18	0.350	0.744	0.002
194	T	8.36	116.60	56.65	67.49	39.46	68.00	178.19	---	0.596	0.803	0.003
195	F	7.49	122.25	67.51	61.74	68.01	38.54	175.01	177.23	1.000	0.959	0.003
196	L	7.55	119.75	61.74	58.08	38.54	40.56	177.24	177.91	0.577	0.978	0.003
197	V	8.42	117.66	58.07	66.62	40.56	30.04	177.92	178.28	0.959	0.664	0.003
198	D	8.41	123.09	66.63	57.42	30.07	39.26	178.28	179.14	0.698	0.706	0.003

199	L	7.71	120.39	57.42	57.92	39.26	41.38	179.15	178.91	0.525	0.833	0.003
200	I	7.27	120.16	57.92	62.72	41.39	37.60	178.90	180.89	0.729	0.101	0.003
201	K	9.22	124.66	62.71	59.32	37.59	31.48	180.90	178.59	0.020	0.001	0.003
202	N	7.83	114.07	59.32	53.00	31.49	38.04	178.60	173.01	0.649	0.470	0.003
203	K	7.76	111.63	52.98	56.93	38.04	26.67	173.03	175.57	0.576	0.109	0.003
204	H	8.19	116.38	56.92	56.69	26.67	27.89	175.50	---	0.396	0.704	0.003
205	M	7.30	114.23	56.64	54.03	27.88	36.74	175.39	172.99	0.523	0.903	0.003
206	N	8.54	118.69	54.03	51.54	36.74	39.60	172.99	175.95	0.636	0.886	0.002
207	A	8.89	125.15	51.53	54.06	39.60	17.62	176.04	---	0.569	0.880	0.003
208	D	8.00	112.98	54.06	53.12	17.61	39.27	176.21	---	0.487	0.885	0.003
209	T	7.16	116.69	53.12	66.13	39.26	68.47	176.31	---	0.768	0.424	0.003
210	D	6.81	130.92	66.15	51.25	68.47	41.47	173.06	175.61	1.000	0.020	0.003
211	Y	7.68	117.55	51.24	62.79	41.47	38.44	175.63	178.16	0.971	0.850	0.003
212	S	8.03	114.86	62.79	61.29	38.43	62.24	178.20	177.34	0.751	0.910	0.003
213	I	8.80	124.63	61.28	64.37	62.24	37.90	177.35	178.75	1.000	0.671	0.003
214	A	7.44	120.84	64.37	54.81	37.90	16.42	178.76	177.65	0.641	0.405	0.003
215	E	7.70	117.55	54.81	58.41	16.42	29.20	177.69	---	0.288	0.609	0.003
216	A	7.81	119.89	58.41	54.18	---	17.20	177.94	179.49	0.114	0.965	0.002
217	A	7.64	117.83	54.14	54.35	17.19	19.09	179.53	180.49	0.478	0.806	0.003
218	F	8.16	119.58	54.39	62.34	19.11	37.47	180.49	178.83	0.880	0.481	0.003
219	N											
220	K	7.54	116.90	54.91	55.52	35.69	32.21	176.62	177.54	0.479	0.889	0.003
221	G	7.54	108.84	55.51	45.54	32.21	---	177.54	174.96	0.473	0.945	0.003
222	E											
223	T	6.68	110.66	56.82	57.45	29.80	69.91	175.60	172.52	0.172	0.045	0.003
224	A	8.50	127.52	57.45	54.04	69.91	19.72	172.53	176.24	1.000	0.276	0.003
225	M	7.90	114.41	54.02	---	19.73	39.50	176.25	173.76	0.725	0.482	0.003
226	T	8.98	113.49	53.98	58.98	39.50	70.36	173.77	171.19	0.210	0.074	0.003
227	I	6.98	122.74	58.98	59.74	70.36	39.73	171.19	174.54	1.000	0.256	0.003
228	N	8.25	121.93	59.73	51.82	39.73	42.18	174.55	175.32	0.961	0.985	0.003
229	G	8.08	110.89	51.82	41.78	42.19	---	175.32	169.97	0.906	0.152	0.003
230	P											
231	W	5.81	112.13	62.14	58.54	29.13	25.89	176.15	---	0.967	0.003	0.003
232	A	6.80	125.43	58.54	51.51	25.88	18.37	176.21	179.22	0.998	0.159	0.003
233	W	7.00	118.64	51.51	58.03	18.37	27.91	179.24	177.61	0.723	0.969	0.003
234	S											
235	N											
236	I											
237	D											
238	T											

239	S	7.49	118.45	64.78	60.27	---	63.49	175.66	---	0.659	0.495	0.002
240	K											
241	V											
242	N	8.54	118.64	---	51.54	---	36.74	---	---	0.035	0.365	0.001
243	Y	7.70	121.92	51.50	54.19	36.76	40.89	173.19	174.20	0.906	0.856	0.003
244	G	8.33	107.63	54.19	42.39	40.90	---	174.22	170.95	0.752	0.655	0.003
245	V	8.05	120.88	42.39	60.85	---	33.54	170.95	174.65	0.936	0.938	0.001
246	T	9.31	119.71	60.85	58.41	33.55	70.92	174.66	174.00	0.446	0.443	0.003
247	V	7.96	123.66	58.41	61.74	70.93	31.41	174.01	174.91	0.974	0.551	0.003
248	L	8.41	125.67	61.74	53.87	31.41	39.66	174.93	173.94	0.605	0.500	0.003
249	P											
250	T	8.62	111.78	61.36	60.41	29.67	70.76	175.34	174.39	0.421	0.610	0.003
251	F	9.39	122.03	60.40	56.23	70.76	41.13	174.41	---	0.967	0.978	0.003
252	K	10.39	129.84	56.22	56.75	41.13	27.59	174.46	177.47	0.989	0.185	0.003
253	G	8.85	103.71	56.75	44.48	27.59	---	177.47	173.66	0.929	0.729	0.003
254	Q	8.07	122.50	44.46	51.74	---	28.70	173.67	172.53	1.000	0.606	0.002
255	P											
256	S	7.81	118.09	62.67	61.62	30.58	64.30	178.39	173.33	0.251	0.238	0.003
257	K	7.49	123.48	61.62	52.46	64.30	32.64	173.35	171.29	1.000	0.007	0.003
258	P											
259	F	9.17	118.57	61.67	57.87	30.83	39.46	176.95	177.43	0.677	0.686	0.003
260	V	8.58	122.03	57.87	61.29	39.46	33.07	177.44	176.28	0.609	0.843	0.003
261	G	8.68	116.11	61.29	44.26	33.09	---	176.30	171.39	0.969	0.883	0.003
262	V	10.87	129.28	44.25	59.58	---	32.03	171.41	179.17	0.070	0.000	0.002
263	L	8.64	134.43	59.57	56.01	32.05	40.34	179.17	175.01	0.018	0.000	0.003
264	S	8.60	126.75	56.00	58.19	40.33	65.59	175.02	170.49	0.126	0.012	0.003
265	A	8.97	123.85	58.18	49.24	65.59	20.20	170.51	175.95	1.000	0.822	0.003
266	G	9.25	110.91	49.23	42.33	20.20	---	175.97	170.89	1.000	0.651	0.003
267	I	10.12	123.85	42.32	59.23	---	38.88	170.90	175.04	1.000	0.382	0.002
268	N	8.01	125.84	59.23	52.88	38.89	38.18	175.06	177.06	0.759	0.870	0.003
269	A	8.67	131.16	52.88	54.69	38.18	17.25	177.07	178.20	0.718	0.926	0.003
270	A	8.00	118.36	54.69	50.75	17.27	17.91	178.21	177.89	0.775	0.987	0.003
271	S	7.37	113.84	50.74	55.59	17.91	63.86	177.90	175.54	0.703	0.715	0.003
272	P											
273	N	8.61	119.74	63.02	52.06	30.55	39.86	175.80	176.04	0.024	0.054	0.003
274	K	7.66	119.43	52.05	61.10	39.87	31.12	176.08	178.67	0.542	0.397	0.003
275	E	8.64	118.47	61.10	59.34	31.12	27.38	178.68	179.51	0.554	0.849	0.003
276	L	7.28	120.90	59.34	56.76	27.39	41.47	179.53	178.23	0.416	0.825	0.003
277	A	8.15	120.50	56.76	54.80	41.47	17.18	178.30	---	0.327	0.961	0.003
278	K	7.65	118.42	54.80	59.62	17.19	31.75	178.37	177.03	0.711	0.823	0.003

279	E	7.43	118.95	59.61	59.07	31.75	28.57	177.04	179.06	0.348	0.794	0.003
280	F	8.23	118.59	59.06	61.04	28.57	37.80	179.06	177.36	0.379	0.998	0.003
281	L	8.18	120.15	61.05	57.68	37.80	40.05	177.37	176.92	0.469	0.959	0.003
282	E	8.24	113.06	57.68	58.93	40.05	28.82	176.90	---	0.145	0.208	0.003
283	N	7.56	109.08	58.92	52.48	28.82	38.45	176.87	175.00	0.902	0.845	0.003
284	Y	7.00	116.44	52.47	59.19	38.44	37.36	175.02	176.05	0.415	0.784	0.003
285	L	7.91	121.50	59.19	57.54	37.36	40.36	176.07	176.75	0.329	0.400	0.003
286	L	7.65	119.47	57.54	53.82	40.31	37.73	176.75	174.74	0.090	0.058	0.003
287	T	8.37	108.93	53.82	58.32	37.74	72.95	174.76	174.07	0.304	0.221	0.003
288	D	8.35	122.26	58.32	57.81	72.95	38.96	174.08	177.56	1.000	0.979	0.003
289	E	8.24	115.81	57.80	58.70	38.95	28.57	177.57	179.90	0.348	0.974	0.003
290	G	8.15	113.03	58.69	46.33	28.57	---	179.91	175.05	0.889	0.130	0.003
291	L											
292	E	7.56	121.15	56.68	58.84	40.95	28.38	179.67	177.96	0.395	0.910	0.003
293	A	7.01	120.05	58.83	54.67	28.42	16.97	177.98	180.60	0.336	0.873	0.003
294	V	7.08	116.02	54.66	66.70	16.97	31.04	180.60	176.83	0.471	0.684	0.003
295	N	8.53	117.56	66.71	55.64	31.05	40.35	176.85	176.15	0.260	0.239	0.003
296	K	8.09	116.13	55.63	57.79	40.36	31.46	176.17	177.20	0.284	0.552	0.003
297	D	7.10	118.96	57.78	55.56	31.47	41.47	177.22	175.83	0.551	0.977	0.003
298	K	7.46	116.03	55.55	52.00	41.47	34.10	175.85	173.03	0.774	0.485	0.003
299	P											
300	L	8.44	122.71	62.61	55.10	30.99	43.12	178.63	177.27	0.907	0.977	0.003
301	G	7.97	104.31	55.09	43.78	43.13	---	177.27	173.62	0.963	0.851	0.003
302	A	7.35	124.02	43.77	50.85	---	18.79	173.63	177.44	1.000	0.259	0.002
303	V	7.99	110.57	50.85	59.03	18.79	34.56	177.46	173.46	0.990	0.193	0.003
304	A	7.47	117.27	59.02	52.48	34.56	18.82	173.47	175.55	0.990	0.041	0.003
305	L	6.21	115.16	52.47	53.71	18.82	43.32	175.56	175.30	0.753	0.052	0.003
306	K	7.88	129.60	53.71	59.94	43.32	31.07	175.27	178.43	0.930	0.532	0.003
307	S	8.56	112.51	59.93	60.81	31.07	59.94	178.43	---	0.512	0.700	0.003
308	Y	6.46	122.43	60.81	58.52	59.94	37.92	176.91	177.18	1.000	0.213	0.003
309	E	8.33	121.56	58.52	57.39	37.92	25.15	177.21	177.53	0.167	0.085	0.003
310	E	7.56	115.71	57.39	58.65	25.21	28.33	177.55	178.58	0.061	0.044	0.003
311	E	6.71	117.68	58.64	57.28	28.39	---	178.59	179.02	0.063	0.229	0.003
312	L	8.06	121.18	57.27	56.56	28.55	41.14	179.01	179.46	0.266	0.711	0.003
313	A	8.40	118.16	56.56	53.41	41.14	16.41	179.45	176.46	0.801	0.891	0.002
314	K	6.93	117.56	53.40	58.28	16.40	31.21	176.46	177.92	0.343	0.027	0.003
315	D	8.13	121.39	58.27	50.01	31.21	40.77	177.93	175.43	0.451	0.079	0.003
316	P											
317	R	8.27	117.04	63.87	57.86	31.48	28.75	178.97	179.60	0.411	0.920	0.003
318	I	7.36	123.15	57.85	63.98	28.75	35.72	179.61	177.75	0.552	0.349	0.003

319	A	7.69	122.74	63.97	55.23	35.73	16.69	177.77	181.10	0.619	0.775	0.003
320	A	7.97	118.91	55.23	54.60	16.70	17.31	181.11	178.64	0.417	0.974	0.003
321	T	7.17	114.92	54.60	67.49	17.30	68.28	178.65	---	1.000	0.964	0.003
322	M	7.99	119.51	67.50	56.49	68.23	30.80	175.18	177.13	0.813	0.918	0.003
323	E	8.06	121.61	56.48	59.31	30.81	28.18	177.14	179.23	0.443	0.913	0.003
324	N	8.12	115.42	59.30	56.32	28.20	38.56	179.24	176.82	0.205	0.737	0.003
325	A	7.92	119.86	56.31	53.82	38.56	17.02	176.83	180.01	0.254	0.541	0.003
326	Q	8.08	116.43	53.81	57.76	17.02	27.36	180.02	177.53	0.133	0.464	0.003
327	K	6.88	117.95	57.76	55.54	27.35	32.05	177.54	175.91	0.413	0.631	0.003
328	G	7.08	106.56	55.53	44.04	32.06	---	175.92	172.35	0.639	0.982	0.003
329	E	8.35	121.96	44.04	53.34	---	32.17	172.36	176.51	0.754	0.592	0.002
330	I	9.00	128.17	53.34	61.73	32.20	37.07	176.53	177.99	0.540	0.328	0.003
331	M	8.32	125.22	61.73	55.84	37.07	33.75	178.00	174.00	0.260	0.153	0.003
332	P											
333	N	7.43	118.37	62.99	50.95	30.57	37.79	175.23	---	0.504	0.222	0.003
334	I	6.21	108.72	50.94	59.01	37.78	35.95	175.41	176.03	0.991	0.217	0.003
335	P											
336	Q	8.18	116.68	64.84	58.83	30.68	25.82	178.55	176.83	0.590	0.784	0.003
337	M	7.67	118.60	58.82	55.44	25.82	29.65	176.85	177.32	0.180	0.272	0.003
338	S	7.78	113.84	55.44	61.68	29.65	---	177.34	---	0.048	0.081	0.003
339	A	7.18	123.12	61.69	54.04	---	17.96	177.03	179.77	0.173	0.138	0.002
340	F	7.35	119.06	54.03	61.19	17.96	38.33	179.78	176.34	0.436	0.889	0.003
341	W	8.60	118.23	61.19	59.41	38.33	29.71	176.36	178.80	0.233	0.446	0.003
342	Y	7.26	114.83	59.41	60.83	29.71	37.79	178.81	178.38	0.250	0.115	0.003
343	A	8.26	122.04	60.83	54.30	37.79	18.47	178.41	180.65	0.802	0.932	0.003
344	V	8.50	118.62	54.29	66.35	18.46	29.97	180.65	176.87	0.550	0.758	0.003
345	R	7.94	120.96	66.36	59.68	29.98	29.29	176.89	178.25	0.868	0.641	0.003
346	T	7.36	113.82	59.68	65.90	29.30	68.65	178.27	---	0.477	0.943	0.003
347	A	7.61	123.00	65.91	54.88	68.65	18.05	174.88	178.98	1.000	0.955	0.003
348	V	7.98	116.11	54.86	66.87	18.04	30.35	178.98	177.81	0.504	0.993	0.003
349	I	7.59	118.53	66.88	64.32	30.36	36.74	177.82	180.09	1.000	0.907	0.003
350	N	8.64	121.25	64.32	55.28	36.75	36.55	180.09	177.82	0.883	0.256	0.003
351	A	8.34	122.89	55.28	53.40	36.53	17.26	177.83	180.91	0.311	0.130	0.003
352	A	8.98	121.18	53.39	54.93	17.29	---	180.92	178.07	0.003	0.000	0.003
353	S	7.27	111.59	---	---	---	---	177.96	---	0.052	0.901	0.003
354	G	7.44	109.98	58.30	45.15	63.77	---	175.51	174.30	0.270	0.298	0.003
355	R	8.05	121.18	45.14	57.86	---	30.09	174.32	176.60	0.403	0.662	0.002
356	Q	7.16	113.81	57.86	53.64	30.10	35.67	176.61	175.84	0.003	0.002	0.003
357	T	8.39	111.93	53.63	60.81	35.66	69.93	175.85	174.71	0.000	0.000	0.003
358	V	8.55	121.50	60.81	67.26	69.93	30.52	174.73	176.94	1.000	0.169	0.003

359	D	7.94	115.28	67.27	57.05	30.53	40.29	176.95	178.66	0.543	0.880	0.003
360	E	7.42	118.80	57.05	58.21	40.29	29.47	178.68	178.38	0.193	0.953	0.003
361	A	8.52	121.68	58.21	54.23	29.48	17.65	178.39	181.30	0.239	0.462	0.003
362	L	7.90	116.39	54.22	56.86	17.65	39.10	181.31	178.68	0.643	0.649	0.003
363	K	7.52	121.66	56.85	59.18	39.10	30.86	178.70	179.67	0.288	0.697	0.003
364	D	7.97	120.22	59.17	56.62	30.86	39.82	179.69	178.66	0.196	0.277	0.003
365	A	7.56	121.45	56.62	54.68	39.82	17.09	178.69	178.19	0.109	0.372	0.003
366	Q	8.07	118.32	54.67	59.76	17.09	26.62	178.21	179.06	0.419	0.615	0.003
367	T	7.98	115.40	59.75	65.56	26.70	68.39	179.07	175.71	0.448	0.728	0.003
368	R	7.79	120.23	65.56	58.25	68.38	29.18	175.74	178.49	0.481	0.990	0.003
369	I	7.82	117.54	58.25	64.32	29.18	38.01	178.51	176.94	0.527	0.998	0.003
370	T	7.46	107.21	64.32	61.91	38.01	69.78	176.96	174.06	0.845	0.927	0.003
371	K	7.23	128.39	61.91	58.32	69.78	31.87	174.08	181.59	0.982	0.000	0.003

Assigned amide groups that match the reference assignment are indicated by green shading. Missing amide groups that are present in the reference assignments are indicated by white shading. Amide groups that were assigned to residues that are not assigned in the reference assignments are indicated by orange shading. Amide groups that do not match those in the reference assignments are indicated with red shading. Amide groups indicated with blue shading are matching assignments that were confirmed manually, but were not assigned in the original reference assignment set. Residues that were not assigned by either BARASA or in the reference assignments were considered matching. Non-amide resonances were not considered when determining assignment accuracy.
The table is included in Excel format in the Source Data file.

Supplementary Table 7 – Assignments of CY1 by BARASA												
**Index**	**AA**	**H**	**N**	**CA(i-1)**	**CA**	**CB(i-1)**	**CB**	**CO(i-1)**	**CO**	**Posterior**	**Likelihood**	**Prior**
1	M											
2	P											
3	D											
4	E	8.65	121.99	54.34	56.30	40.76	29.39	176.08	176.52	0.261	1.000	0.0026
5	S	8.37	116.70	56.32	58.03	29.41	63.46	176.52	174.05	0.237	1.000	0.0026
6	S	7.95	117.47	58.04	57.40	63.46	---	174.05	173.36	0.284	0.998	0.0025
7	W											
8	P											
9	N											
10	M	11.73	123.22	51.45	57.04	39.89	33.77	177.41	---	0.390	0.257	0.0007
11	T	7.61	110.23	57.08	62.35	33.80	67.51	177.40	173.48	0.309	0.975	0.0013
12	E											
13	S											
14	T											
15	P											
16	F											
17	P											
18	L	7.29	116.47	63.26	53.33	32.16	42.30	175.49	180.32	0.021	0.033	0.0026
19	T	9.78	114.58	53.31	60.56	42.33	68.42	180.33	173.98	0.797	0.211	0.0026
20	P											
21	V	6.70	118.13	---	---	30.18	31.20	---	---	0.094	0.278	0.0026
22	Q	6.81	120.50	64.76	---	31.10	---	---	178.57	0.017	0.118	0.0006
23	H	8.72	116.91	58.91	59.81	31.65	30.04	178.59	178.23	0.029	0.026	0.0026
24	A	7.96	123.14	59.82	54.78	30.13	16.76	178.22	181.56	0.134	0.217	0.0013
25	Y	8.17	118.97	54.69	56.88	16.81	36.48	181.57	178.19	0.881	0.300	0.0007
26	L	8.83	120.97	56.87	57.81	36.54	41.60	178.24	---	0.194	0.765	0.0013
27	T	7.93	109.27	57.72	64.33	41.54	---	178.13	---	0.155	0.050	0.0013
28	G	8.16	109.14	64.39	46.82	69.14	---	177.88	174.56	0.936	0.584	0.0013
29	R	7.56	115.60	46.81	55.80	---	30.12	174.59	176.36	0.202	0.475	0.0013
30	M	6.69	118.12	55.80	53.63	---	31.20	176.39	176.75	0.107	0.551	0.0013
31	P											
32	G											
33	Q											
34	T											
35	L	9.99	124.63	64.18	55.74	68.24	37.68	175.01	176.08	0.306	0.118	0.0026
36	G	7.69	104.47	---	43.50	---	---	176.10	177.36	0.223	0.020	0.0012
37	G											
38	V											

39	G									
40	C									
41	H									
42	L									
43	Y	8.71	127.19	---	54.64	---	---	174.72	---	0.006	0.498	0.0006
44	Q	8.71	127.26	54.67	53.32	41.03	31.86	174.66	172.71	0.324	0.783	0.0026
45	E	8.47	123.38	53.35	---	31.85	---	172.71	177.29	0.020	0.149	0.0013
46	F	9.39	122.67	53.45	55.66	29.57	41.73	177.39	174.70	0.360	0.233	0.0026
47	E	9.14	120.27	55.61	54.08	41.72	31.25	174.68	176.88	0.249	0.746	0.0026
48	G	7.39	112.01	54.09	45.61	31.25	---	176.89	171.68	0.725	0.300	0.0013
49	H	8.88	120.84	45.62	55.66	---	33.40	171.69	176.03	0.003	0.003	0.0013
50	C	8.99	109.38	55.67	61.43	33.39	25.48	176.01	173.25	0.018	0.000	0.0026
51	L	8.03	124.23	61.42	55.68	25.47	---	173.25	175.61	0.059	0.228	0.0013
52	T	7.38	112.03	55.69	58.34	39.55	71.53	175.61	174.99	0.047	0.035	0.0013
53	A	9.01	123.64	58.33	55.38	71.54	17.63	174.99	179.38	0.813	0.981	0.0026
54	S	8.57	111.51	55.39	60.98	17.66	61.94	179.38	---	0.539	0.997	0.0026
55	Q	7.39	121.07	60.96	58.02	61.90	28.63	177.73	179.18	0.289	0.981	0.0026
56	L	7.81	120.02	58.02	57.40	28.63	41.59	179.20	177.59	0.133	0.956	0.0026
57	E	9.10	118.46	57.40	59.74	41.64	29.27	177.58	179.48	0.213	0.968	0.0013
58	Q											
59	A	7.51	121.40	58.55	54.61	27.85	18.18	178.25	179.04	0.116	0.992	0.0007
60	I	8.48	117.91	54.59	64.85	18.14	---	---	177.57	0.311	0.972	0.0013
61	T	8.33	115.27	64.76	67.40	36.32	---	177.59	---	0.972	0.932	0.0025
62	T	7.53	118.98	67.37	66.64	---	67.82	177.15	---	0.463	0.808	0.0013
63	L	8.25	123.32	66.63	58.74	67.75	40.49	176.64	178.93	0.377	0.882	0.0026
64	L	8.61	117.46	58.74	57.60	40.34	41.51	179.04	---	0.288	0.734	0.0026
65	Q	7.37	116.73	57.56	56.63	41.45	28.27	179.07	177.59	0.143	0.917	0.0013
66	R	7.54	119.33	56.63	58.13	---	32.71	177.62	175.51	0.014	0.230	0.0006
67	H	7.42	115.65	58.11	53.13	32.70	---	175.51	174.78	0.082	0.263	0.0025
68	P											
69	M	8.78	115.04	65.25	56.30	31.39	29.41	---	179.16	0.047	0.170	0.0003
70	L	8.12	116.11	56.29	---	29.46	39.68	179.16	175.88	0.086	0.131	0.0026
71	H	8.48	114.19	56.19	54.82	39.69	29.34	175.88	175.20	0.288	0.746	0.0026
72	I	7.24	125.35	54.77	59.75	29.45	38.47	175.21	174.18	0.041	0.072	0.0013
73	A	8.59	123.22	59.74	49.54	38.35	21.65	174.14	175.48	0.913	0.249	0.0026
74	F	7.18	116.96	49.54	58.28	21.65	40.50	175.49	---	0.109	0.005	0.0013
75	R	8.38	121.77	58.27	53.35	40.52	---	175.67	176.63	0.018	0.155	0.0013
76	P											
77	D	7.88	115.91	63.86	52.77	31.01	38.75	---	176.73	0.145	0.971	0.0007
78	G	8.39	107.38	52.79	46.03	38.72	---	176.76	173.23	0.962	0.873	0.0025

79	Q	7.01	115.16	46.03	54.20	---	33.56	173.22	172.80	0.107	0.110	0.0013
80	Q	8.79	119.06	54.25	52.07	33.50	29.90	172.78	173.08	0.117	0.168	0.0013
81	V	8.43	117.65	52.05	59.65	29.92	36.03	173.10	170.99	0.086	0.129	0.0013
82	W	9.18	129.72	59.65	58.91	35.99	29.96	171.00	175.03	0.057	0.016	0.0026
83	L	7.72	128.15	58.92	50.86	29.99	41.17	174.92	---	0.284	0.347	0.0026
84	P											
85	Q											
86	P											
87	Y											
88	W											
89	N											
90	G											
91	V	9.32	121.07	43.71	---	---	33.49	173.04	176.51	0.569	0.392	0.0013
92	T	7.69	126.84	62.20	63.57	33.50	68.68	176.52	172.62	0.377	0.403	0.0013
93	V	8.64	129.34	63.57	60.57	68.70	33.25	172.61	175.18	0.409	0.868	0.0013
94	H	9.48	129.80	60.57	53.74	33.29	30.46	175.22	174.28	0.621	0.653	0.0026
95	D	9.14	123.81	53.71	53.39	30.47	39.34	174.26	175.59	0.287	0.740	0.0026
96	L	8.52	127.10	53.27	52.60	39.38	40.83	175.60	178.93	0.373	0.873	0.0013
97	R	7.73	118.15	52.60	60.07	---	29.49	178.95	177.28	0.070	0.368	0.0013
98	H											
99	N	7.24	118.77	54.86	53.53	29.58	39.89	175.03	175.36	0.201	0.780	0.0013
100	D	8.61	120.46	53.55	53.15	39.93	40.45	175.39	---	0.323	0.737	0.0026
101	A	8.67	121.70	53.09	55.75	---	17.87	175.44	180.43	0.557	0.798	0.0013
102	E	8.60	118.69	55.73	59.68	17.90	28.46	180.44	179.73	0.368	0.965	0.0026
103	S	8.59	117.43	59.69	61.32	28.49	61.98	179.73	---	0.239	0.938	0.0026
104	R	8.50	123.52	61.32	59.70	61.98	29.27	178.49	177.19	0.231	0.579	0.0026
105	Q	7.99	118.03	59.68	58.14	29.23	27.52	177.18	178.03	0.080	0.986	0.0013
106	A	7.93	120.27	58.14	54.64	27.54	17.33	178.04	180.77	0.221	0.999	0.0026
107	Y	8.19	120.99	54.65	60.72	17.32	38.07	180.78	179.22	0.528	0.923	0.0013
108	L	8.57	119.90	60.72	57.57	38.17	40.77	179.33	180.31	0.143	0.951	0.0007
109	D	8.50	122.11	57.58	57.06	40.78	39.88	180.33	178.45	0.222	0.825	0.0026
110	A	7.72	123.44	57.03	54.48	39.88	16.88	178.45	180.46	0.175	0.998	0.0013
111	L	8.33	120.82	54.46	57.16	16.85	41.84	180.46	177.88	0.561	0.941	0.0013
112	R	7.76	118.23	57.15	59.18	41.86	30.54	177.90	178.24	0.079	0.420	0.0013
113	Q	7.71	116.08	59.20	58.23	30.44	---	178.23	---	0.044	0.650	0.0013
114	R	7.60	117.27	58.23	57.92	28.43	27.50	178.22	---	0.087	0.530	0.0026
115	L	8.13	117.01	57.91	55.87	---	42.41	---	---	0.091	0.825	0.0013
116	S	8.08	113.86	55.90	61.41	42.56	62.35	180.25	---	0.750	0.416	0.0013
117	H	8.05	119.86	---	54.28	62.33	---	173.94	173.00	0.082	0.418	0.0013
118	R	6.23	119.38	54.24	55.97	27.13	31.56	172.99	---	0.243	0.057	0.0013

119	L	8.24	127.11	55.97	51.88	31.61	40.83	173.25	176.92	0.082	0.142	0.0026
120	L	9.18	130.14	51.83	55.76	41.03	39.06	176.96	178.21	0.008	0.006	0.0026
121	R	9.88	124.79	55.76	54.58	39.09	27.39	178.17	---	0.004	0.008	0.0013
122	V	7.87	120.29	54.60	64.27	28.92	---	177.99	178.53	0.145	0.612	0.0013
123	E	10.17	124.11	64.29	58.00	29.10	27.64	178.53	177.43	0.250	0.027	0.0013
124	I	7.52	111.30	58.08	59.69	---	37.36	177.43	175.30	0.178	0.777	0.0013
125	G	7.55	106.48	59.73	44.35	37.46	---	175.27	173.41	0.855	0.654	0.0025
126	E	7.22	123.09	44.35	54.47	---	28.13	173.43	---	0.297	0.186	0.0013
127	T	7.96	114.29	54.47	60.70	28.17	69.17	173.45	172.73	0.028	0.110	0.0013
128	F	7.48	114.24	60.80	55.33	69.20	39.78	172.74	---	0.455	0.425	0.0013
129	D	9.30	115.78	55.32	54.28	39.82	42.82	172.94	173.98	0.407	0.340	0.0013
130	F	8.96	120.56	54.22	57.03	42.81	---	173.79	---	0.025	0.278	0.0006
131	Q	9.29	117.60	57.01	53.89	43.51	---	---	173.73	0.088	0.304	0.0013
132	L	9.33	126.13	53.83	53.45	---	47.04	173.74	173.26	0.072	0.056	0.0013
133	T	10.12	127.74	53.37	61.65	47.03	67.40	173.27	174.94	0.900	0.004	0.0013
134	L	8.89	126.27	61.65	---	67.39	41.41	174.98	---	0.367	0.376	0.0013
135	L	8.08	122.75	53.59	51.64	41.40	41.82	174.73	173.69	0.533	0.668	0.0013
136	P											
137	D	8.89	117.79	62.94	55.25	31.54	38.87	175.77	175.53	0.072	0.959	0.0007
138	N	8.87	110.19	55.25	54.35	38.86	37.34	175.51	174.20	0.557	0.961	0.0026
139	R	7.14	119.11	54.34	55.72	37.34	31.50	174.21	174.85	0.195	0.877	0.0013
140	H	8.26	118.67	55.83	---	31.56	35.33	174.90	173.81	0.007	0.030	0.0013
141	R	9.28	122.00	56.01	54.48	35.32	33.26	173.83	173.40	0.006	0.020	0.0013
142	L	9.86	129.15	54.47	52.89	33.26	44.64	173.40	174.56	0.387	0.635	0.0026
143	H	9.87	127.38	52.88	54.77	44.59	29.38	174.53	---	0.447	0.509	0.0026
144	V	8.76	125.72	54.78	61.25	29.45	34.44	174.70	173.42	0.623	0.402	0.0026
145	N	8.58	123.56	61.31	---	34.42	44.24	173.42	174.90	0.001	0.006	0.0007
146	I	9.02	119.55	53.77	61.59	44.27	42.72	174.90	173.32	0.006	0.000	0.0013
147	D	8.76	128.84	61.58	53.92	42.70	44.16	173.29	175.45	0.095	0.011	0.0013
148	L											
149	L	7.70	121.58	55.34	---	---	---	178.63	177.82	0.031	0.523	0.0024
150	I	7.53	104.13	58.16	61.39	---	39.32	177.83	173.90	0.352	0.386	0.0013
151	M	6.42	111.41	61.37	54.23	---	---	173.87	---	0.210	0.157	0.0024
152	D	8.95	120.55	54.23	---	---	43.11	173.91	---	0.078	0.599	0.0013
153	A											
154	S											
155	S											
156	F											
157	T											
158	L	6.51	120.02	65.21	56.99	68.36	40.68	174.58	179.25	0.517	0.075	0.0013

159	F	7.69	119.73	57.04	59.65	40.76	38.71	---	176.72	0.026	0.133	0.0007
160	F	8.94	114.89	---	60.27	38.66	---	---	178.62	0.020	0.237	0.0006
161	D	8.79	121.01	60.30	57.93	---	38.90	178.62	179.34	0.042	0.428	0.0013
162	E	8.11	121.06	---	---	38.95	28.52	179.29	178.24	0.058	0.749	0.0013
163	L	8.35	115.58	58.11	---	---	40.49	---	177.91	0.003	0.139	0.0006
164	N	8.35	115.59	58.11	55.09	40.57	38.53	177.91	177.42	0.199	0.776	0.0013
165	A	8.31	122.29	55.10	---	38.56	---	177.40	179.93	0.098	0.593	0.0025
166	L	8.15	117.55	55.31	57.21	17.81	41.16	179.96	182.67	0.982	0.844	0.0026
167	L	8.45	121.20	---	57.01	41.14	40.77	182.64	178.63	0.321	0.628	0.0013
168	A	8.03	120.20	56.97	51.73	40.72	18.43	178.64	177.55	0.616	0.997	0.0026
169	G	7.87	107.36	51.72	45.28	18.43	---	177.56	174.95	1.000	0.997	0.0025
170	E	8.24	120.33	45.28	55.12	---	30.00	174.95	176.04	0.263	0.993	0.0013
171	S	8.30	114.79	55.11	57.14	30.01	63.64	176.04	174.04	0.248	0.995	0.0026
172	L	8.47	124.17	57.15	51.73	63.66	41.49	174.05	175.20	0.628	0.854	0.0026
173	P											
174	A	8.57	125.13	61.88	52.40	31.35	18.10	176.61	178.04	0.494	0.958	0.0026
175	I	8.34	122.56	52.40	58.28	18.09	38.75	178.05	175.80	0.840	0.605	0.0026
176	D	8.84	126.88	58.28	54.02	38.77	41.02	175.80	177.64	0.465	0.740	0.0026
177	T	8.43	117.55	54.02	61.85	40.99	68.89	177.64	175.64	0.293	0.907	0.0007
178	R	8.68	121.08	61.84	57.12	68.90	30.00	---	175.75	0.169	0.972	0.0013
179	Y	7.09	121.96	57.12	56.58	29.99	38.89	175.77	173.33	0.116	0.390	0.0013
180	D	8.12	126.28	56.54	51.42	38.91	42.47	173.32	176.96	0.670	0.642	0.0026
181	F	8.35	116.68	51.41	59.86	42.43	38.64	176.94	177.28	0.571	0.888	0.0026
182	R	7.52	116.30	59.83	---	38.66	28.26	177.30	---	0.149	0.700	0.0013
183	S	7.34	115.15	---	---	28.21	60.73	177.46	---	0.333	0.939	0.0026
184	Y	8.07	118.97	60.16	59.72	60.75	38.52	174.75	176.68	0.169	0.215	0.0026
185	L	8.02	117.98	59.67	57.31	38.51	40.84	176.71	181.58	0.122	0.387	0.0007
186	L	7.84	121.40	57.40	---	40.90	---	181.59	178.77	0.023	0.192	0.0013
187	H	8.41	124.92	57.46	56.54	41.04	---	178.81	178.25	0.000	0.001	0.0013
188	Q	8.88	117.24	56.55	58.68	28.82	---	178.25	---	0.002	0.026	0.0013
189	Q	7.80	118.14	---	58.50	28.82	27.74	---	178.28	0.023	0.855	0.0007
190	K	7.51	117.68	---	58.40	27.75	31.97	178.30	179.41	0.126	0.987	0.0007
191	I	8.04	116.45	58.38	62.57	32.01	37.54	179.42	177.92	0.296	0.915	0.0013
192	N	8.51	115.18	62.56	52.64	37.56	---	177.93	176.89	0.332	0.844	0.0025
193	Q	7.51	122.25	52.67	61.30	37.59	25.04	176.92	---	0.183	0.256	0.0007
194	P											
195	L	6.90	116.29	65.50	56.24	30.16	40.60	178.96	180.32	0.405	0.964	0.0013
196	R	8.46	122.31	56.25	60.28	40.67	29.20	180.32	---	0.115	0.519	0.0013
197	D	8.18	120.30	60.30	57.51	29.21	39.27	177.91	179.54	0.114	0.675	0.0026
198	D	8.16	121.39	57.50	56.92	39.24	40.64	179.49	179.12	0.155	0.843	0.0026

199	A	8.51	123.03	56.92	55.11	40.69	18.99	179.09	178.58	0.224	0.784	0.0026
200	R	8.42	121.02	55.12	59.23	18.99	28.30	178.59	177.18	0.385	0.669	0.0013
201	A	7.31	118.64	59.30	54.73	28.39	17.18	177.16	180.55	0.186	0.764	0.0013
202	Y	7.80	119.97	54.69	60.42	17.18	37.44	180.55	178.07	0.282	0.722	0.0013
203	W	8.70	120.41	---	60.24	37.43	29.95	178.06	179.33	0.158	0.761	0.0013
204	L	8.85	117.84	60.20	57.30	29.93	39.51	179.33	180.29	0.142	0.519	0.0013
205	A	7.65	120.72	57.29	54.06	39.55	17.19	180.30	179.50	0.242	0.982	0.0026
206	K	7.30	114.47	54.04	56.55	17.18	31.99	179.52	178.68	0.595	0.696	0.0007
207	A	7.88	122.80	56.54	55.57	31.99	17.92	178.39	179.34	0.392	0.772	0.0013
208	S	8.46	110.72	55.58	---	17.86	---	179.33	---	0.163	0.633	0.0006
209	T	7.89	109.68	---	60.75	62.51	69.43	---	174.72	0.154	0.990	0.0007
210	L	7.15	122.84	60.78	53.53	69.47	40.47	174.72	---	0.581	0.990	0.0013
211	P											
212	P											
213	A	8.27	121.86	62.50	51.31	31.08	16.02	176.97	174.24	0.611	0.706	0.0007
214	P											
215	V	6.95	124.03	64.07	61.75	30.69	32.59	174.70	---	0.022	0.010	0.0026
216	L	8.19	127.09	61.75	50.69	32.58	44.03	175.85	174.84	0.140	0.512	0.0007
217	P											
218	L	7.70	119.74	61.93	52.47	30.25	42.45	174.19	179.02	0.028	0.054	0.0026
219	A	9.42	126.01	52.47	51.82	42.45	18.91	179.03	176.84	0.939	0.160	0.0026
220	C	7.27	111.13	51.78	54.31	18.86	29.48	176.85	171.83	0.999	0.567	0.0007
221	E	8.38	119.79	54.31	53.51	29.51	28.85	171.83	177.34	0.597	0.432	0.0026
222	P											
223	A	9.01	116.62	65.93	54.10	31.75	18.39	177.56	178.29	0.387	0.236	0.0026
224	T	8.07	106.75	54.09	61.83	18.39	69.74	178.29	174.86	0.972	0.865	0.0007
225	L	7.13	123.73	61.80	54.86	69.74	40.77	174.85	177.00	0.468	0.989	0.0013
226	R	8.58	125.22	54.90	57.21	40.73	30.05	176.99	---	0.162	0.982	0.0013
227	E	7.62	116.50	57.20	54.48	29.96	30.95	176.26	174.24	0.251	0.986	0.0026
228	V	8.37	125.21	54.48	62.75	31.02	32.16	174.25	175.05	0.849	0.985	0.0026
229	R	8.20	127.04	62.73	---	---	31.21	174.89	175.85	0.066	0.870	0.0013
230	N	9.68	117.56	---	52.04	---	40.01	176.59	175.88	0.010	0.054	0.0006
231	T	9.68	117.58	52.05	62.42	40.01	71.91	175.93	171.88	0.706	0.246	0.0026
232	R	8.58	127.42	62.42	54.52	71.91	31.22	171.86	175.71	0.231	0.436	0.0026
233	R	8.90	127.83	54.49	---	31.27	30.31	175.73	174.02	0.347	0.742	0.0026
234	R	8.58	121.27	54.53	---	30.27	34.38	174.04	174.51	0.105	0.374	0.0026
235	M	9.10	125.11	53.72	54.94	34.39	35.88	174.55	172.89	0.144	0.510	0.0013
236	I	8.37	127.04	55.03	59.84	35.88	38.20	172.89	175.72	0.529	0.760	0.0026
237	V	9.58	132.01	59.84	58.87	38.23	32.54	175.73	---	0.530	0.397	0.0013
238	P											

239	A	9.15	124.18	62.01	56.13	32.43	18.32	177.07	180.34	0.999	0.938	0.0026
240	T	8.62	109.06	---	65.40	---	---	180.34	---	0.296	0.648	0.0012
241	R	7.03	122.58	65.38	59.15	67.91	30.22	---	177.48	0.145	0.714	0.0007
242	W	8.90	120.81	---	58.63	30.26	29.20	177.48	178.75	0.105	0.544	0.0013
243	H	8.97	119.31	58.69	---	29.20	---	178.77	177.19	0.053	0.584	0.0025
244	A	7.30	121.03	58.85	55.16	29.23	17.68	177.17	179.90	0.163	0.914	0.0013
245	F	8.23	119.59	55.18	61.79	17.72	39.23	179.91	176.67	0.289	0.960	0.0013
246	S	8.57	112.26	61.77	---	39.22	---	176.68	---	0.115	0.785	0.0013
247	N	8.43	122.12	61.68	55.35	---	37.37	177.33	177.84	0.089	0.841	0.0013
248	R	7.71	121.59	55.32	58.41	37.36	27.96	177.83	177.17	0.178	0.985	0.0013
249	A	7.56	119.25	58.36	55.17	28.03	15.57	177.15	180.16	0.274	0.860	0.0026
250	G	7.72	103.14	55.18	46.78	15.58	---	180.15	177.94	1.000	0.674	0.0025
251	E	7.69	124.53	46.78	58.43	---	27.99	178.06	---	0.662	0.695	0.0013
252	Y	7.27	115.89	---	58.19	27.99	37.96	178.18	175.42	0.164	0.783	0.0013
253	G	7.90	108.05	58.15	45.78	37.95	---	175.43	174.60	0.610	0.893	0.0013
254	V	7.95	118.49	45.70	59.46	---	33.96	174.62	175.28	0.497	0.894	0.0013
255	T	8.32	115.43	59.47	---	33.98	69.24	175.28	174.82	0.022	0.098	0.0013
256	P											
257	T	8.06	111.52	66.04	68.24	31.70	68.84	178.25	---	0.978	0.333	0.0026
258	M	7.61	118.13	68.18	55.15	---	32.72	175.31	180.54	0.011	0.032	0.0006
259	A	8.78	127.19	55.13	56.03	32.75	17.57	180.54	179.54	0.979	0.022	0.0026
260	L	8.80	119.03	56.00	58.49	17.59	41.60	179.55	178.69	0.664	0.926	0.0026
261	A	8.32	119.36	58.47	55.30	41.63	16.61	178.68	180.18	0.222	0.816	0.0013
262	T	7.92	113.92	55.24	67.64	16.55	68.74	180.16	---	0.946	0.779	0.0026
263	C	7.95	120.34	67.64	63.80	68.67	27.35	175.34	---	0.652	0.618	0.0026
264	F	9.08	119.63	63.78	58.27	27.26	38.78	175.31	176.84	0.214	0.407	0.0013
265	S	8.00	113.07	58.26	61.98	38.77	---	176.87	---	0.134	0.222	0.0025
266	A	7.85	123.56	61.85	54.89	---	15.98	174.73	180.69	0.615	0.722	0.0013
267	V	7.22	113.47	54.90	65.32	16.02	31.54	180.72	177.93	0.706	0.529	0.0013
268	L	7.28	118.78	65.30	56.32	31.55	41.52	177.97	178.59	0.256	0.894	0.0026
269	A	8.14	119.87	56.31	53.78	41.54	20.40	178.58	---	0.113	0.057	0.0013
270	R	7.10	113.74	53.79	57.66	20.41	28.32	178.56	179.22	0.647	0.006	0.0013
271	W	8.47	119.93	57.66	61.39	---	28.20	179.23	177.73	0.027	0.255	0.0013
272	G	8.65	104.53	61.40	44.27	28.18	---	177.75	174.34	0.891	0.240	0.0025
273	G	7.92	110.84	44.28	46.39	---	---	174.35	175.30	0.812	0.709	0.0024
274	L	7.71	117.55	46.39	54.49	---	42.07	175.30	177.35	0.462	0.633	0.0013
275	T	8.69	109.46	54.48	62.24	42.07	68.93	177.37	175.79	0.404	0.315	0.0026
276	R	7.39	122.14	62.21	54.98	68.97	34.39	175.93	174.83	0.075	0.166	0.0013
277	L	9.10	117.58	54.93	53.59	34.39	45.27	174.83	173.98	0.605	0.659	0.0026
278	L	8.56	124.46	53.48	---	45.27	42.40	174.01	---	0.511	0.788	0.0026

279	L	8.52	129.09	53.23	52.73	42.39	44.93	174.11	175.13	0.672	0.583	0.0013
280	N	6.83	123.00	---	52.57	44.93	38.50	175.11	173.78	0.075	0.069	0.0013
281	I	9.28	126.70	52.47	58.49	38.52	37.87	173.81	174.34	0.253	0.793	0.0013
282	T											
283	L											
284	F	8.23	117.62	52.58	56.89	44.97	39.92	174.52	175.23	0.012	0.020	0.0007
285	D	8.90	123.72	56.73	53.21	39.92	42.85	175.26	173.76	0.001	0.001	0.0013
286	R	8.40	126.30	53.21	53.80	42.83	28.02	173.80	175.30	0.001	0.003	0.0007
287	Q	8.26	128.81	53.85	54.94	28.02	---	175.30	---	0.151	0.120	0.0025
288	P											
289	L											
290	H											
291	P											
292	A	12.18	125.24	64.90	54.10	31.31	19.13	178.16	180.71	0.000	0.000	0.0026
293	V	8.01	116.65	54.11	65.99	19.14	31.99	180.71	177.36	0.876	0.362	0.0013
294	G	7.67	104.53	65.96	46.83	32.01	---	177.37	173.58	0.776	0.485	0.0013
295	A	7.22	120.41	46.83	50.61	---	19.71	173.56	175.98	0.932	0.778	0.0013
296	M	6.83	116.71	50.60	56.46	19.72	35.00	175.98	175.94	0.256	0.246	0.0003
297	L	8.39	124.29	---	53.03	34.98	38.62	175.91	174.24	0.006	0.025	0.0013
298	A											
299	D	7.18	116.95	49.54	---	21.65	---	---	175.60	0.433	0.398	0.0006
300	F	8.52	127.08	53.27	---	---	39.38	175.61	---	0.022	0.326	0.0013
301	T	7.93	117.84	---	64.40	---	68.32	---	---	0.079	0.913	0.0006
302	N	9.61	127.48	64.42	52.62	68.31	41.96	175.39	172.30	0.733	0.168	0.0013
303	I	7.98	123.35	52.63	59.91	41.94	---	172.30	175.11	0.002	0.003	0.0025
304	L	9.28	130.47	59.94	53.35	39.56	45.34	175.10	173.95	0.006	0.002	0.0007
305	L	8.07	121.78	53.38	52.30	45.37	38.79	173.96	173.84	0.067	0.347	0.0007
306	L	8.92	126.13	52.29	53.81	38.80	44.75	173.92	177.07	0.489	0.105	0.0026
307	D	9.18	131.27	53.82	53.72	44.68	39.76	177.08	173.70	0.112	0.084	0.0007
308	T	9.10	125.20	53.72	63.51	39.82	68.20	173.67	174.03	0.316	0.112	0.0026
309	A	10.18	132.32	63.50	52.41	68.16	18.33	174.04	178.45	0.822	0.044	0.0026
310	C	8.53	117.01	52.41	56.63	18.33	30.42	178.45	171.46	0.904	0.266	0.0013
311	D	7.77	116.74	56.60	53.31	30.42	43.71	171.46	175.78	0.183	0.277	0.0013
312	G	8.97	107.11	53.31	45.47	43.70	---	175.74	173.30	0.744	0.952	0.0013
313	D	7.51	117.07	45.47	50.99	---	43.65	173.29	174.50	0.911	0.747	0.0013
314	T	10.03	110.86	50.97	60.72	43.65	70.37	174.49	174.13	0.859	0.011	0.0013
315	V	7.84	119.18	60.72	66.37	70.40	31.56	174.15	177.94	0.226	0.473	0.0013
316	S	8.04	109.24	66.38	61.58	31.64	60.87	177.96	---	0.489	0.439	0.0013
317	N	7.42	122.37	61.57	---	60.89	36.06	176.26	179.86	0.311	0.496	0.0026
318	L	8.83	122.53	55.11	57.55	36.09	40.53	179.86	177.96	0.125	0.387	0.0013

319	A	8.73	120.16	57.55	54.62	40.56	16.71	177.99	179.44	0.167	0.982	0.0013
320	R	8.35	118.22	54.61	59.36	16.71	29.63	179.47	178.74	0.249	0.998	0.0013
321	K	7.92	121.26	59.32	59.31	29.59	31.04	178.75	178.97	0.011	0.958	0.0001
322	N	8.46	117.47	59.40	55.59	31.05	36.63	178.99	176.60	0.253	0.979	0.0013
323	Q	8.86	121.32	55.59	58.77	36.65	28.75	176.61	178.19	0.191	0.805	0.0013
324	L	8.25	121.99	58.77	---	28.79	40.77	178.19	180.45	0.094	0.813	0.0013
325	T	8.59	119.49	58.40	65.64	40.74	69.00	180.47	175.62	0.583	0.494	0.0026
326	F	9.13	120.48	65.65	61.82	69.03	39.83	175.65	176.52	0.762	0.539	0.0026
327	T	8.79	115.41	61.82	67.19	39.85	69.12	176.52	176.59	0.115	0.450	0.0007
328	E	7.35	121.19	67.22	58.74	69.10	29.06	---	179.28	0.181	0.518	0.0007
329	D	8.81	123.80	58.69	57.68	29.15	40.92	179.30	---	0.056	0.318	0.0013
330	W	9.46	121.09	57.67	59.30	40.90	28.21	179.30	179.84	0.106	0.282	0.0026
331	E	7.25	117.95	59.33	57.30	28.21	---	179.85	175.83	0.046	0.347	0.0013
332	H	7.57	113.56	57.26	54.63	---	28.65	175.85	176.44	0.034	0.382	0.0013
333	R	6.94	117.33	54.59	56.26	28.69	27.95	176.45	176.05	0.254	0.657	0.0013
334	H	7.23	115.73	56.24	57.72	27.91	30.03	176.06	174.66	0.090	0.199	0.0026
335	W	6.95	120.95	57.71	57.93	30.06	29.09	174.65	174.51	0.010	0.157	0.0002
336	S	8.50	118.55	58.00	58.81	29.25	63.13	174.52	---	0.060	0.362	0.0013
337	G	8.28	115.86	58.82	46.85	63.13	---	176.63	174.96	0.472	0.500	0.0013
338	V	7.61	114.21	46.83	64.74	---	30.88	174.95	178.46	0.755	0.187	0.0013
339	E	6.80	120.55	64.76	58.33	31.04	27.62	178.52	178.93	0.586	0.747	0.0013
340	L	6.60	120.96	58.31	57.11	27.61	39.02	178.94	178.60	0.380	0.951	0.0026
341	L	8.23	119.15	57.11	57.52	---	40.70	178.59	178.01	0.035	0.878	0.0013
342	R	6.98	116.50	57.57	59.36	40.68	29.14	178.02	179.17	0.248	0.746	0.0013
343	E	7.32	118.31	59.29	57.87	---	28.11	179.18	---	0.018	0.657	0.0006
344	L	8.57	120.08	57.84	---	28.02	39.64	179.37	179.72	0.169	0.940	0.0026
345	K	8.23	117.17	57.43	58.53	---	31.22	179.76	---	0.087	0.949	0.0013
346	R	7.60	119.70	57.23	58.03	31.24	29.37	179.84	177.68	0.544	0.846	0.0026
347	Q	7.80	116.89	58.00	55.23	29.36	28.07	177.77	175.90	0.232	0.999	0.0026
348	Q											
349	R	7.48	115.41	57.10	55.44	26.01	31.69	175.78	175.01	0.585	0.827	0.0026
350	Y	7.99	115.17	55.45	57.35	31.65	36.22	175.01	174.67	0.222	0.375	0.0026
351	P											
352	H	8.11	115.44	64.27	53.84	30.82	31.30	177.59	176.48	0.039	0.046	0.0013
353	G	7.92	108.56	53.86	45.91	31.36	---	176.48	171.97	0.030	0.015	0.0013
354	A	8.02	128.72	45.94	48.62	---	17.89	171.95	173.98	1.000	0.379	0.0013
355	P											
356	V	7.28	121.01	63.96	62.20	---	---	175.30	173.76	0.206	0.501	0.0024
357	V	8.86	130.35	62.21	60.27	31.74	35.59	173.76	---	0.271	0.207	0.0007
358	F	9.31	126.90	60.26	54.42	35.58	41.71	172.91	---	0.130	0.188	0.0013

359	T											
360	S	8.05	117.05	60.49	55.72	---	62.66	173.45	---	0.015	0.094	0.0013
361	N	8.70	128.68	55.70	50.62	62.70	40.64	173.37	174.30	0.256	0.119	0.0013
362	L											
363	G	9.23	110.18	55.97	44.76	40.49	---	177.91	173.33	0.848	0.417	0.0013
364	R	8.10	118.66	44.77	54.43	---	33.01	173.32	173.00	0.368	0.740	0.0013
365	S	8.49	113.59	54.43	56.53	33.00	62.67	173.00	---	0.040	0.084	0.0026
366	L	8.70	128.70	---	---	62.70	40.64	173.37	174.30	0.309	0.287	0.0026
367	Y											
368	S											
369	S	10.22	124.32	---	---	---	64.17	175.69	---	0.015	0.007	0.0013
370	R	7.65	123.55	58.54	56.08	64.22	31.23	174.97	176.63	0.058	0.218	0.0013
371	A	8.66	126.52	56.08	54.24	31.23	17.85	176.63	178.01	0.851	0.812	0.0026
372	E	7.94	116.03	54.25	54.79	17.86	29.43	178.01	174.87	0.925	0.997	0.0026
373	S	8.70	116.82	54.82	53.00	29.46	62.97	174.87	174.25	0.106	0.179	0.0026
374	P											
375	L	8.56	115.38	64.56	57.46	31.67	39.87	178.93	176.10	0.021	0.042	0.0026
376	G	7.70	106.17	57.41	---	39.86	---	176.06	173.41	0.145	0.266	0.0025
377	E	8.66	119.14	42.12	52.53	---	30.13	173.42	175.34	0.892	0.644	0.0013
378	P											
379	E											
380	W											
381	G											
382	I											
383	S											
384	Q											
385	T											
386	P											
387	Q											
388	V	7.29	119.42	60.58	62.68	---	34.15	174.10	---	0.000	0.001	0.0013
389	W	9.83	126.67	62.60	56.64	34.17	31.44	175.48	176.15	0.235	0.176	0.0013
390	I	7.20	114.28	56.63	61.11	31.56	---	176.18	---	0.020	0.109	0.0013
391	D											
392	H											
393	L	8.58	130.23	51.00	53.59	28.82	---	171.33	174.74	0.007	0.000	0.0025
394	A	7.97	127.32	53.59	49.54	44.20	21.39	174.58	175.07	0.825	0.568	0.0026
395	F	8.41	117.30	49.54	55.56	21.37	39.38	175.12	---	0.223	0.573	0.0007
396	E	8.76	119.49	55.57	54.40	39.39	32.72	170.87	175.31	0.055	0.183	0.0013
397	H	8.96	123.34	54.40	55.75	32.69	33.18	175.30	---	0.016	0.060	0.0013
398	H	9.26	126.33	55.76	55.36	32.79	26.65	175.50	174.83	0.329	0.197	0.0026

399	G	8.87	104.60	55.21	45.21	26.86	---	174.89	173.19	0.687	0.646	0.0025
400	E	7.58	117.94	45.21	54.03	---	31.44	173.19	175.70	0.152	0.526	0.0013
401	V	8.13	121.11	54.02	61.39	31.46	32.97	175.73	173.63	0.455	0.491	0.0026
402	W	9.01	130.84	61.42	55.87	32.98	27.95	173.69	173.60	0.117	0.336	0.0003
403	L	8.72	120.26	55.89	52.55	27.99	40.94	173.55	174.94	0.081	0.259	0.0013
404	Q	9.18	121.88	52.54	53.72	41.02	34.27	174.90	175.84	0.220	0.482	0.0026
405	W	9.93	128.32	53.71	55.33	34.23	31.53	175.83	174.13	0.015	0.010	0.0026
406	D	8.88	126.81	55.30	52.88	31.62	---	174.10	176.62	0.079	0.724	0.0013
407	S	8.65	113.83	---	55.50	---	---	---	---	0.016	0.539	0.0012
408	N	8.47	119.91	55.46	50.77	67.73	35.97	171.81	175.53	0.329	0.425	0.0013
409	D	8.44	122.26	50.77	57.22	35.95	39.20	175.55	178.75	0.485	0.760	0.0026
410	A	7.62	119.60	57.22	53.12	39.19	18.35	178.76	---	0.104	0.707	0.0007
411	L	6.68	113.94	53.13	55.12	18.38	40.61	177.63	175.99	0.967	0.271	0.0013
412	F	7.18	114.85	55.08	---	40.63	40.21	176.04	172.60	0.212	0.669	0.0007
413	P											
414	P											
415	A	8.78	121.03	63.99	53.77	30.81	17.39	177.36	178.34	0.301	0.766	0.0013
416	L	7.88	122.52	53.78	59.23	17.39	41.09	178.38	178.75	0.586	0.310	0.0013
417	V	8.15	117.00	59.17	66.83	41.10	30.16	178.75	---	0.419	0.412	0.0007
418	E	7.75	119.99	66.83	59.81	30.19	28.98	177.61	177.99	0.554	0.974	0.0026
419	T	8.05	114.13	59.83	66.13	28.97	68.67	177.98	177.48	0.447	0.861	0.0026
420	L	8.30	123.01	66.11	57.69	68.67	41.39	177.50	177.57	0.059	0.424	0.0007
421	F	8.99	121.05	57.67	61.13	41.39	38.11	177.59	177.67	0.076	0.625	0.0007
422	D	8.40	120.30	61.12	57.43	38.12	40.40	177.69	178.68	0.180	0.964	0.0026
423	A	7.84	121.56	57.45	54.71	40.51	17.47	178.71	179.50	0.134	0.982	0.0013
424	Y	8.48	119.18	54.74	60.65	17.48	38.49	179.65	176.24	0.283	0.972	0.0013
425	C	8.27	116.57	60.64	63.49	38.48	26.33	176.32	176.75	0.828	0.953	0.0026
426	Q	7.91	117.37	63.46	58.53	26.28	27.34	176.74	178.88	0.954	0.934	0.0013
427	L	7.85	122.04	58.51	---	27.35	41.14	---	177.69	0.045	0.964	0.0007
428	I	8.06	117.80	57.69	62.10	41.54	34.51	177.75	179.21	0.510	0.885	0.0026
429	N	8.16	116.91	61.99	56.61	34.43	37.09	179.20	---	0.223	0.872	0.0013
430	Q	8.18	119.96	56.57	59.52	37.00	27.58	---	178.06	0.100	0.959	0.0007
431	L	8.21	118.06	59.52	56.86	27.60	---	178.11	181.16	0.074	0.402	0.0025
432	C	8.25	117.37	---	63.16	39.77	27.14	181.14	176.49	0.130	0.097	0.0013
433	D	7.77	118.45	63.14	55.91	27.18	41.83	176.49	---	0.134	0.317	0.0013
434	D	8.65	121.15	55.90	52.33	41.73	---	176.49	176.11	0.234	0.881	0.0025
435	E	9.16	125.14	52.33	59.27	41.63	28.74	176.12	178.99	0.393	0.955	0.0026
436	S	8.53	116.31	59.26	60.61	28.75	62.83	178.99	---	0.076	0.907	0.0007
437	A	7.64	126.11	60.62	54.08	62.81	18.00	176.88	177.93	0.530	0.441	0.0013
438	W	7.20	113.47	54.09	59.54	18.02	28.42	177.95	176.78	0.705	0.229	0.0013

439	Q	7.59	117.24	59.54	54.03	27.50	28.29	176.77	173.65	0.239	0.646	0.0013
440	K	7.12	119.82	54.01	53.63	28.33	33.78	173.65	173.44	0.082	0.508	0.0003
441	P											
442	F	8.56	119.86	60.73	---	---	38.17	---	---	0.020	0.322	0.0013
443	A	8.81	120.56	62.49	54.25	38.23	18.35	178.73	178.25	0.000	0.000	0.0013
444	D	7.46	115.96	54.29	55.82	18.36	40.34	178.30	177.46	0.002	0.001	0.0026
445	M	7.68	117.29	55.81	56.80	40.35	33.83	177.48	176.74	0.007	0.014	0.0026
446	L	7.89	118.44	56.82	54.92	33.87	42.20	176.77	177.27	0.147	0.308	0.0026
447	E	8.12	119.60	54.94	56.68	42.19	29.28	177.30	176.45	0.063	0.274	0.0013
448	H											
449	H											
450	H											
451	H											
452	H											
453	H											

Assigned amide groups that match the reference assignment are indicated by green shading. Missing amide groups that are present in the reference assignments are indicated by white shading. Amide groups that were assigned to residues that are not assigned in the reference assignments are indicated by orange shading. Amide groups that do not match those in the reference assignments are indicated with red shading. Residues that were not assigned by either BARASA or in the reference assignments were considered matching. Non-amide resonances were not considered when determining assignment accuracy.
The table is included in Excel format in the Source Data file.

Supplementary Table 8 – Assignments of ecTS by BARASA

Index	AA	H	N	CA(i-1)	CA	CB(i-1)	CB	CO(i-1)	CO	Posterior	Likelihood	Prior
1	M											
2	K	9.49	126.41	55.27	60.68	39.06	31.62	178.01	178.315	0.001	0.000	0.005
3	Q	10.24	116.56	60.71	58.98	31.54	28.97	178.33	178.516	0.267	0.028	0.001
4	Y	6.85	118.21	59.01	60.24	28.92	39.68	178.52	175.542	0.969	0.079	0.005
5	L	8.47	120.32	60.27	57.48	39.55	---	175.53	179.577	0.362	0.342	0.004
6	E	8.14	118.55	57.56	59.03	39.38	28.38	179.64	179.546	0.045	0.995	0.001
7	L	7.11	121.85	59.06	57.09	28.40	38.57	179.53	176.892	0.772	0.645	0.005
8	M	7.67	118.35	57.15	60.67	38.53	33.46	176.93	176.743	0.221	0.102	0.001
9	Q	7.86	115.80	60.71	57.32	33.42	28.15	176.74	176.624	0.167	0.347	0.001
10	K	7.70	121.01	57.38	59.25	28.10	31.66	176.60	178.428	0.903	0.761	0.005
11	V	8.12	119.30	59.31	66.46	31.60	29.79	178.47	178.374	0.255	0.908	0.001
12	L	7.37	118.07	66.47	57.59	29.76	40.51	178.34	179.836	0.487	0.946	0.005
13	D	8.38	118.19	57.64	56.50	40.46	40.87	179.85	178.429	0.355	0.928	0.005
14	E	8.71	116.02	56.53	55.06	40.91	30.52	178.45	177.631	0.782	0.612	0.005
15	G	8.18	110.48	55.10	45.74	30.38	---	177.66	173.268	0.554	0.741	0.004
16	T	9.10	120.02	45.74	60.72	---	69.84	173.27	174.228	0.492	0.639	0.002
17	Q	8.67	127.66	60.76	55.85	69.77	27.80	174.24	175.579	1.000	0.581	0.005
18	K	8.72	127.22	55.91	55.29	27.76	35.65	175.57	---	0.998	0.948	0.005
19	N	8.71	122.35	55.32	52.32	35.59	39.65	175.53	174.732	0.944	0.967	0.005
20	D	8.38	121.52	52.40	---	39.58	41.94	174.75	177.859	0.781	0.808	0.005
21	R											
22	T	8.29	110.90	58.20	61.87	28.76	69.72	177.65	175.6	0.969	0.003	0.005
23	G	8.09	110.67	61.90	44.85	69.65	---	175.60	174.227	0.100	0.088	0.004
24	T	7.95	119.10	44.85	62.85	---	68.54	174.24	174.641	0.774	0.649	0.002
25	G	8.70	114.20	62.87	45.18	68.47	---	174.66	173.683	0.862	0.529	0.004
26	T	8.33	112.31	45.20	59.12	---	72.72	173.70	174.114	0.975	0.784	0.002
27	L	8.60	121.15	59.17	53.57	72.67	44.18	174.12	176.816	1.000	0.535	0.005
28	S	8.73	114.53	53.62	57.04	44.14	65.51	176.86	174.157	0.789	0.171	0.005
29	I	8.85	118.95	57.09	59.03	65.46	40.10	174.17	172.712	1.000	0.658	0.005
30	F	8.38	120.63	59.07	54.82	40.08	39.32	172.73	175.111	0.993	0.268	0.005
31	G	7.38	114.91	54.81	44.21	39.27	---	175.14	171.054	0.909	0.033	0.002
32	H	7.42	122.90	44.22	55.85	---	33.59	171.07	172.402	0.621	0.121	0.002
33	Q	6.85	126.85	55.88	54.06	33.50	32.74	172.42	173.155	0.954	0.042	0.005
34	M	9.25	122.65	54.02	---	32.70	36.22	173.16	172.431	0.958	0.511	0.005
35	R	8.40	120.23	53.94	54.19	36.19	33.21	172.44	173.948	0.230	0.566	0.001
36	F	9.27	124.37	54.30	55.93	33.17	38.99	173.96	174.407	0.534	0.849	0.005
37	N	9.16	123.97	55.97	52.51	38.97	36.38	174.42	178.945	1.000	0.339	0.005
38	L	9.47	131.26	52.54	56.76	36.34	39.29	178.98	179.528	1.000	0.013	0.005

39	Q	8.13	118.48	---	57.43	39.32	---	179.61	---	0.028	0.424	0.002
40	D	7.82	119.04	57.52	55.23	26.62	39.79	177.67	175.958	0.367	0.604	0.005
41	G	6.87	106.65	55.28	43.30	39.78	---	175.99	170.436	0.983	0.095	0.004
42	F	8.95	126.48	43.29	55.35	---	39.00	170.39	175.079	0.982	0.046	0.002
43	P											
44	L	8.16	129.42	62.15	51.00	27.45	43.82	171.51	173.1	1.000	0.000	0.005
45	V	7.22	120.56	51.05	64.70	43.79	32.78	173.11	173.753	0.999	0.000	0.005
46	T	9.19	116.71	64.76	62.37	32.93	71.65	173.76	---	0.998	0.002	0.005
47	T											
48	K	7.06	116.90	64.03	55.86	68.00	33.81	173.21	173.932	1.000	0.538	0.005
49	R	6.09	119.23	55.90	57.95	33.76	28.50	173.93	175.747	0.932	0.000	0.005
50	C	8.46	128.38	57.98	---	28.29	29.36	175.75	173.093	0.860	0.126	0.005
51	H											
52	L											
53	R											
54	S	7.47	112.81	---	61.90	27.75	---	180.18	---	0.756	0.342	0.002
55	I											
56	I	8.04	120.73	62.48	65.64	31.78	36.68	176.79	177.254	0.513	0.000	0.002
57	H	7.35	114.85	65.65	61.15	36.64	---	---	177.538	0.500	0.430	0.001
58	E	8.14	118.03	61.02	59.40	30.52	29.16	177.54	175.819	0.383	0.214	0.005
59	L	7.46	120.18	59.47	57.49	29.08	40.36	175.84	178.896	0.345	0.487	0.005
60	L	8.18	117.53	57.60	57.67	40.25	38.71	178.91	180.058	0.146	0.679	0.001
61	W	8.18	123.38	57.76	60.21	38.73	27.83	180.09	178.736	0.230	0.276	0.005
62	F	8.77	120.36	60.27	56.70	27.74	37.32	178.75	180.43	0.791	0.085	0.005
63	L	8.27	115.79	56.77	57.48	37.27	41.68	180.44	178.048	0.875	0.157	0.005
64	Q	7.54	115.63	57.54	55.75	41.61	28.30	178.05	177.808	0.202	0.745	0.001
65	G	8.02	109.13	55.77	44.72	28.24	---	177.82	172.47	0.632	0.267	0.004
66	D	6.61	120.38	44.72	53.02	---	42.16	172.47	175.274	0.845	0.391	0.002
67	T	8.00	113.15	53.09	60.66	42.13	68.38	175.30	172.109	1.000	0.278	0.005
68	N	8.43	122.47	60.68	51.89	68.33	42.02	172.13	177.626	0.881	0.065	0.005
69	I	9.03	121.46	51.91	63.05	41.96	36.96	177.68	175.976	1.000	0.245	0.005
70	A	8.93	128.62	63.08	56.60	36.90	16.67	176.01	180.017	0.964	0.080	0.005
71	Y	9.35	118.44	56.64	60.93	16.61	38.80	180.02	178.039	0.999	0.017	0.005
72	L	6.59	117.37	60.97	56.83	38.80	38.09	178.04	180.094	0.956	0.491	0.005
73	H	8.43	121.42	56.89	59.20	---	28.41	180.09	179.563	0.132	0.620	0.002
74	E	8.41	122.20	---	58.20	---	28.20	179.57	176.282	0.069	0.475	0.001
75	N	6.67	114.50	58.26	52.47	28.06	38.21	176.30	172.874	0.979	0.677	0.005
76	N	7.76	115.52	52.48	54.01	38.07	36.18	172.88	173.403	1.000	0.974	0.005
77	V	8.13	120.29	54.05	60.47	36.17	---	173.41	177.048	0.786	0.871	0.002
78	T	8.57	116.82	61.75	---	31.06	68.75	177.06	---	0.286	0.747	0.002

79	I	6.49	114.78	61.76	63.93	68.72	37.41	177.47	176.39	1.000	0.006	0.005
80	W	8.49	121.45	63.96	58.44	37.34	---	176.37	---	0.671	0.162	0.004
81	D	7.62	121.83	58.45	57.91	29.74	39.13	176.36	179.163	0.225	0.317	0.005
82	E	8.49	116.33	57.92	57.74	39.06	27.72	179.17	176.439	0.108	0.784	0.001
83	W	7.14	116.75	57.67	---	27.68	30.36	176.44	175.028	0.788	0.832	0.005
84	A	7.02	121.92	57.55	51.41	30.28	19.73	175.06	177.371	0.993	0.777	0.005
85	D	8.27	120.97	51.46	52.11	19.68	39.97	177.39	178.596	0.992	0.929	0.005
86	E	9.04	117.77	52.13	58.44	39.92	27.88	178.62	177.008	0.856	1.000	0.005
87	N	8.29	117.36	58.48	52.44	27.86	39.04	177.02	175.474	0.648	0.973	0.005
88	G	8.31	109.62	52.48	45.07	39.00	---	175.49	173.059	0.587	0.875	0.004
89	D	8.51	121.59	45.07	55.58	---	42.44	173.07	177.713	0.905	0.274	0.002
90	L	7.97	121.32	55.63	54.11	42.44	44.31	177.73	176.992	0.980	0.024	0.005
91	G	7.90	107.19	54.14	43.99	44.29	---	177.04	173.719	0.982	0.254	0.004
92	P											
93	V	8.41	114.56	62.67	60.18	27.92	31.19	175.29	174.165	0.958	0.007	0.005
94	Y	6.22	110.74	60.21	62.31	31.13	39.64	174.18	174.975	1.000	0.000	0.005
95	G	7.61	133.31	62.33	47.68	39.63	---	175.05	---	0.000	0.000	0.004
96	K	7.01	119.49	47.62	57.46	---	31.03	175.16	178.213	0.979	0.491	0.002
97	Q	7.01	113.39	57.53	56.29	31.01	26.33	178.23	179.383	0.939	0.164	0.005
98	W	9.04	116.66	56.34	59.52	26.17	28.95	179.42	178.377	0.983	0.426	0.005
99	R	7.63	112.00	59.57	53.29	28.75	---	178.39	176.791	0.588	0.439	0.004
100	A	8.62	126.94	53.31	51.23	28.43	16.94	176.81	174.713	0.968	0.022	0.005
101	W	8.47	126.75	51.31	55.70	16.89	26.56	174.72	176.586	0.622	0.033	0.001
102	P											
103	T	8.50	114.18	62.23	59.12	31.69	67.31	177.29	175.081	0.955	0.935	0.005
104	P											
105	D	7.92	112.47	64.56	52.33	30.52	39.04	176.13	176.238	0.177	0.975	0.001
106	G	7.60	107.33	52.36	45.73	39.03	---	176.28	174.716	0.511	0.967	0.004
107	R	7.33	119.52	45.73	54.29	---	30.50	174.73	175.375	0.552	0.627	0.002
108	H	8.12	118.35	54.33	55.61	30.43	32.11	175.38	174.922	0.492	0.298	0.005
109	I	9.55	124.96	55.65	59.86	32.06	38.48	174.93	174.197	0.628	0.111	0.005
110	D	8.64	126.19	59.90	51.44	38.38	38.83	174.21	178.063	0.999	0.112	0.005
111	Q	8.46	126.70	51.34	59.16	38.95	31.59	178.09	177.792	0.457	0.003	0.002
112	I	7.99	119.01	59.25	61.01	31.57	33.90	177.79	177.03	0.009	0.005	0.001
113	T	7.71	118.00	61.06	67.27	33.90	---	177.04	---	0.449	0.064	0.001
114	T	7.74	117.57	67.26	---	68.24	68.07	176.50	---	0.176	0.861	0.001
115	V	7.97	119.92	67.57	65.02	68.10	30.11	176.14	177.582	1.000	0.936	0.005
116	L	7.80	121.94	65.05	57.92	30.02	40.85	177.59	178.565	0.404	0.617	0.005
117	N	7.73	116.04	57.92	55.96	40.81	37.55	178.61	178.52	0.117	0.718	0.001
118	Q	8.43	121.51	55.99	58.07	37.63	29.63	178.52	178.141	0.630	0.410	0.005

119	L	8.68	118.96	59.25	57.56	29.59	41.55	178.09	---	0.035	0.075	0.005
120	K	7.30	112.95	57.65	58.12	41.58	32.72	177.97	178.341	0.964	0.802	0.005
121	N	8.03	114.31	58.15	53.95	32.65	40.37	178.35	174.675	0.999	0.952	0.005
122	D	9.22	123.44	54.09	52.21	40.43	41.13	174.75	172.535	0.160	0.002	0.005
123	P											
124	D	7.93	114.12	64.54	54.32	29.98	40.11	175.69	176.865	0.441	0.538	0.005
125	S	7.92	116.23	54.35	58.64	40.08	62.37	176.86	176.417	0.813	0.542	0.005
126	R	9.24	127.68	58.68	56.05	62.33	28.76	176.57	---	1.000	0.366	0.005
127	R	8.64	119.36	56.08	53.56	28.57	28.99	176.69	174.723	0.364	0.548	0.002
128	I	8.81	125.76	53.61	61.17	28.99	33.69	174.66	---	0.834	0.007	0.005
129	I	7.75	127.70	61.23	58.85	33.62	43.28	174.53	175.292	1.000	0.030	0.005
130	V	8.02	127.57	58.90	60.06	43.25	31.24	175.32	173.779	0.313	0.021	0.005
131	S	9.10	117.20	60.09	54.35	31.19	65.87	173.79	174.814	0.999	0.030	0.005
132	A	9.15	127.66	54.38	50.64	65.76	18.78	174.86	176.176	1.000	0.526	0.005
133	W	8.90	123.34	50.68	55.87	18.74	26.47	176.22	174.291	0.652	0.518	0.005
134	N	6.94	124.61	55.89	50.87	26.42	33.44	174.46	---	1.000	0.011	0.005
135	V	5.82	123.54	50.94	64.37	33.38	30.33	174.63	175.583	1.000	0.000	0.005
136	G											
137	E	6.97	115.73	45.63	55.20	---	30.61	172.95	176.068	0.825	0.443	0.002
138	L	6.69	121.76	55.25	57.63	30.47	41.39	176.11	179.005	0.963	0.606	0.005
139	D	8.17	114.34	57.65	55.14	41.31	38.90	179.00	176.684	0.346	0.778	0.002
140	K	7.26	117.30	55.29	55.03	38.83	32.40	176.69	177.192	0.207	0.975	0.001
141	M	7.16	119.71	55.03	56.10	32.33	32.63	177.23	176.798	0.493	0.769	0.002
142	A	8.48	124.07	56.17	54.38	32.64	19.31	176.81	176.8	0.245	0.673	0.001
143	L	6.49	111.95	54.42	53.16	19.23	43.70	176.79	174.784	1.000	0.796	0.005
144	A	8.15	127.28	53.20	49.37	43.65	15.49	174.79	176.338	1.000	0.387	0.005
145	P											
146	C											
147	H											
148	A	7.88	126.39	53.69	55.46	29.25	20.33	174.64	175.612	0.997	0.222	0.005
149	F											
150	F	8.74	119.93	56.48	54.63	41.05	39.97	171.86	---	0.957	0.344	0.005
151	Q	8.84	120.45	54.66	52.13	39.94	31.83	171.78	174.404	0.989	0.559	0.005
152	F	9.10	124.05	52.16	56.75	31.75	41.65	174.42	172.974	0.450	0.355	0.005
153	Y	7.96	121.86	56.80	55.95	41.59	39.90	172.99	172.225	0.087	0.076	0.005
154	V	8.20	127.96	55.99	59.68	39.85	32.62	172.23	174.591	0.552	0.500	0.005
155	A	7.87	125.21	59.71	51.45	32.60	21.33	174.59	177.338	0.759	0.598	0.005
156	D	9.50	123.87	51.48	54.61	21.32	39.31	177.35	175.808	0.999	0.682	0.005
157	G	8.46	132.34	54.66	45.36	39.25	---	175.85	173.509	0.000	0.000	0.004
158	K	7.89	119.75	45.35	53.83	---	33.84	173.53	175.699	0.933	0.850	0.002

159	L	9.04	126.00	53.90	53.59	33.78	43.41	175.72	175.076	0.725	0.969	0.005
160	S	9.81	122.39	53.52	57.09	43.36	66.14	175.08	173.057	0.687	0.966	0.005
161	C	8.99	120.85	57.15	56.13	66.09	32.10	173.09	172.111	0.369	0.370	0.005
162	Q	9.70	129.67	56.17	52.31	31.85	---	172.13	172.543	0.890	0.118	0.004
163	L	8.22	128.43	52.24	---	31.51	45.95	172.57	172.872	1.000	0.166	0.005
164	Y	8.49	124.11	52.13	56.68	46.02	39.58	172.90	173.405	0.996	0.127	0.005
165	Q	8.30	131.62	56.73	52.96	39.48	27.27	173.46	174.361	1.000	0.049	0.005
166	R	8.01	123.27	53.01	57.26	27.22	28.74	174.43	---	0.435	0.022	0.005
167	S	6.89	115.49	57.30	55.74	28.70	64.12	174.53	173.709	0.431	0.153	0.005
168	C	8.89	127.79	55.78	56.80	64.04	30.33	173.74	170.443	0.996	0.222	0.005
169	D	8.49	130.53	56.87	51.97	30.27	40.27	170.46	177.546	1.000	0.321	0.005
170	V	9.19	125.56	52.01	65.29	40.19	31.32	177.56	176.603	0.985	0.820	0.005
171	F	8.39	119.67	65.32	61.33	31.24	39.36	176.60	173.699	1.000	0.762	0.005
172	L	7.86	115.00	61.37	54.97	39.35	42.71	173.68	179.853	1.000	0.248	0.005
173	G	7.76	107.31	54.96	46.69	42.64	---	179.86	175.418	0.996	0.614	0.002
174	L	7.77	121.91	46.75	---	---	38.42	175.44	173.187	0.129	0.067	0.000
175	P											
176	F	6.67	113.38	65.64	58.96	30.28	37.48	179.20	176.566	0.999	0.090	0.005
177	N	7.91	118.91	59.05	56.27	37.42	39.76	176.59	---	0.081	0.080	0.005
178	I	7.96	115.61	56.33	64.48	39.82	37.38	176.60	176.013	1.000	0.038	0.005
179	A	6.52	118.12	64.49	54.41	37.30	17.96	176.04	178.132	0.998	0.347	0.005
180	S	8.26	112.03	54.46	62.48	17.89	---	178.12	---	0.809	0.482	0.004
181	Y	6.89	114.65	62.53	62.26	---	38.72	175.45	177.666	0.096	0.262	0.001
182	A	7.89	121.91	62.27	55.13	38.70	17.64	177.72	179.661	0.527	0.797	0.005
183	L	8.00	119.47	55.19	58.30	17.53	40.43	179.67	178.163	0.989	0.952	0.005
184	L	7.36	119.49	58.30	57.49	40.43	39.84	178.12	179.133	0.260	0.903	0.002
185	V	8.32	119.15	57.54	67.59	39.58	29.85	179.06	177.882	1.000	0.748	0.005
186	H	7.93	118.20	67.64	---	29.77	27.96	177.90	179.318	0.871	0.474	0.001
187	M	7.48	114.68	---	---	27.98	---	---	---	0.029	0.345	0.004
188	M	8.64	118.35	59.24	57.30	34.88	30.34	177.29	178.167	0.858	0.197	0.005
189	A	8.79	120.95	57.35	55.44	30.28	16.69	178.17	179.44	1.000	0.864	0.005
190	Q	7.89	117.77	55.49	58.71	16.57	27.38	179.44	180.238	1.000	0.627	0.005
191	Q	8.08	115.23	58.75	57.00	27.39	27.93	180.26	177.169	0.623	0.653	0.005
192	C	7.48	114.64	57.05	58.57	27.96	27.49	177.19	172.235	0.510	0.942	0.002
193	D	7.51	117.87	58.54	55.56	27.41	38.45	172.25	174.314	0.997	0.802	0.005
194	L	8.32	117.88	55.61	52.64	38.40	45.11	174.32	176.828	0.999	0.694	0.005
195	E	7.84	118.55	52.69	54.24	45.10	30.69	176.83	177.013	0.245	0.118	0.001
196	V	8.45	114.36	54.27	62.58	30.70	31.56	177.05	175.355	1.000	0.112	0.005
197	G	8.35	113.22	62.60	45.02	31.58	---	175.38	172.007	0.529	0.238	0.004
198	D	8.53	125.20	45.01	52.80	---	42.37	172.02	176.278	0.911	0.817	0.002

199	F	9.51	120.25	52.84	55.26	42.32	40.59	176.27	173.236	0.996	0.964	0.005
200	V	9.28	130.85	55.31	60.16	40.53	31.53	173.26	172.043	0.998	0.455	0.005
201	W	8.92	129.25	60.19	58.12	31.45	30.99	172.06	173.753	1.000	0.119	0.005
202	T	7.71	125.49	58.16	60.63	30.82	69.04	173.77	170.986	1.000	0.081	0.005
203	G	7.69	111.52	60.66	43.26	68.97	---	171.02	174.843	0.999	0.844	0.004
204	G	7.84	107.46	43.25	44.72	---	---	174.85	174.637	0.259	0.490	0.001
205	D	7.78	129.54	44.71	55.75	---	40.82	174.59	174.232	0.570	0.006	0.002
206	T	9.10	130.67	55.77	63.29	40.78	68.73	174.26	172.601	1.000	0.002	0.005
207	H	8.70	121.32	63.32	53.18	68.64	32.41	172.58	---	0.992	0.562	0.005
208	L	8.70	120.09	53.22	51.72	32.37	46.23	172.53	177.374	1.000	0.484	0.005
209	Y	9.71	124.49	51.70	59.80	46.14	36.79	177.38	178.61	1.000	0.077	0.002
210	S	8.63	118.70	59.84	61.28	36.75	62.09	178.65	---	1.000	0.425	0.005
211	N	8.25	116.51	61.22	53.26	62.12	35.95	175.86	174.925	0.999	0.956	0.002
212	H	7.76	117.97	53.40	53.15	35.87	30.95	174.93	177.718	0.146	0.246	0.001
213	M	7.49	123.11	53.13	58.56	30.82	31.44	177.77	---	0.947	0.074	0.005
214	D	9.20	121.65	58.57	57.44	31.28	38.77	177.84	179.423	0.955	0.796	0.005
215	Q	8.60	122.11	57.51	60.02	38.69	26.32	179.44	177.165	0.154	0.047	0.005
216	T	7.72	117.64	60.05	67.20	26.31	---	177.18	176.54	0.725	0.047	0.002
217	H	8.01	119.06	67.20	59.92	67.67	29.03	175.90	178.689	1.000	0.619	0.005
218	L	7.61	120.27	59.96	57.39	28.96	40.53	178.70	179.994	0.310	0.781	0.005
219	Q	8.45	124.67	57.42	60.80	40.50	26.16	180.01	177.501	0.257	0.027	0.005
220	L	8.18	114.13	60.84	56.33	26.15	41.67	177.50	177.913	0.774	0.056	0.002
221	S	7.50	114.41	56.37	58.54	41.64	63.64	177.91	173.756	0.996	0.723	0.005
222	R	7.53	124.17	58.61	55.02	63.59	30.46	173.74	174.668	0.722	0.309	0.005
223	E	8.46	122.82	55.07	52.99	30.31	29.52	174.82	---	0.565	0.792	0.005
224	P											
225	R	8.82	126.58	62.98	53.54	31.25	28.85	176.04	172.809	0.945	0.058	0.005
226	P											
227	L	8.22	122.33	62.98	53.10	30.75	40.03	175.79	176.787	0.571	0.408	0.005
228	P											
229	K	8.68	117.10	61.65	53.79	33.22	34.58	173.11	175.044	1.000	0.569	0.005
230	L											
231	I	9.17	130.44	53.44	59.19	41.69	38.48	175.03	174.487	0.964	0.755	0.005
232	I	8.18	125.96	59.24	60.39	38.46	36.50	174.50	178.571	1.000	0.373	0.005
233	K	9.15	126.70	60.43	57.92	36.47	32.17	178.58	174.95	1.000	0.221	0.005
234	R	7.26	116.52	57.97	53.64	32.07	32.46	174.99	172.344	0.999	0.651	0.005
235	K	8.29	122.57	53.67	52.17	32.39	---	172.36	173.769	0.404	0.631	0.004
236	P											
237	E	9.02	118.66	61.50	57.67	31.96	28.96	176.04	175.298	0.991	0.906	0.005
238	S	7.07	110.25	57.67	56.33	28.91	66.23	175.31	174.778	0.514	0.475	0.001

239	I	8.56	121.12	56.39	59.79	66.19	38.22	174.78	172.752	0.000	0.000	0.005
240	F	7.46	118.48	59.84	56.92	38.04	37.68	172.78	175.968	0.347	0.000	0.005
241	D	7.29	118.91	56.98	53.57	37.62	43.44	176.07	---	0.137	0.060	0.005
242	Y	8.56	120.35	53.59	59.95	43.45	38.25	176.22	174.993	0.013	0.002	0.005
243	R	9.07	119.86	59.97	53.15	38.25	32.69	175.01	176.545	0.914	0.196	0.005
244	F	9.25	123.44	53.18	61.74	32.55	39.15	176.60	---	1.000	0.931	0.005
245	E	9.11	114.01	61.78	57.55	39.06	27.29	176.70	176.197	0.987	0.833	0.005
246	D	7.85	119.62	57.60	55.85	27.21	40.06	176.21	174.387	0.673	0.978	0.005
247	F	6.79	115.57	55.89	56.15	40.08	39.58	174.40	175.098	0.247	0.839	0.001
248	E	8.96	123.67	56.21	54.55	40.15	32.69	175.11	173.197	0.861	0.950	0.005
249	I	8.45	123.17	54.57	57.51	32.68	37.98	173.21	175.145	0.786	0.905	0.005
250	E	9.24	129.46	57.54	54.33	37.91	32.04	175.08	176.528	0.914	0.938	0.005
251	G											
252	Y	8.64	121.85	45.95	55.24	---	37.23	175.04	173.357	0.672	0.184	0.002
253	D	8.37	130.23	55.30	50.55	37.17	40.31	173.38	171.745	1.000	0.412	0.005
254	P											
255	H	8.33	119.11	62.16	55.83	31.88	31.18	178.53	172.262	0.593	0.004	0.002
256	P											
257	G	8.81	108.34	62.99	45.44	31.23	---	177.28	173.301	0.732	0.855	0.004
258	I	8.22	124.00	45.43	60.42	---	40.15	173.32	174.729	0.850	0.693	0.002
259	K	8.59	128.70	60.50	55.85	40.12	32.21	174.75	175.947	0.895	0.866	0.005
260	A	8.42	127.16	55.88	49.53	32.10	18.08	175.94	174.933	0.983	0.799	0.005
261	P											
262	V	8.09	120.72	62.29	61.47	31.21	31.76	176.53	175.724	0.214	0.033	0.005
263	A	8.25	129.11	61.53	51.94	31.89	18.25	175.74	176.266	0.297	0.062	0.005
264	I	7.53	125.49	51.89	62.30	18.17	38.69	176.28	181.034	1.000	0.000	0.005

Assigned amide groups that match the reference assignment are indicated by green shading. Missing amide groups that are present in the reference assignments are indicated by white shading. Amide groups that were assigned to residues that are not assigned in the reference assignments are indicated by orange shading. Amide groups that do not match those in the reference assignments are indicated with red shading. Amide groups indicated with blue shading are matching assignments that were confirmed manually, but were not assigned in the original reference assignment set (BMRB:19082[ref 14]. Residues that were not assigned by either BARASA or in the reference assignments were considered matching. Non-amide resonances were not considered when determining assignment accuracy.
The table is included in Excel format in the Source Data file.

Supplementary Table 9 – Assignments of V5Domain by BARASA

Index	AA	H	N	CA(i-1)	CA	CB(i-1)	CB	CO(i-1)	CO	Posterior	Likelihood	Prior
1	E											
2	N											
3	R	8.57	122.73	53.29	56.12	38.90	30.97	174.86	176.01	1.000	0.883	0.020
4	E	8.46	122.22	56.16	---	30.90	30.36	176.02	176.26	1.000	0.985	0.010
5	I	8.20	122.44	56.57	60.90	30.30	38.74	176.30	176.06	1.000	0.988	0.010
6	Q	8.44	125.89	60.93	53.37	38.73	29.05	176.05	173.35	1.000	0.981	0.020
7	P											
8	P											
9	F	8.14	120.23	62.99	57.51	31.95	39.72	176.44	175.06	1.000	0.961	0.020
10	K	8.05	125.49	57.56	53.53	39.71	33.16	175.07	173.50	1.000	0.710	0.020
11	P											
12	K	8.45	122.02	62.84	---	32.25	33.06	176.67	---	0.512	0.960	0.020
13	V	8.18	121.46	56.41	61.93	33.09	---	176.69	175.98	1.000	0.781	0.018
14	S	8.44	119.81	61.95	58.24	33.08	64.05	176.00	174.87	1.000	0.940	0.020
15	G	8.45	111.03	58.29	45.31	64.10	---	174.89	174.11	1.000	0.986	0.018
16	K	8.29	121.01	45.30	56.47	---	33.06	174.12	177.25	1.000	0.982	0.005
17	G	8.54	110.57	56.54	45.35	33.01	---	177.26	174.01	1.000	0.979	0.009
18	A	8.17	123.78	45.34	52.62	---	19.42	174.02	177.91	1.000	0.995	0.005
19	E	8.50	119.70	52.64	56.87	19.37	30.04	177.92	176.16	1.000	0.996	0.020
20	N	8.27	118.84	56.90	53.25	30.02	39.04	176.17	175.00	0.537	0.977	0.020
21	F	8.13	120.68	53.28	58.08	39.02	39.44	175.02	175.58	1.000	0.976	0.020
22	D	8.26	121.26	58.09	54.67	39.49	41.18	175.60	176.52	1.000	0.647	0.020
23	K	8.00	120.66	54.67	56.82	41.17	32.63	176.48	---	1.000	0.616	0.020
24	F	8.09	119.33	56.84	57.97	32.64	39.40	176.42	175.57	1.000	0.979	0.020
25	F	8.00	120.61	58.03	---	39.41	39.69	175.69	---	0.930	0.664	0.020
26	T	8.05	115.81	58.03	61.88	39.68	69.76	175.77	174.29	1.000	0.472	0.020
27	R	8.25	123.29	61.92	56.40	69.78	30.67	174.29	176.66	1.000	0.963	0.020
28	G	8.38	109.80	56.43	45.13	30.63	---	176.67	173.64	0.583	0.926	0.018
29	Q	8.12	120.70	45.13	53.52	---	29.14	173.66	175.02	1.000	0.176	0.005
30	P											
31	V	8.23	120.93	63.14	---	32.13	---	176.56	175.94	0.237	0.940	0.009
32	L	8.31	126.60	62.28	54.84	32.80	42.58	175.98	176.78	1.000	1.000	0.020
33	E	8.31	123.19	54.86	54.21	42.58	29.80	176.81	173.81	1.000	0.917	0.020
34	P											
35	P											
36	D	8.32	119.32	63.22	54.53	32.06	41.10	176.82	176.30	1.000	0.884	0.020
37	Q	8.19	119.78	54.54	55.94	41.09	29.58	176.33	175.77	0.659	0.960	0.020
38	L	8.19	122.91	---	---	29.55	42.35	175.77	177.14	1.000	0.987	0.020

39	V	8.06	122.03	55.49	62.50	42.34	32.73	177.15	176.06	1.000	0.972	0.020
40	I	8.17	125.16	62.51	60.95	32.71	38.55	176.08	175.80	0.932	0.920	0.010
41	A	8.30	127.86	60.97	52.47	38.54	19.59	175.79	177.14	1.000	0.752	0.020
42	N	8.34	118.16	52.50	---	19.54	38.92	177.16	175.23	1.000	0.985	0.020
43	I	8.04	120.69	53.25	61.37	38.90	---	175.25	175.77	1.000	0.825	0.018
44	D	8.41	123.87	61.38	54.41	39.03	41.29	175.78	176.24	0.999	0.900	0.020
45	Q	8.30	121.30	54.42	56.09	41.29	29.33	176.22	---	0.480	0.937	0.020
46	S	8.39	116.87	56.07	59.07	29.32	63.75	176.19	174.36	0.568	1.000	0.020
47	D	8.26	121.68	59.10	54.42	63.80	41.00	174.38	175.92	1.000	0.988	0.020
48	F	8.03	119.99	54.43	57.84	41.00	39.58	175.86	---	1.000	0.996	0.020
49	E	8.30	122.59	57.84	56.91	39.58	30.25	175.83	176.63	0.660	0.997	0.020
50	G	7.93	109.22	56.89	45.36	30.19	---	176.64	174.00	0.973	0.559	0.018
51	F	8.02	119.87	45.36	58.04	---	39.72	174.01	---	1.000	0.969	0.005
52	E	8.42	121.89	58.03	56.57	39.70	30.28	---	175.74	0.327	0.931	0.010
53	Y	8.05	121.00	56.63	58.03	30.22	38.86	175.71	175.41	0.954	0.996	0.020
54	V	7.81	122.53	58.05	61.83	38.84	33.12	175.42	174.90	0.702	0.952	0.020
55	N	8.34	123.47	---	51.18	33.11	39.02	174.92	173.87	1.000	0.149	0.005
56	P											
57	Q	8.26	118.21	63.78	56.26	32.15	29.16	177.04	175.84	1.000	0.670	0.020
58	F	7.94	119.68	56.30	57.23	29.14	39.58	175.85	175.26	0.976	0.363	0.020
59	V	7.79	121.75	57.26	62.13	39.58	32.87	175.25	---	0.810	0.907	0.020
60	H	8.35	123.39	---	53.65	---	29.37	175.20	172.73	0.996	0.927	0.005
61	P											
62	I	8.39	121.56	63.20	61.23	32.26	38.68	176.77	176.27	1.000	0.993	0.020
63	L	8.33	126.54	61.25	54.97	38.67	42.40	176.31	177.14	1.000	0.998	0.020
64	Q	8.41	121.88	54.98	---	42.39	29.46	177.17	175.94	0.962	0.998	0.010
65	S	8.28	116.84	55.97	58.27	29.54	63.80	175.90	174.15	0.562	0.998	0.020
66	A	8.34	126.08	58.30	52.51	63.82	19.46	174.15	177.56	1.000	0.991	0.020
67	V	8.06	118.69	52.54	62.29	19.38	32.84	177.62	176.08	1.000	0.938	0.020
68	Q*	8.58	119.37	62.30	51.90	32.83	30.38	176.10	180.62	0.000	0.000	0.005

Assigned amide groups that match the reference assignment are indicated by green shading. Missing amide groups that are present in the reference assignments are indicated by white shading. Amide groups that were assigned to residues that are not assigned in the reference assignments are indicated by orange shading. Amide groups that do not match those in the reference assignments are indicated with red shading. Amide groups shown in blue shading are matching assignments that were confirmed manually, but were not present in the BMRB 18927[ref 15]. Residues that were not assigned by either BARASA or in the reference assignments were considered matching. Non-amide resonances were not considered when determining assignment accuracy.

*Non-canonical amino acid due to cyanogen bromide cleavage, sequence-specific random coil shifts were calculated assuming the residue was a glutamine.

The table is included in Excel format in the Source Data file.

Supplementary Table 10 – Assignments of hIDD by BARASA										
**Index**	**AA**	**H**	**N**	**CA(i-1)**	**CA**	**CO(i-1)**	**CO**	**Posterior**	**Likelihood**	**Prior**
1	G									
2	R									
3	Q	8.58	122.09	56.18	55.79	175.92	175.46	0.481	0.848	0.009
4	Y	8.37	121.86	55.81	57.83	175.46	175.75	0.818	0.976	0.009
5	L	8.29	123.91	57.81	55.13	175.75	176.84	0.850	0.895	0.009
6	D	8.25	120.51	55.13	54.72	176.86	176.60	0.170	0.984	0.009
7	G	8.28	108.91	54.73	45.42	176.59	174.07	0.520	0.999	0.009
8	Y	8.00	120.09	45.43	58.00	174.08	175.36	0.307	0.809	0.009
9	D	8.38	122.16	58.00	54.21	175.36	175.80	0.447	0.836	0.009
10	R	8.05	121.14	54.09	56.06	175.81	175.95	0.140	0.805	0.002
11	D	8.43	121.39	56.08	54.42	175.98	176.00	0.041	0.832	0.002
12	D	8.26	120.80	54.39	54.29	176.00	176.34	0.032	0.927	0.002
13	K	8.21	120.99	54.31	56.37	176.32	176.67	0.102	0.933	0.009
14	E	8.35	121.35	56.39	56.33	176.66	175.86	0.021	0.783	0.002
15	A	8.27	126.18	56.30	50.47	175.87	175.42	0.987	0.645	0.009
16	P									
17	D	8.45	120.53	63.03	54.30	176.74	176.52	0.997	0.988	0.009
18	S	8.22	116.02	54.31	58.69	176.52	174.48	0.671	0.992	0.009
19	D	8.38	122.25	58.70	54.31	174.49	176.22	0.861	0.931	0.009
20	A	8.07	123.98	54.42	52.78	176.18	178.02	0.368	0.994	0.009
21	E	8.37	120.05	52.77	56.93	178.02	177.12	0.974	0.995	0.009
22	G	8.36	109.77	56.94	45.26	177.14	173.62	0.235	0.995	0.009
23	Y	8.01	120.99	45.24	58.30	173.61	175.40	0.457	0.598	0.009
24	V	7.96	125.13	58.30	61.68	175.40	175.14	1.000	0.064	0.009
25	E	8.40	125.72	61.69	56.77	175.14	176.79	0.934	0.652	0.009
26	G	8.47	110.80	56.77	45.40	176.80	174.07	0.212	0.983	0.009
27	L	8.11	121.82	45.39	55.05	174.07	177.20	0.960	0.905	0.009
28	D	8.40	121.09	55.04	54.29	177.22	175.86	0.365	0.895	0.009
29	D	8.26	120.82	54.25	54.36	175.88	176.21	0.039	0.897	0.002
30	E	8.37	121.19	54.35	56.44	176.21	176.43	0.052	0.531	0.009
31	E	8.45	122.45	56.43	56.32	176.43	176.34	0.001	0.216	0.001
32	E	8.51	122.85	56.28	56.25	176.34	176.06	0.008	0.317	0.002
33	D	8.52	122.78	56.25	54.22	176.06	176.10	0.020	0.286	0.002
34	E	8.49	122.10	54.23	56.25	176.13	176.14	0.022	0.370	0.002
35	D									
36	E	8.43	122.04	54.32	56.28	176.41	176.35	0.018	0.538	0.002
37	E	8.46	122.89	56.30	56.39	176.36	176.05	0.002	0.115	0.002
38	E	8.41	122.81	56.40	56.13	176.03	175.77	0.016	0.078	0.009

39	Y	8.36	122.80	56.12	57.62	175.78	175.01	0.159	0.165	0.009
40	D	8.33	124.11	57.61	53.60	175.02	176.08	0.663	0.150	0.009
41	E	8.46	123.13	53.61	57.14	176.08	176.51	0.258	0.355	0.009
42	D	8.39	120.86	57.14	54.53	176.51	176.05	0.090	0.976	0.009
43	A	7.94	124.03	54.52	52.50	176.04	177.63	0.293	0.579	0.009
44	Q	8.27	119.88	52.48	55.58	177.62	175.84	0.996	0.963	0.009
45	V	8.27	123.07	55.58	62.37	175.84	175.97	0.236	0.997	0.002
46	V	8.37	125.87	62.36	62.04	175.98	175.97	0.250	0.996	0.002
47	E	8.59	126.03	62.04	56.23	175.97	175.94	0.191	0.912	0.002
48	D	8.43	122.25	56.24	54.37	175.92	176.07	0.047	0.908	0.002
49	E	8.45	121.75	54.36	56.35	176.07	176.34	0.077	0.547	0.009
50	E									
51	D									
52	E	8.47	122.04	54.23	56.22	176.11	176.11	0.021	0.343	0.002
53	D	8.50	122.36	56.26	54.32	176.13	176.69	0.026	0.159	0.009
54	E									
55	E	8.54	123.29	56.33	56.26	176.37	176.38	0.001	0.128	0.001
56	E	8.52	122.95	56.25	56.27	176.36	176.36	0.002	0.400	0.001
57	E	8.61	123.49	56.27	56.66	176.38	176.91	0.066	0.630	0.009
58	G	8.50	110.67	56.64	45.03	176.89	173.76	0.184	0.723	0.009
59	E	8.37	120.73	45.03	56.07	173.78	176.49	0.106	0.364	0.009
60	E	8.59	122.83	56.06	56.40	176.49	176.39	0.008	0.315	0.002
61	E	8.53	123.03	56.36	56.35	176.38	176.00	0.014	0.620	0.002
62	D	8.56	122.90	56.36	54.14	175.99	176.24	0.181	0.589	0.009
63	V	8.25	120.83	54.09	61.70	176.24	176.43	0.220	0.410	0.002
64	S	8.54	120.20	61.75	58.82	176.44	175.15	1.000	0.561	0.009
65	G	8.51	111.27	58.82	45.27	175.16	174.09	0.982	0.998	0.009
66	E	8.29	120.48	45.28	56.29	174.07	176.52	0.140	0.808	0.009
67	E	8.54	122.11	56.36	56.31	176.53	176.42	0.002	0.655	0.001
68	E									
69	E									
70	D									
71	E									
72	E	8.53	122.44	56.57	56.96	176.66	177.07	0.051	0.909	0.009
73	G	8.40	110.00	56.97	45.23	177.07	173.82	0.189	0.980	0.009
74	Y	8.10	120.39	45.23	58.01	173.82	175.59	0.372	0.937	0.009
75	N	8.38	121.70	58.04	52.86	175.59	174.51	0.999	0.606	0.009
76	D	8.25	121.73	52.87	54.53	174.53	176.64	1.000	0.604	0.009
77	G	8.31	108.99	54.54	45.34	176.60	174.09	0.447	0.801	0.009
78	E	8.23	120.80	45.31	56.33	174.07	176.48	0.132	0.742	0.009

79	V	8.25	121.13	56.31	61.78	176.49	175.72	0.528	0.262	0.009
80	D	8.46	124.90	61.84	54.15	175.73	175.85	0.263	0.590	0.002
81	D	8.35	121.76	54.16	54.29	175.84	176.21	0.052	0.841	0.002
82	E	8.41	121.38	54.30	56.47	176.22	176.47	0.040	0.403	0.009
83	E	8.45	122.36	56.43	56.31	176.46	176.13	0.004	0.232	0.002
84	D	8.50	122.50	56.28	54.32	176.12	175.77	0.063	0.209	0.009
85	E									
86	E									
87	E									
88	F									
89	G									
90	E	8.44	120.68	45.22	57.49	174.37	177.44	0.138	0.327	0.009
91	E	8.66	120.80	57.51	57.51	177.44	177.16	0.014	0.177	0.002
92	E	8.37	121.07	57.53	57.18	177.16	177.08	0.005	0.163	0.002
93	R	8.29	121.34	57.17	56.96	177.09	177.30	0.024	0.229	0.009
94	G	8.44	109.18	56.97	45.60	177.31	174.44	0.105	0.498	0.009
95	Q	8.17	119.66	45.60	55.92	174.45	176.21	0.394	0.885	0.009
96	K	8.32	122.25	55.93	56.50	176.19	176.59	0.157	0.936	0.009
97	R	8.36	122.56	56.47	56.03	176.60	176.16	0.109	0.994	0.009
98	K	8.43	123.70	56.08	56.38	176.17	176.30	0.041	0.999	0.002
99	R	8.45	123.33	56.37	55.93	176.27	176.08	0.039	1.000	0.002
100	E	8.63	124.06	55.93	54.41	176.10	174.99	0.784	0.915	0.009
101	P									
102	E	8.62	119.04	63.95	56.84	177.34	176.27	1.000	0.116	0.009
103	D	8.19	120.97	56.83	54.41	176.28	176.23	0.022	0.853	0.002
104	E	8.39	121.62	54.41	56.69	176.22	176.93	0.073	0.910	0.009
105	G	8.48	109.93	56.67	45.24	176.94	173.96	0.111	0.575	0.009
106	E	8.33	120.30	45.23	56.14	173.98	176.27	0.086	0.340	0.009
107	D	8.49	121.60	56.13	54.37	176.25	175.92	0.071	0.380	0.009
108	D	8.35	121.08	54.30	54.29	175.90	175.93	0.007	0.519	0.001
109	D	8.35	121.28	54.30	54.31	175.90	175.24	0.002	0.007	0.002
110	D	7.98	126.01	54.31	56.02	175.25	176.11	0.990	0.053	0.009

Assigned amide groups that match the reference assignment are indicated by green shading. Missing amide groups that are present in the reference assignments are indicated by white shading. Amide groups that were assigned to residues that are not assigned in the reference assignments are indicated by orange shading. Indicated with yellow shading are those assignments made by FLYA that match those made by BARASA and correspond to previously unreported assignments. Residues that were not assigned by either BARASA or in the reference assignments were considered matching. Non-amide resonances were not considered when determining assignment accuracy.
The table is included in Excel format in the Source Data file.

**Supplementary Table 11 – BARASA runs using SPARTA+ predicted chemical shifts‡**			
	**Fraction**		
**Protein**	**Matching**	**Missing**	**Mismatching**
IL-1Ra	0.974	0.020	0.007
IL-1β	1.000	0.000	0.000
IGPS	0.931	0.056	0.012
MBP	0.978	0.005	0.016
CY1	0.909	0.053	0.038
ecTS	0.973	0.015	0.013

‡ Same as in Fig. 2 of the main text except that SPARTA+ was used to predict the chemical shifts
The table is included in Excel format in the Source Data file.

**Supplementary Table 12 – Execution time for BARASA on the test protein set‡**			
**Protein**	**Avg Run Time (min)**	**Std Dev (min)**	**Total time (min)**
IL-1Ra	3.6	1.6	6
IL-1β	0.62	0.14	1
IGPS	1.86	0.5	4
MBP	3.9	1.3	7
CY1	28	11	52
ecTS	5.6	1.0	11
V5dm	0.85	0.17	2
hIDD	1.08	0.35	2

‡Performed on a 2019 6 core MacBook Pro (Intel processor) running 20 annealing runs with up to 12 simultaneous annealing runs at any given time.
The table is included in Excel format in the Source Data file.

Supplementary Table 13 – BARASA results from randomly depleted MBP crosspeaks									
Data Set Properties							BARASA results		
Fraction Retained			Possible Connectivities (± std)*				Fraction (± std)*		
Cα	Cβ	CO	CA	CB	CO	Assigned Spin Systems	Matching	Missing	Mismatching
1	1	1	307 ± 0	277 ± 0	299 ± 0	335 ± 0	0.982 ± 0.001	0.005 ± 0	0.012 ± 0.001
1	1	0.88	307 ± 0	277 ± 0	252 ± 4	335 ± 0	0.983 ± 0.003	0.005 ± 0.003	0.012 ± 0.001
1	1	0.75	307 ± 0	277 ± 0	203 ± 6	335 ± 0	0.982 ± 0.004	0.007 ± 0.004	0.011 ± 0.002
1	1	0.25	307 ± 0	277 ± 0	28 ± 3	335 ± 0	0.982 ± 0.005	0.006 ± 0.004	0.011 ± 0.003
1	1	0	307 ± 0	277 ± 0	0 ± 0	335 ± 0	0.978 ± 0.007	0.008 ± 0.005	0.014 ± 0.003
1	0.88	1	307 ± 0	240 ± 4	299 ± 0	335 ± 0	0.984 ± 0.003	0.004 ± 0.002	0.012 ± 0.002
1	0.88	0.88	307 ± 0	241 ± 5	255 ± 5	335 ± 0	0.984 ± 0.004	0.005 ± 0.004	0.011 ± 0.003
1	0.88	0.75	307 ± 0	238 ± 5	207 ± 6	335 ± 0	0.979 ± 0.006	0.008 ± 0.004	0.012 ± 0.003
1	0.88	0.25	307 ± 0	238 ± 4	30 ± 8	335 ± 0	0.979 ± 0.008	0.01 ± 0.006	0.011 ± 0.003
1	0.88	0	307 ± 0	244 ± 4	0 ± 0	335 ± 0	0.978 ± 0.009	0.01 ± 0.008	0.012 ± 0.003
1	0.75	1	307 ± 0	199 ± 6	299 ± 0	335 ± 0	0.986 ± 0.003	0.003 ± 0.002	0.011 ± 0.002
1	0.75	0.88	307 ± 0	193 ± 6	256 ± 6	335 ± 0	0.987 ± 0.005	0.004 ± 0.004	0.009 ± 0.001
1	0.75	0.75	307 ± 0	195 ± 8	206 ± 6	335 ± 0	0.981 ± 0.005	0.006 ± 0.004	0.013 ± 0.004
1	0.75	0.25	307 ± 0	191 ± 8	33 ± 5	335 ± 0	0.977 ± 0.008	0.012 ± 0.009	0.011 ± 0.003
1	0.75	0	307 ± 0	193 ± 11	0 ± 0	335 ± 0	0.983 ± 0.006	0.005 ± 0.004	0.012 ± 0.003
1	0.25	1	307 ± 0	31 ± 4	299 ± 0	335 ± 0	0.989 ± 0.004	0.003 ± 0.003	0.008 ± 0.003
1	0.25	0.88	307 ± 0	28 ± 3	255 ± 8	335 ± 0	0.985 ± 0.004	0.007 ± 0.005	0.008 ± 0.003
1	0.25	0.75	307 ± 0	31 ± 3	203 ± 8	335 ± 0	0.977 ± 0.008	0.014 ± 0.008	0.009 ± 0.003
1	0.25	0.25	307 ± 0	31 ± 7	32 ± 3	335 ± 0	0.973 ± 0.006	0.018 ± 0.006	0.009 ± 0.003
1	0.25	0	307 ± 0	33 ± 6	0 ± 0	335 ± 0	0.985 ± 0.003	0.008 ± 0.003	0.008 ± 0.003
1	0	1	307 ± 0	0 ± 0	299 ± 0	335 ± 0	0.994 ± 0.002	0.001 ± 0.002	0.005 ± 0
1	0	0.88	307 ± 0	0 ± 0	255 ± 7	335 ± 0	0.988 ± 0.003	0.003 ± 0.002	0.009 ± 0.002
1	0	0.75	307 ± 0	0 ± 0	204 ± 7	335 ± 0	0.985 ± 0.006	0.005 ± 0.004	0.01 ± 0.005
1	0	0.25	307 ± 0	0 ± 0	30 ± 4	335 ± 0	0.978 ± 0.007	0.013 ± 0.006	0.008 ± 0.004
1	0	0	307 ± 0	0 ± 0	0 ± 0	335 ± 0	0.98 ± 0.001	0.009 ± 0.001	0.011 ± 0
0.88	1	1	270 ± 8	277 ± 0	299 ± 0	335 ± 0	0.981 ± 0.006	0.006 ± 0.005	0.012 ± 0.002
0.88	1	0.88	269 ± 6	277 ± 0	259 ± 8	335 ± 0	0.981 ± 0.004	0.006 ± 0.003	0.013 ± 0.002
0.88	1	0.75	268 ± 4	277 ± 0	204 ± 7	335 ± 0	0.98 ± 0.005	0.008 ± 0.005	0.012 ± 0.003
0.88	1	0.25	268 ± 6	277 ± 0	31 ± 5	335 ± 0	0.969 ± 0.009	0.015 ± 0.007	0.016 ± 0.004
0.88	1	0	270 ± 4	277 ± 0	0 ± 0	335 ± 0	0.983 ± 0.007	0.005 ± 0.005	0.012 ± 0.004
0.88	0.88	1	269 ± 5	240 ± 7	299 ± 0	335 ± 0	0.987 ± 0.003	0.004 ± 0.003	0.009 ± 0.002

0.88	0.88	0.88	269 ± 4	240 ± 4	253 ± 6	335 ± 0	0.976 ± 0.007	0.009 ± 0.005	0.015 ± 0.005
0.88	0.88	0.75	266 ± 5	238 ± 3	207 ± 7	335 ± 0	0.978 ± 0.006	0.01 ± 0.005	0.012 ± 0.003
0.88	0.88	0.25	270 ± 5	238 ± 6	30 ± 5	335 ± 1	0.965 ± 0.009	0.02 ± 0.008	0.015 ± 0.004
0.88	0.88	0	267 ± 5	238 ± 6	0 ± 0	335 ± 0	0.972 ± 0.011	0.012 ± 0.009	0.016 ± 0.006
0.88	0.75	1	270 ± 6	190 ± 8	299 ± 0	335 ± 0	0.983 ± 0.004	0.005 ± 0.002	0.012 ± 0.003
0.88	0.75	0.88	270 ± 3	195 ± 5	256 ± 7	335 ± 0	0.977 ± 0.008	0.008 ± 0.006	0.015 ± 0.005
0.88	0.75	0.75	267 ± 5	195 ± 7	205 ± 5	335 ± 0	0.977 ± 0.009	0.011 ± 0.007	0.012 ± 0.003
0.88	0.75	0.25	269 ± 7	194 ± 4	31 ± 4	335 ± 0	0.961 ± 0.01	0.024 ± 0.008	0.015 ± 0.006
0.88	0.75	0	267 ± 5	198 ± 10	0 ± 0	335 ± 0	0.967 ± 0.011	0.018 ± 0.007	0.015 ± 0.006
0.88	0.25	1	266 ± 6	32 ± 5	299 ± 0	335 ± 0	0.98 ± 0.006	0.007 ± 0.005	0.013 ± 0.004
0.88	0.25	0.88	264 ± 6	28 ± 8	255 ± 5	335 ± 0	0.971 ± 0.009	0.018 ± 0.007	0.011 ± 0.005
0.88	0.25	0.75	264 ± 3	33 ± 7	203 ± 10	335 ± 0	0.964 ± 0.013	0.022 ± 0.012	0.014 ± 0.006
0.88	0.25	0.25	266 ± 6	32 ± 4	31 ± 5	335 ± 1	0.913 ± 0.015	0.06 ± 0.016	0.027 ± 0.007
0.88	0.25	0	266 ± 4	33 ± 5	0 ± 0	335 ± 1	0.916 ± 0.022	0.053 ± 0.017	0.031 ± 0.011
0.88	0	1	262 ± 6	0 ± 0	299 ± 0	335 ± 0	0.983 ± 0.006	0.006 ± 0.005	0.011 ± 0.004
0.88	0	0.88	266 ± 7	0 ± 0	255 ± 3	335 ± 0	0.967 ± 0.009	0.021 ± 0.007	0.012 ± 0.004
0.88	0	0.75	266 ± 7	0 ± 0	206 ± 7	335 ± 0	0.956 ± 0.01	0.028 ± 0.009	0.016 ± 0.006
0.88	0	0.25	264 ± 4	0 ± 0	30 ± 3	335 ± 0	0.884 ± 0.026	0.086 ± 0.017	0.03 ± 0.011
0.88	0	0	269 ± 8	0 ± 0	0 ± 0	335 ± 1	0.863 ± 0.033	0.082 ± 0.024	0.055 ± 0.018
0.75	1	1	223 ± 7	277 ± 0	299 ± 0	335 ± 0	0.982 ± 0.004	0.006 ± 0.002	0.011 ± 0.003
0.75	1	0.88	223 ± 6	277 ± 0	256 ± 7	335 ± 0	0.983 ± 0.003	0.006 ± 0.003	0.011 ± 0.003
0.75	1	0.75	227 ± 8	277 ± 0	207 ± 5	335 ± 1	0.978 ± 0.006	0.01 ± 0.007	0.012 ± 0.005
0.75	1	0.25	224 ± 7	277 ± 0	27 ± 4	335 ± 0	0.975 ± 0.008	0.013 ± 0.008	0.012 ± 0.004
0.75	1	0	221 ± 6	277 ± 0	0 ± 0	335 ± 0	0.973 ± 0.008	0.011 ± 0.006	0.016 ± 0.004
0.75	0.88	1	219 ± 8	239 ± 5	299 ± 0	335 ± 0	0.98 ± 0.005	0.007 ± 0.002	0.013 ± 0.003
0.75	0.88	0.88	221 ± 9	239 ± 7	255 ± 6	335 ± 0	0.976 ± 0.01	0.011 ± 0.01	0.012 ± 0.002
0.75	0.88	0.75	223 ± 7	240 ± 7	203 ± 7	335 ± 0	0.97 ± 0.009	0.016 ± 0.007	0.014 ± 0.005
0.75	0.88	0.25	220 ± 7	240 ± 6	31 ± 7	335 ± 0	0.966 ± 0.009	0.023 ± 0.008	0.011 ± 0.004
0.75	0.88	0	219 ± 6	242 ± 3	0 ± 0	335 ± 0	0.964 ± 0.007	0.018 ± 0.007	0.018 ± 0.006
0.75	0.75	1	220 ± 8	192 ± 7	299 ± 0	335 ± 0	0.983 ± 0.005	0.006 ± 0.004	0.011 ± 0.003
0.75	0.75	0.88	220 ± 7	193 ± 6	257 ± 7	335 ± 0	0.973 ± 0.007	0.013 ± 0.005	0.014 ± 0.004
0.75	0.75	0.75	221 ± 5	193 ± 7	208 ± 6	335 ± 0	0.965 ± 0.012	0.02 ± 0.008	0.015 ± 0.008
0.75	0.75	0.25	219 ± 6	194 ± 6	32 ± 4	335 ± 0	0.944 ± 0.012	0.038 ± 0.01	0.018 ± 0.006
0.75	0.75	0	220 ± 7	194 ± 7	0 ± 0	334 ± 1	0.942 ± 0.011	0.039 ± 0.012	0.019 ± 0.006
0.75	0.25	1	216 ± 10	29 ± 4	299 ± 0	335 ± 0	0.973 ± 0.007	0.012 ± 0.006	0.016 ± 0.006
0.75	0.25	0.88	217 ± 5	29 ± 5	254 ± 8	335 ± 0	0.95 ± 0.012	0.035 ± 0.01	0.016 ± 0.004
0.75	0.25	0.75	214 ± 11	31 ± 6	209 ± 6	335 ± 1	0.922 ± 0.021	0.055 ± 0.02	0.023 ± 0.007

0.75	0.25	0.25	215 ± 10	28 ± 5	33 ± 7	334 ± 1	0.799 ± 0.042	0.151 ± 0.037	0.05 ± 0.013
0.75	0.25	0	220 ± 5	30 ± 4	0 ± 0	333 ± 2	0.798 ± 0.028	0.146 ± 0.029	0.056 ± 0.011
0.75	0	1	215 ± 6	0 ± 0	299 ± 0	335 ± 0	0.964 ± 0.01	0.021 ± 0.008	0.015 ± 0.006
0.75	0	0.88	210 ± 8	0 ± 0	255 ± 5	335 ± 0	0.939 ± 0.018	0.042 ± 0.016	0.02 ± 0.006
0.75	0	0.75	216 ± 10	0 ± 0	206 ± 6	335 ± 0	0.899 ± 0.026	0.078 ± 0.025	0.023 ± 0.005
0.75	0	0.25	220 ± 4	0 ± 0	34 ± 5	332 ± 2	0.684 ± 0.042	0.246 ± 0.04	0.07 ± 0.014
0.75	0	0	215 ± 8	0 ± 0	0 ± 0	329 ± 2	0.631 ± 0.038	0.253 ± 0.031	0.116 ± 0.024
0.25	1	1	57 ± 5	277 ± 0	299 ± 0	334 ± 0	0.985 ± 0.003	0.004 ± 0.003	0.01 ± 0.001
0.25	1	0.88	60 ± 5	277 ± 0	255 ± 6	334 ± 1	0.982 ± 0.002	0.006 ± 0.003	0.012 ± 0.003
0.25	1	0.75	60 ± 5	277 ± 0	204 ± 8	335 ± 1	0.979 ± 0.006	0.01 ± 0.005	0.012 ± 0.002
0.25	1	0.25	55 ± 4	277 ± 0	32 ± 3	334 ± 0	0.958 ± 0.01	0.029 ± 0.005	0.013 ± 0.007
0.25	1	0	58 ± 4	277 ± 0	0 ± 0	334 ± 1	0.967 ± 0.006	0.021 ± 0.006	0.011 ± 0.004
0.25	0.88	1	52 ± 7	243 ± 7	299 ± 0	334 ± 0	0.977 ± 0.008	0.012 ± 0.007	0.011 ± 0.002
0.25	0.88	0.88	58 ± 8	241 ± 6	257 ± 5	334 ± 0	0.971 ± 0.006	0.015 ± 0.006	0.014 ± 0.003
0.25	0.88	0.75	55 ± 5	238 ± 5	205 ± 10	334 ± 0	0.959 ± 0.008	0.026 ± 0.01	0.015 ± 0.003
0.25	0.88	0.25	57 ± 8	241 ± 4	29 ± 4	334 ± 1	0.922 ± 0.013	0.055 ± 0.012	0.023 ± 0.01
0.25	0.88	0	53 ± 6	238 ± 5	0 ± 0	333 ± 1	0.902 ± 0.013	0.066 ± 0.012	0.032 ± 0.012
0.25	0.75	1	53 ± 8	195 ± 4	299 ± 0	334 ± 0	0.973 ± 0.009	0.016 ± 0.007	0.011 ± 0.004
0.25	0.75	0.88	49 ± 5	192 ± 5	256 ± 7	334 ± 1	0.955 ± 0.008	0.031 ± 0.008	0.014 ± 0.007
0.25	0.75	0.75	53 ± 4	193 ± 9	203 ± 8	334 ± 0	0.934 ± 0.026	0.046 ± 0.023	0.021 ± 0.006
0.25	0.75	0.25	55 ± 6	194 ± 6	34 ± 8	333 ± 1	0.819 ± 0.023	0.146 ± 0.018	0.035 ± 0.01
0.25	0.75	0	50 ± 5	194 ± 10	0 ± 0	331 ± 2	0.799 ± 0.042	0.142 ± 0.025	0.059 ± 0.021
0.25	0.25	1	38 ± 5	32 ± 4	299 ± 0	335 ± 0	0.876 ± 0.028	0.097 ± 0.026	0.027 ± 0.012
0.25	0.25	0.88	39 ± 4	31 ± 5	255 ± 5	334 ± 1	0.744 ± 0.041	0.219 ± 0.035	0.037 ± 0.014
0.25	0.25	0.75	39 ± 6	32 ± 5	205 ± 7	331 ± 2	0.608 ± 0.056	0.334 ± 0.057	0.058 ± 0.007
0.25	0.25	0.25	41 ± 6	29 ± 2	30 ± 4	307 ± 5	0.316 ± 0.025	0.573 ± 0.029	0.111 ± 0.016
0.25	0.25	0	37 ± 7	28 ± 6	0 ± 0	270 ± 11	0.263 ± 0.029	0.601 ± 0.03	0.137 ± 0.016
0.25	0	1	37 ± 5	0 ± 0	299 ± 0	334 ± 0	0.675 ± 0.031	0.295 ± 0.03	0.03 ± 0.008
0.25	0	0.88	35 ± 5	0 ± 0	254 ± 4	334 ± 1	0.523 ± 0.031	0.428 ± 0.028	0.049 ± 0.022
0.25	0	0.75	32 ± 3	0 ± 0	209 ± 6	331 ± 2	0.419 ± 0.032	0.525 ± 0.034	0.056 ± 0.01
0.25	0	0.25	34 ± 5	0 ± 0	29 ± 6	272 ± 6	0.169 ± 0.014	0.743 ± 0.027	0.087 ± 0.022
0.25	0	0	34 ± 6	0 ± 0	0 ± 0	192 ± 10	0.159 ± 0.012	0.769 ± 0.019	0.071 ± 0.011
0	1	1	27 ± 0	277 ± 0	299 ± 0	334 ± 0	0.986 ± 0.002	0.004 ± 0.001	0.01 ± 0.002
0	1	0.88	27 ± 0	277 ± 0	257 ± 5	334 ± 0	0.981 ± 0.006	0.008 ± 0.004	0.011 ± 0.002
0	1	0.75	27 ± 0	277 ± 0	202 ± 12	334 ± 0	0.973 ± 0.01	0.013 ± 0.009	0.013 ± 0.003
0	1	0.25	27 ± 0	277 ± 0	33 ± 4	334 ± 1	0.952 ± 0.005	0.035 ± 0.005	0.013 ± 0.005
0	1	0	27 ± 0	277 ± 0	0 ± 0	333 ± 0	0.95 ± 0.004	0.037 ± 0.001	0.013 ± 0.004

0	0.88	1	24 ± 2	240 ± 3	299 ± 0	334 ± 0	0.98 ± 0.006	0.008 ± 0.003	0.012 ± 0.004
0	0.88	0.88	22 ± 2	241 ± 4	255 ± 6	334 ± 0	0.972 ± 0.008	0.014 ± 0.006	0.015 ± 0.005
0	0.88	0.75	23 ± 2	242 ± 5	204 ± 4	334 ± 0	0.966 ± 0.012	0.02 ± 0.007	0.014 ± 0.006
0	0.88	0.25	23 ± 2	238 ± 5	30 ± 7	333 ± 1	0.894 ± 0.027	0.08 ± 0.014	0.027 ± 0.016
0	0.88	0	24 ± 1	238 ± 4	0 ± 0	332 ± 1	0.881 ± 0.024	0.085 ± 0.02	0.035 ± 0.011
0	0.75	1	18 ± 3	188 ± 7	299 ± 0	334 ± 0	0.969 ± 0.008	0.016 ± 0.005	0.014 ± 0.005
0	0.75	0.88	19 ± 2	193 ± 6	255 ± 9	334 ± 0	0.954 ± 0.011	0.032 ± 0.01	0.014 ± 0.006
0	0.75	0.75	18 ± 2	195 ± 8	201 ± 8	334 ± 0	0.926 ± 0.022	0.057 ± 0.023	0.017 ± 0.007
0	0.75	0.25	18 ± 3	194 ± 3	30 ± 6	331 ± 1	0.744 ± 0.025	0.193 ± 0.015	0.063 ± 0.014
0	0.75	0	20 ± 3	197 ± 9	0 ± 0	328 ± 2	0.739 ± 0.035	0.19 ± 0.034	0.07 ± 0.012
0	0.25	1	2 ± 1	31 ± 6	299 ± 0	334 ± 0	0.748 ± 0.024	0.219 ± 0.023	0.033 ± 0.01
0	0.25	0.88	4 ± 2	33 ± 6	256 ± 5	333 ± 1	0.594 ± 0.04	0.363 ± 0.037	0.043 ± 0.015
0	0.25	0.75	3 ± 1	26 ± 3	209 ± 6	330 ± 2	0.477 ± 0.039	0.461 ± 0.035	0.062 ± 0.014
0	0.25	0.25	3 ± 1	29 ± 6	31 ± 5	269 ± 9	0.212 ± 0.027	0.689 ± 0.029	0.099 ± 0.031
0	0.25	0	3 ± 1	28 ± 5	0 ± 0	191 ± 6	0.176 ± 0.019	0.745 ± 0.019	0.079 ± 0.008
0	0	1	0 ± 0	0 ± 0	299 ± 0	334 ± 0	0.396 ± 0.017	0.578 ± 0.016	0.026 ± 0.005
0	0	0.88	0 ± 0	0 ± 0	259 ± 4	332 ± 2	0.298 ± 0.017	0.665 ± 0.02	0.037 ± 0.011
0	0	0.75	0 ± 0	0 ± 0	203 ± 11	324 ± 2	0.23 ± 0.018	0.735 ± 0.022	0.035 ± 0.01
0	0	0.25	0 ± 0	0 ± 0	32 ± 5	186 ± 5	0.108 ± 0.006	0.867 ± 0.012	0.026 ± 0.009

*Values are means and standard deviations from 10 independently generated data sets. Possible connectivities refer to the number of matching (i), (i-1) pairs of resonances of adjacent spin systems that are still present in the depleted data set. Entries of zero for error indicate that the standard deviation is less than the precision indicated for the observed value.
The table is included in Excel format in the Source Data file.

Supplementary Table 14 – BARASA results from randomly depleted ecTS crosspeaks									
**Data Set Properties**							**BARASA results**		
**Fraction Retained**			**Possible Connectivities (± std)***				**Fraction (± std)***		
**Cα**	**Cβ**	**CO**	**CA**	**CB**	**CO**	**Assigned Spin Systems**	**Matching**	**Missing**	**Mismatching**
1	1	1	207 ± 0	102 ± 0	199 ± 0	236 ± 0	0.971 ± 0.004	0.022 ± 0.004	0.007 ± 0.002
1	1	0.88	207 ± 0	102 ± 0	169 ± 7	236 ± 0	0.97 ± 0.008	0.023 ± 0.007	0.007 ± 0.003
1	1	0.75	207 ± 0	102 ± 0	132 ± 9	236 ± 0	0.97 ± 0.011	0.022 ± 0.011	0.007 ± 0.002
1	1	0.25	207 ± 0	102 ± 0	21 ± 4	236 ± 0	0.959 ± 0.007	0.031 ± 0.008	0.009 ± 0.004
1	1	0	207 ± 0	102 ± 0	0 ± 0	236 ± 0	0.97 ± 0.005	0.025 ± 0.005	0.006 ± 0.002
1	0.88	1	207 ± 0	88 ± 5	199 ± 0	236 ± 0	0.969 ± 0.006	0.023 ± 0.006	0.007 ± 0.002
1	0.88	0.88	207 ± 0	87 ± 3	170 ± 3	236 ± 0	0.972 ± 0.009	0.021 ± 0.008	0.007 ± 0.006
1	0.88	0.75	207 ± 0	89 ± 2	136 ± 7	236 ± 0	0.963 ± 0.006	0.027 ± 0.006	0.011 ± 0.005
1	0.88	0.25	207 ± 0	85 ± 3	22 ± 3	236 ± 0	0.953 ± 0.008	0.034 ± 0.009	0.013 ± 0.005
1	0.88	0	207 ± 0	86 ± 3	0 ± 0	236 ± 0	0.963 ± 0.007	0.025 ± 0.008	0.012 ± 0.004
1	0.75	1	207 ± 0	71 ± 4	199 ± 0	236 ± 0	0.97 ± 0.005	0.021 ± 0.005	0.009 ± 0.003
1	0.75	0.88	207 ± 0	68 ± 5	168 ± 5	236 ± 0	0.966 ± 0.011	0.023 ± 0.01	0.011 ± 0.007
1	0.75	0.75	207 ± 0	71 ± 3	139 ± 7	236 ± 0	0.959 ± 0.007	0.03 ± 0.006	0.011 ± 0.006
1	0.75	0.25	207 ± 0	70 ± 3	20 ± 6	236 ± 0	0.947 ± 0.012	0.039 ± 0.011	0.014 ± 0.005
1	0.75	0	207 ± 0	70 ± 3	0 ± 0	236 ± 0	0.949 ± 0.013	0.033 ± 0.011	0.017 ± 0.009
1	0.25	1	207 ± 0	11 ± 3	199 ± 0	236 ± 0	0.961 ± 0.015	0.028 ± 0.013	0.011 ± 0.005
1	0.25	0.88	207 ± 0	10 ± 2	171 ± 4	236 ± 0	0.955 ± 0.011	0.036 ± 0.01	0.009 ± 0.006
1	0.25	0.75	207 ± 0	10 ± 4	137 ± 5	236 ± 0	0.938 ± 0.013	0.047 ± 0.016	0.015 ± 0.007
1	0.25	0.25	207 ± 0	10 ± 2	21 ± 5	236 ± 0	0.902 ± 0.021	0.074 ± 0.015	0.024 ± 0.007
1	0.25	0	207 ± 0	10 ± 3	0 ± 0	236 ± 0	0.893 ± 0.018	0.073 ± 0.015	0.034 ± 0.006
1	0	1	207 ± 0	0 ± 0	199 ± 0	236 ± 0	0.973 ± 0.004	0.016 ± 0.002	0.011 ± 0.006
1	0	0.88	207 ± 0	0 ± 0	171 ± 4	236 ± 0	0.953 ± 0.011	0.03 ± 0.008	0.017 ± 0.007
1	0	0.75	207 ± 0	0 ± 0	135 ± 6	236 ± 0	0.939 ± 0.016	0.039 ± 0.009	0.022 ± 0.011
1	0	0.25	207 ± 0	0 ± 0	22 ± 3	236 ± 0	0.868 ± 0.017	0.085 ± 0.007	0.047 ± 0.013
1	0	0	180 ± 3	0 ± 0	0 ± 0	236 ± 0	0.859 ± 0.007	0.083 ± 0.008	0.058 ± 0.006
0.88	1	1	179 ± 4	102 ± 0	199 ± 0	236 ± 0	0.968 ± 0.009	0.024 ± 0.009	0.008 ± 0.003
0.88	1	0.88	178 ± 4	102 ± 0	172 ± 4	236 ± 0	0.968 ± 0.008	0.024 ± 0.009	0.008 ± 0.005
0.88	1	0.75	182 ± 4	102 ± 0	133 ± 7	236 ± 0	0.968 ± 0.009	0.025 ± 0.007	0.007 ± 0.005
0.88	1	0.25	182 ± 4	102 ± 0	21 ± 5	236 ± 0	0.956 ± 0.008	0.034 ± 0.008	0.009 ± 0.004
0.88	1	0	180 ± 6	102 ± 0	0 ± 0	236 ± 0	0.96 ± 0.006	0.031 ± 0.004	0.009 ± 0.004
0.88	0.88	1	182 ± 6	87 ± 2	199 ± 0	236 ± 0	0.968 ± 0.008	0.023 ± 0.007	0.008 ± 0.003

0.88	0.88	0.88	182 ± 5	88 ± 3	171 ± 5	236 ± 0	0.959 ± 0.009	0.032 ± 0.008	0.009 ± 0.007
0.88	0.88	0.75	180 ± 2	89 ± 2	136 ± 5	236 ± 0	0.957 ± 0.014	0.032 ± 0.013	0.011 ± 0.006
0.88	0.88	0.25	179 ± 5	86 ± 4	21 ± 3	236 ± 0	0.94 ± 0.013	0.044 ± 0.012	0.017 ± 0.006
0.88	0.88	0	178 ± 3	88 ± 2	0 ± 0	236 ± 0	0.94 ± 0.014	0.041 ± 0.012	0.019 ± 0.005
0.88	0.75	1	179 ± 4	69 ± 3	199 ± 0	236 ± 0	0.962 ± 0.007	0.027 ± 0.008	0.011 ± 0.004
0.88	0.75	0.88	179 ± 4	70 ± 4	170 ± 6	236 ± 0	0.956 ± 0.013	0.033 ± 0.012	0.011 ± 0.006
0.88	0.75	0.75	179 ± 5	68 ± 3	138 ± 8	236 ± 0	0.949 ± 0.012	0.041 ± 0.011	0.01 ± 0.003
0.88	0.75	0.25	182 ± 3	67 ± 5	24 ± 5	236 ± 0	0.925 ± 0.012	0.053 ± 0.012	0.023 ± 0.007
0.88	0.75	0	180 ± 3	73 ± 4	0 ± 0	236 ± 0	0.934 ± 0.008	0.042 ± 0.009	0.025 ± 0.008
0.88	0.25	1	182 ± 3	12 ± 3	199 ± 0	236 ± 0	0.955 ± 0.014	0.031 ± 0.011	0.014 ± 0.006
0.88	0.25	0.88	181 ± 4	11 ± 4	169 ± 4	236 ± 0	0.942 ± 0.015	0.041 ± 0.014	0.017 ± 0.007
0.88	0.25	0.75	180 ± 5	9 ± 3	135 ± 4	236 ± 0	0.916 ± 0.014	0.067 ± 0.015	0.017 ± 0.008
0.88	0.25	0.25	180 ± 6	12 ± 3	22 ± 6	236 ± 1	0.844 ± 0.029	0.116 ± 0.027	0.04 ± 0.011
0.88	0.25	0	182 ± 4	11 ± 3	0 ± 0	236 ± 1	0.812 ± 0.033	0.126 ± 0.028	0.062 ± 0.021
0.88	0	1	180 ± 6	0 ± 0	199 ± 0	236 ± 0	0.946 ± 0.014	0.037 ± 0.014	0.017 ± 0.006
0.88	0	0.88	179 ± 3	0 ± 0	170 ± 6	236 ± 0	0.934 ± 0.014	0.047 ± 0.015	0.019 ± 0.006
0.88	0	0.75	181 ± 5	0 ± 0	141 ± 6	236 ± 0	0.891 ± 0.017	0.081 ± 0.024	0.029 ± 0.01
0.88	0	0.25	181 ± 6	0 ± 0	22 ± 5	236 ± 1	0.75 ± 0.039	0.188 ± 0.034	0.063 ± 0.018
0.88	0	0	144 ± 3	0 ± 0	0 ± 0	235 ± 1	0.734 ± 0.058	0.184 ± 0.047	0.082 ± 0.021
0.75	1	1	147 ± 4	102 ± 0	199 ± 0	236 ± 0	0.961 ± 0.009	0.031 ± 0.009	0.008 ± 0.007
0.75	1	0.88	148 ± 5	102 ± 0	170 ± 4	236 ± 0	0.963 ± 0.01	0.028 ± 0.007	0.009 ± 0.006
0.75	1	0.75	147 ± 5	102 ± 0	134 ± 7	236 ± 0	0.958 ± 0.016	0.032 ± 0.015	0.01 ± 0.005
0.75	1	0.25	148 ± 7	102 ± 0	21 ± 4	236 ± 0	0.949 ± 0.012	0.04 ± 0.008	0.011 ± 0.007
0.75	1	0	147 ± 6	102 ± 0	0 ± 0	235 ± 1	0.944 ± 0.005	0.038 ± 0.006	0.018 ± 0.006
0.75	0.88	1	149 ± 5	85 ± 4	199 ± 0	236 ± 0	0.968 ± 0.007	0.026 ± 0.005	0.006 ± 0.005
0.75	0.88	0.88	145 ± 9	86 ± 2	168 ± 3	236 ± 0	0.96 ± 0.007	0.033 ± 0.006	0.007 ± 0.004
0.75	0.88	0.75	148 ± 5	89 ± 2	137 ± 9	236 ± 0	0.946 ± 0.017	0.039 ± 0.013	0.014 ± 0.012
0.75	0.88	0.25	148 ± 8	87 ± 4	21 ± 4	235 ± 1	0.933 ± 0.01	0.05 ± 0.01	0.017 ± 0.007
0.75	0.88	0	149 ± 7	88 ± 3	0 ± 0	235 ± 1	0.933 ± 0.011	0.044 ± 0.007	0.022 ± 0.011
0.75	0.75	1	143 ± 8	67 ± 6	199 ± 0	236 ± 0	0.951 ± 0.011	0.038 ± 0.013	0.011 ± 0.004
0.75	0.75	0.88	144 ± 5	72 ± 5	171 ± 5	236 ± 0	0.947 ± 0.013	0.04 ± 0.012	0.013 ± 0.005
0.75	0.75	0.75	141 ± 5	69 ± 4	138 ± 6	236 ± 0	0.941 ± 0.016	0.045 ± 0.014	0.015 ± 0.008
0.75	0.75	0.25	144 ± 8	70 ± 4	21 ± 3	236 ± 0	0.904 ± 0.011	0.07 ± 0.01	0.026 ± 0.007
0.75	0.75	0	148 ± 5	72 ± 3	0 ± 0	235 ± 1	0.909 ± 0.016	0.066 ± 0.02	0.025 ± 0.013
0.75	0.25	1	149 ± 5	10 ± 3	199 ± 0	236 ± 1	0.934 ± 0.008	0.05 ± 0.013	0.016 ± 0.009
0.75	0.25	0.88	149 ± 8	10 ± 2	170 ± 6	236 ± 1	0.897 ± 0.022	0.082 ± 0.016	0.021 ± 0.01
0.75	0.25	0.75	150 ± 4	9 ± 4	135 ± 5	236 ± 1	0.895 ± 0.017	0.081 ± 0.018	0.024 ± 0.006

0.75	0.25	0.25	145 ± 6	11 ± 3	21 ± 5	234 ± 1	0.737 ± 0.037	0.203 ± 0.033	0.06 ± 0.016
0.75	0.25	0	146 ± 5	11 ± 3	0 ± 0	233 ± 2	0.703 ± 0.027	0.222 ± 0.02	0.075 ± 0.019
0.75	0	1	147 ± 5	0 ± 0	199 ± 0	235 ± 1	0.918 ± 0.014	0.059 ± 0.012	0.023 ± 0.006
0.75	0	0.88	149 ± 4	0 ± 0	171 ± 5	236 ± 1	0.889 ± 0.021	0.082 ± 0.019	0.03 ± 0.013
0.75	0	0.75	146 ± 6	0 ± 0	136 ± 6	236 ± 0	0.854 ± 0.028	0.116 ± 0.02	0.03 ± 0.015
0.75	0	0.25	148 ± 7	0 ± 0	22 ± 3	233 ± 2	0.619 ± 0.03	0.284 ± 0.033	0.098 ± 0.028
0.75	0	0	25 ± 4	0 ± 0	0 ± 0	231 ± 2	0.573 ± 0.038	0.322 ± 0.044	0.105 ± 0.036
0.25	1	1	25 ± 4	102 ± 0	199 ± 0	236 ± 0	0.953 ± 0.008	0.039 ± 0.01	0.008 ± 0.004
0.25	1	0.88	26 ± 5	102 ± 0	170 ± 4	236 ± 1	0.941 ± 0.01	0.047 ± 0.007	0.012 ± 0.008
0.25	1	0.75	26 ± 7	102 ± 0	139 ± 9	236 ± 0	0.934 ± 0.017	0.055 ± 0.017	0.011 ± 0.006
0.25	1	0.25	27 ± 4	102 ± 0	22 ± 3	232 ± 2	0.879 ± 0.013	0.086 ± 0.011	0.035 ± 0.009
0.25	1	0	24 ± 4	102 ± 0	0 ± 0	230 ± 2	0.872 ± 0.011	0.086 ± 0.007	0.042 ± 0.012
0.25	0.88	1	26 ± 5	89 ± 4	199 ± 0	236 ± 0	0.941 ± 0.007	0.051 ± 0.011	0.008 ± 0.007
0.25	0.88	0.88	24 ± 3	88 ± 3	171 ± 5	236 ± 0	0.928 ± 0.016	0.057 ± 0.015	0.015 ± 0.007
0.25	0.88	0.75	23 ± 4	89 ± 4	136 ± 6	235 ± 1	0.911 ± 0.018	0.066 ± 0.012	0.023 ± 0.012
0.25	0.88	0.25	25 ± 4	87 ± 3	22 ± 2	231 ± 2	0.817 ± 0.031	0.134 ± 0.023	0.049 ± 0.016
0.25	0.88	0	26 ± 5	86 ± 4	0 ± 0	227 ± 3	0.809 ± 0.031	0.127 ± 0.025	0.064 ± 0.013
0.25	0.75	1	25 ± 3	70 ± 3	199 ± 0	236 ± 1	0.922 ± 0.011	0.065 ± 0.01	0.014 ± 0.008
0.25	0.75	0.88	24 ± 6	71 ± 5	170 ± 5	236 ± 1	0.9 ± 0.022	0.085 ± 0.019	0.015 ± 0.006
0.25	0.75	0.75	25 ± 3	70 ± 5	138 ± 5	235 ± 1	0.869 ± 0.025	0.102 ± 0.022	0.03 ± 0.015
0.25	0.75	0.25	24 ± 4	70 ± 3	18 ± 2	228 ± 2	0.71 ± 0.028	0.216 ± 0.028	0.074 ± 0.017
0.25	0.75	0	22 ± 3	69 ± 4	0 ± 0	221 ± 3	0.687 ± 0.034	0.205 ± 0.027	0.108 ± 0.024
0.25	0.25	1	25 ± 4	12 ± 2	199 ± 0	235 ± 1	0.796 ± 0.025	0.169 ± 0.023	0.035 ± 0.014
0.25	0.25	0.88	24 ± 5	10 ± 2	171 ± 7	234 ± 1	0.709 ± 0.044	0.231 ± 0.037	0.06 ± 0.013
0.25	0.25	0.75	25 ± 2	10 ± 4	139 ± 6	233 ± 2	0.629 ± 0.029	0.316 ± 0.027	0.055 ± 0.01
0.25	0.25	0.25	24 ± 3	11 ± 3	20 ± 4	209 ± 5	0.324 ± 0.017	0.521 ± 0.03	0.155 ± 0.029
0.25	0.25	0	24 ± 4	10 ± 2	0 ± 0	178 ± 8	0.277 ± 0.018	0.594 ± 0.046	0.129 ± 0.034
0.25	0	1	24 ± 4	0 ± 0	199 ± 0	234 ± 1	0.652 ± 0.014	0.292 ± 0.014	0.056 ± 0.017
0.25	0	0.88	25 ± 4	0 ± 0	171 ± 4	233 ± 2	0.566 ± 0.034	0.365 ± 0.03	0.069 ± 0.023
0.25	0	0.75	25 ± 5	0 ± 0	133 ± 7	229 ± 2	0.431 ± 0.051	0.491 ± 0.05	0.078 ± 0.025
0.25	0	0.25	24 ± 5	0 ± 0	19 ± 3	188 ± 6	0.199 ± 0.033	0.701 ± 0.045	0.1 ± 0.025
0.25	0	0	0 ± 0	0 ± 0	0 ± 0	135 ± 11	0.155 ± 0.012	0.751 ± 0.022	0.094 ± 0.015
0	1	1	0 ± 0	102 ± 0	199 ± 0	236 ± 0	0.956 ± 0.003	0.039 ± 0.002	0.005 ± 0.002
0	1	0.88	0 ± 0	102 ± 0	171 ± 4	236 ± 1	0.938 ± 0.01	0.054 ± 0.008	0.008 ± 0.008
0	1	0.75	0 ± 0	102 ± 0	134 ± 6	235 ± 1	0.919 ± 0.012	0.066 ± 0.009	0.016 ± 0.008
0	1	0.25	0 ± 0	102 ± 0	22 ± 3	228 ± 2	0.849 ± 0.015	0.104 ± 0.006	0.047 ± 0.01
0	1	0	0 ± 0	102 ± 0	0 ± 0	219 ± 0	0.825 ± 0.009	0.111 ± 0.007	0.064 ± 0.007

0	0.88	1	0 ± 0	87 ± 3	199 ± 0	236 ± 0	0.941 ± 0.011	0.052 ± 0.007	0.008 ± 0.006
0	0.88	0.88	0 ± 0	87 ± 4	170 ± 6	236 ± 1	0.9 ± 0.013	0.074 ± 0.01	0.026 ± 0.015
0	0.88	0.75	0 ± 0	88 ± 3	136 ± 5	235 ± 1	0.902 ± 0.013	0.078 ± 0.006	0.02 ± 0.013
0	0.88	0.25	0 ± 0	88 ± 5	21 ± 7	224 ± 3	0.755 ± 0.018	0.171 ± 0.017	0.073 ± 0.015
0	0.88	0	0 ± 0	88 ± 2	0 ± 0	212 ± 2	0.713 ± 0.015	0.186 ± 0.011	0.102 ± 0.017
0	0.75	1	0 ± 0	72 ± 5	199 ± 0	235 ± 1	0.911 ± 0.022	0.077 ± 0.015	0.011 ± 0.009
0	0.75	0.88	0 ± 0	67 ± 6	170 ± 5	235 ± 1	0.873 ± 0.01	0.098 ± 0.009	0.028 ± 0.012
0	0.75	0.75	0 ± 0	68 ± 4	138 ± 7	234 ± 1	0.838 ± 0.031	0.125 ± 0.031	0.037 ± 0.016
0	0.75	0.25	0 ± 0	70 ± 3	21 ± 3	218 ± 3	0.638 ± 0.042	0.25 ± 0.035	0.112 ± 0.029
0	0.75	0	0 ± 0	70 ± 3	0 ± 0	200 ± 3	0.557 ± 0.026	0.298 ± 0.032	0.145 ± 0.035
0	0.25	1	0 ± 0	11 ± 3	199 ± 0	234 ± 1	0.686 ± 0.026	0.247 ± 0.029	0.066 ± 0.018
0	0.25	0.88	0 ± 0	10 ± 3	172 ± 2	232 ± 1	0.581 ± 0.033	0.342 ± 0.03	0.077 ± 0.016
0	0.25	0.75	0 ± 0	9 ± 2	138 ± 7	228 ± 3	0.461 ± 0.044	0.449 ± 0.028	0.09 ± 0.025
0	0.25	0.25	0 ± 0	10 ± 3	23 ± 4	170 ± 5	0.214 ± 0.025	0.671 ± 0.042	0.115 ± 0.033
0	0.25	0	0 ± 0	10 ± 3	0 ± 0	95 ± 7	0.159 ± 0.022	0.747 ± 0.037	0.094 ± 0.025
0	0	1	0 ± 0	0 ± 0	199 ± 0	232 ± 0	0.443 ± 0.017	0.528 ± 0.018	0.029 ± 0.006
0	0	0.88	0 ± 0	0 ± 0	170 ± 4	230 ± 1	0.319 ± 0.046	0.64 ± 0.035	0.041 ± 0.024
0	0	0.75	0 ± 0	0 ± 0	134 ± 6	225 ± 2	0.232 ± 0.039	0.724 ± 0.033	0.044 ± 0.016
0	0	0.25	0 ± 0	0 ± 0	22 ± 3	126 ± 7	0.119 ± 0.009	0.851 ± 0.011	0.03 ± 0.006

*Values are means and standard deviations from 10 independently generated data sets. Possible connectivities refer to the number of matching (i), (i-1) pairs of resonances of adjacent spin systems that are still present in the depleted data set. Entries of zero for error indicate that the standard deviation is less than the precision indicated for the observed value.
The table is included in Excel format in the Source Data file.

Supplementary Table 15 – Performance of BARASA using SHIFTX+ predicted shifts from AlphaFold2 models			
	**Fraction**		
	**Matching**	**Missing**	**Mismatching**
IL-1β	1.000	0.000	0.000
IL-1Ra	0.954	0.013	0.013
IGPS	0.899	0.098	0.004
MBP	0.984	0.003	0.013
CY1	0.927	0.046	0.026
ecTS	0.970	0.019	0.011
The table is included in Excel format in the Source Data file.			

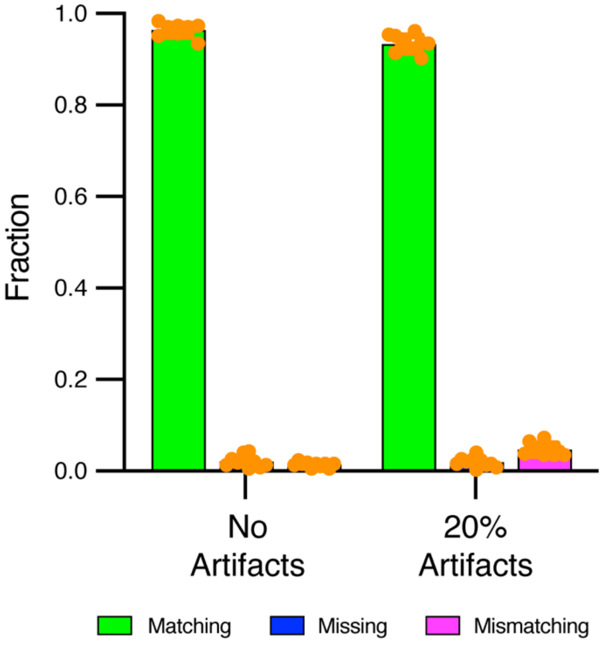

Supplementary Figure 1 | Performance of BARASA in the presence of artifact peaks. Depleted MBP data sets (88% CA, 25% CB, 75% CO) were augmented with randomly generated artifact peaks such that 20% of the total peaks in each peak list were artifacts. For each condition, ten independent data sets (n=10) were randomly generated containing the indicated amount of artifact peaks added. Results of BARASA on the individual data sets are shown as orange points. Bar heights represent the arithmetic mean of the data points. Source data are provided as a Source Data file.

References

1. Schleucher J., *et al.* A general enhancement scheme in heteronuclear multidimensional NMR employing pulsed field gradients. *J Biomol NMR* **4**, 301-306 (1994).
2. Bax A. & Ikura M. An efficient 3D NMR technique for correlating the proton and 15N backbone amide resonances with the α-carbon of the preceding residue in uniformly15N/13C enriched proteins. *J. Biomol. NMR.* **1**, 99-104 (1991).
3. Wittekind M. & Mueller L. HNCACB, a high-sensitivity 3D NMR experiment to correlate amide-proton and nitrogen resonances with the alpha- and beta-carbon resonances in proteins. *J. Magn. Reson. Ser B* **101**, 201-205 (1993).
4. Clubb R. T., Thanabal V. & Wagner G. A constant-time three-dimensional triple-resonance pulse scheme to correlate intraresidue 1HN, 15N, and 13C′ chemical shifts in 15N13C-labelled proteins. *J. Mang. Reson.* **97**, 213-217 (1992).
5. Grzesiek S. & Bax A. Improved 3D triple-resonance NMR techniques applied to a 31 kDa protein. *J. Magn. Reson.* **96**, 432-440 (1992).
6. Pervushin K., Riek R., Wider G. & Wüthrich K. Attenuated T2 relaxation by mutual cancellation of dipole-dipole coupling and chemical shift anisotropy indicates an avenue to NMR structures of very large biological macromolecules in solution. *Proc. Nat. Acad. Sci. USA* **94**, 12366-12371 (1997).
7. Hyberts S. G., Milbradt A. G., Wagner A. B., Arthanari H. & Wagner G. Application of iterative soft thresholding for fast reconstruction of NMR data non-uniformly sampled with multidimensional Poisson Gap scheduling. *J Biomol NMR* **52**, 315-327 (2012).
8. Han B., Liu Y., Ginzinger S. W. & Wishart D. S. SHIFTX2: Significantly improved protein chemical shift prediction. *J. Biomol. NMR.* **50**, 43-57 (2011).
9. Berman H. M., *et al.* The Protein Data Bank. *Nucleic Acid Res.* **28**, 235-242 (2000).
10. Lee W., *et al.* I-PINE web server: an integrative probabilistic NMR assignment system for proteins. *J. Biomol. NMR.* **73**, 213-222 (2019).
11. Schmidt E. & Güntert P. A new algorithm for reliable and general NMR resonance assignment. *J. Am. Chem. Soc.* **134**, 12817-12829 (2012).
12. Zimmerman D. E., *et al.* Automated analysis of protein NMR assignments using methods from artificial intelligence. *J. Mol. Biol.* **269**, 592-610 (1997).
13. Driscoll P. C., Marius Clore G., Marion D., Wingfield P. T. & Gronenborn A. M. Complete resonance assignment for the polypeptide backbone of interleukin 1ß using three-dimensional heteronuclear NMR spectroscopy. *Biochemistry* **29**, 3542-3556 (1990).
14. Sapienza P. J. & Lee A. L. Backbone and ILV methyl resonance assignments of E. coli thymidylate synthase bound to cofactor and a nucleotide analogue. *Biomol. NMR Assign.* **8**, 195-199 (2014).
15. Yang Y. & Igumenova T. I. The C-Terminal V5 domain of protein kinase Cα Is intrinsically disordered, with propensity to associate with a membrane mimetic. *PLoS ONE* **8**, 65699-65699 (2013).
