# Supplementary material for: Robust automated backbone triple resonance NMR assignments of proteins using Bayesian-based simulated annealing
Source: Nat Commun. 2023 Mar 21;14:1556. doi: 10.1038/s41467-023-37219-z (PMC10030768; doi:10.1038/s41467-023-37219-z)
Supplement: Supplementary file 2 — Reporting Summary [file 41467_2023_37219_MOESM2_ESM.pdf]

nature portfolio

Corresponding author(s): A. Joshua Wand

Last updated by author(s): Feb 19, 2023

Reporting Summary

Nature Portfolio wishes to improve the reproducibility of the work that we publish. This form provides structure for consistency and transparency in reporting. For further information on Nature Portfolio policies, see our Editorial Policies and the Editorial Policy Checklist.

Statistics

For all statistical analyses, confirm that the following items are present in the figure legend, table legend, main text, or Methods section.

n/a	Confirmed	
☐	☒	The exact sample size (n) for each experimental group/condition, given as a discrete number and unit of measurement
☐	☒	A statement on whether measurements were taken from distinct samples or whether the same sample was measured repeatedly
☐	☒	The statistical test(s) used AND whether they are one- or two-sided<br>*Only common tests should be described solely by name; describe more complex techniques in the Methods section.*
☒	☐	A description of all covariates tested
☒	☐	A description of any assumptions or corrections, such as tests of normality and adjustment for multiple comparisons
☐	☒	A full description of the statistical parameters including central tendency (e.g. means) or other basic estimates (e.g. regression coefficient) AND variation (e.g. standard deviation) or associated estimates of uncertainty (e.g. confidence intervals)
☐	☒	For null hypothesis testing, the test statistic (e.g. F, t, r) with confidence intervals, effect sizes, degrees of freedom and P value noted<br>*Give P values as exact values whenever suitable.*
☐	☒	For Bayesian analysis, information on the choice of priors and Markov chain Monte Carlo settings
☒	☐	For hierarchical and complex designs, identification of the appropriate level for tests and full reporting of outcomes
☒	☐	Estimates of effect sizes (e.g. Cohen's d, Pearson's r), indicating how they were calculated

Our web collection on statistics for biologists contains articles on many of the points above.

Software and code

Policy information about availability of computer code

Data collection	Data was collected using Topspin versions 3.6.2 and 4.1.2
Data analysis	Data processed using nmrPipe version 10.9 and HMS IST version 341. BARASA as described in the manuscript is version 1.2.0;PyMol 2.4.1; Boost: 1.74.0; WxWidgets: 3.1.4

For manuscripts utilizing custom algorithms or software that are central to the research but not yet described in published literature, software must be made available to editors and reviewers. We strongly encourage code deposition in a community repository (e.g. GitHub). See the Nature Portfolio guidelines for submitting code & software for further information.

Data

Policy information about availability of data

All manuscripts must include a data availability statement. This statement should provide the following information, where applicable:

- Accession codes, unique identifiers, or web links for publicly available datasets
- A description of any restrictions on data availability
- For clinical datasets or third party data, please ensure that the statement adheres to our policy

Resonance assignments for IGPS and IL-1Ra have been deposited to the BMRB under accession numbers 51347 and 51352, respectively.

Field-specific reporting

Please select the one below that is the best fit for your research. If you are not sure, read the appropriate sections before making your selection.

[x] Life sciences   [ ] Behavioural & social sciences   [ ] Ecological, evolutionary & environmental sciences

For a reference copy of the document with all sections, see nature.com/documents/nr-reporting-summary-flat.pdf

Life sciences study design

All studies must disclose on these points even when the disclosure is negative.

Sample size	There are two sample sizes - the number of independent simulated annealing runs that were executed to result in a consensus assignment set as explicitly defined in the text - twenty was generally found to be sufficient; the number of samplings of depleted input crosspeak data - ten was determined to adequately reveal the variation (which is very small and explictly shown in the dot plots)
Data exclusions	no data was excluded in the sense implied; the algorithm reported in this text was challenged by depleting the input data sets as explicitly described in the text
Replication	The input cross peak data sets were not replicated; the analysis was replicated (see sample size)
Randomization	Randomization is not relevant in the sense implied.
Blinding	No blinding was done. Not applicable since the analysis was done by machine algorithm.

Reporting for specific materials, systems and methods

We require information from authors about some types of materials, experimental systems and methods used in many studies. Here, indicate whether each material, system or method listed is relevant to your study. If you are not sure if a list item applies to your research, read the appropriate section before selecting a response.

Materials & experimental systems

n/a	Involved in the study
[x]	[ ] Antibodies
[x]	[ ] Eukaryotic cell lines
[x]	[ ] Palaeontology and archaeology
[x]	[ ] Animals and other organisms
[x]	[ ] Human research participants
[x]	[ ] Clinical data
[x]	[ ] Dual use research of concern

Methods

n/a	Involved in the study
[x]	[ ] ChIP-seq
[x]	[ ] Flow cytometry
[x]	[ ] MRI-based neuroimaging
